# Supplementary material for: Modelling the impact of lockdown-easing measures on cumulative COVID-19 cases and deaths in England
Source: BMJ Open. 2021 Sep 8;11(9):e042483. doi: 10.1136/bmjopen-2020-042483 (PMC8438582; doi:10.1136/bmjopen-2020-042483)
Supplement: Supplementary data [file bmjopen-2020-042483supp001.pdf]

SUPPLEMENTARY MATERIALS

Modelling the impact of lockdown easing measures on cumulative COVID-19 cases and deaths in England

Ziauddeen H, PhD [1,2,3], Subramaniam N, BSc [1,2], Gurdasani D, PhD† [4].

Supplementary Table 1: Comparison of Bayesian models with different constraints on changes in R_t

Model	RMSE	EPLD	SE	diff_EPLD_model3	diff_SE_model3
model 1	38.0	-505.1	25.0	-1.5	0.5
model 2	28.3	-504.8	24.5	-1.2	0.9
model 3	28.0	-503.6	24.8	NA	NA

Supp. Table 1 represents model comparisons between Models 1-3, as specified in the text. RMSE represents the Root mean squared error between estimated and observed deaths for each model. EPLD represents the expected log pointwise predictive density which approximates leave-one-out (LOO) cross-validation. Less negative scores suggest better fit. SE is the standard error of EPLD. We assess the difference in EPLD between all models and the best performing model (Model 3 in this case), comparing the difference in EPLD (diff_EPLD_model3) with the standard error of the difference (diff_SE_model3). Although all three models appear comparable in performance, Model 3 appears to show the best fit with the lowest RMSE, and the least negative EPLD.

Supplementary Table 2: Comparison of models excluding specific change points for R_t

change points removed	RMSE	EPLD	SE	diff_EPLD	diff_SE
16th March	118.1	-576.0	26.1	-70.9	5.3
23rd March	44.6	-506.7	25.2	-1.6	1.0
13th May	40.2	-504.8	25.0	0.3	0.4
1st June	38.1	-505.2	25.0	-0.1	0.0
None (all included)	38.0	-505.1	25.0	NA	NA

Supp. Table 2 represents model comparisons between Models that constrain Rt at each of the 4 hypothesised change points at which point social distancing or lockdown measures were introduced (16th March and 23rd March), or when lockdown measures were eased (13th May and 1st June). The first column represents the change point left out in each model, with the last model with all three change points being the comparator, as specified in the text. RMSE represents the Root mean squared error between estimated and observed deaths for each model. EPLD represents the expected log pointwise predictive density which approximates leave-one-out (LOO) cross-validation. Less negative scores suggest better fit. SE is the standard error of EPLD. We assess the difference in EPLD between all models and the model with all three change points, comparing the difference in EPLD (diff_EPLD) with the standard error of the difference (diff_SE). The model leaving out 16th March as a change point, i.e. constraining Rt to remain constant at this point appears to adversely impact fit the most.

Supplementary Table 3: Cumulative cases and deaths in lockdown easing scenarios in primary model

Rt 1st June	Rt 15th June	Rt 4th July	Cumulative cases	Cumulative deaths	Cases difference from baseline	Death difference from baseline	RMSE
0.752	0.752	0.752	4411594(4199223-4639250)	48501(46170-50989)	0(0,0)	0(0,0)	
0.6	0.6	0.6	4364386(4162299-4580834)	48006(45783-50386)	-44302(-84684--18600	-462(-884--194)	25.3
0.65	0.65	0.65	4374559(4170697-4593499)	48115(45875-50523)	-33831(-64668--14204)	-350(-669--147)	24.9
0.7	0.7	0.7	4391027(4183302-4610584)	48286(46007-50696)	-19968(-38168--8384)	-204(-389--86)	24.4
0.75	0.75	0.75	4410590(4198499-4637531)	48494(46163-50977)	-908(-1736--381)	-9(-17--4)	23.9
0.75	0.75	0.8	4415149(4201945-4645342)	48518(46186-51016)	3069(1285-5890)	19(8-37)	23.8
0.75	0.8	0.8	4424153(4209052-4658126)	48612(46255-51149)	11497(4814-22058)	102(43-195)	23.7
0.75	0.8	0.85	4430866(4213721-4668154)	48654(46293-51225)	18197(7620-34906)	145(61-278)	23.6
0.8	0.8	0.8	4439684(4219884-4679358)	48771(46375-51380)	26447(11105-50549)	257(108-492)	23.4
0.8	0.8	0.85	4447876(4225283-4692920)	48827(46413-51458)	34303(14397-65598)	308(129-589)	23.3
0.8	0.85	0.85	4461240(4232489-4716984)	48954(46492-51680)	47523(19934-90933)	431(181-825)	23.2
0.8	0.85	0.9	4474630(4243279-4736698)	49036(46538-51811)	60851(25519-116475)	508(213-972)	23.1
0.85	0.85	0.85	4481739(4247839-4745973)	49166(46614-52010)	67576(28376-129149)	632(265-1208)	23.0
0.85	0.85	0.9	4498639(4257710-4770487)	49246(46692-52138)	83109(34888-158891)	722(303-1379)	23.0
0.85	0.9	0.9	4521199(4273493-4806484)	49446(46808-52428)	104334(43782-199536)	907(381-1733)	23.1
0.85	0.9	0.95	4547931(4291851-4848863)	49592(46917-52640)	130823(54887-250256)	1043(438-1994)	23.2
0.9	0.9	0.9	4549190(4292969-4850945)	49730(47007-52865)	132381(55595-252977)	1173(493-2240)	23.3
0.9	0.9	0.95	4579138(4311424-4905124)	49887(47120-53118)	163082(68471-311726)	1330(559-2542)	23.5
0.9	0.95	0.95	4613802(4328984-4965451)	50162(47308-53602)	197988(83107-378532)	1610(676-3077)	24.3
0.9	0.95	1	4667450(4358502-5053567)	50397(47439-54007)	250499(105129-479023)	1848(776-3534)	25.2
0.95	0.95	0.95	4655308(4352263-5032669)	50517(47499-54226)	239051(100411-456749)	1971(828-3764)	25.3
0.95	0.95	1	4718132(4385399-5139609)	50790(47648-54680)	299596(125815-572543)	2246(943-4290)	26.6
0.95	1	1	4779022(4412370-5246023)	51226(47883-55381)	358354(150465-684935)	2672(1122-5106)	28.8
0.95	1	1.05	4880364(4464544-5437916)	51650(48103-56116)	462002(193954-883178)	3087(1296-5899)	31.8
1	1	1	4840595(4444280-5359379)	51743(48160-56290)	421310(177012-804811)	3174(1334-6060)	31.4
1	1	1.05	4959206(4498273-5586656)	52235(48368-57131)	540234(226938-1032145)	3649(1533-6970)	35.2
1	1.05	1.05	5055758(4548961-5767421)	52892(48712-58297)	641220(269327-1225202)	4303(1808-8220)	40.2
1	1.05	1.1	5264669(4632979-6143068)	53598(49059-59632)	844596(354706-1613979)	5018(2108-9587)	47.2
1.05	1.05	1.05	5156984(4594880-5946919)	53594(49059-59623)	741957(311832-1416940)	5017(2109-9578)	45.3
1.05	1.05	1.1	5397044(4692140-6391841)	54411(49421-61165)	974252(409410-1860772)	5833(2452-11138)	53.5
1.05	1.1	1.1	5574165(4770085-6732948)	55401(49905-63038)	1150799(483559-2198106)	6843(2876-13067)	62.8
1.05	1.1	1.15	5969381(4946974-7481001)	56609(50491-65190)	1546934(649955-2954983)	8065(3390-15402)	76.7
1.1	1.1	1.1	5744209(4843263-7044331)	56428(50400-64839)	1317940(554129-2516086)	7878(3313-15037)	71.5
1.1	1.1	1.15	6189495(5035202-7885232)	57860(50954-67461)	1768512(743504-3376542)	9269(3898-17692)	87.4
1.1	1.15	1.15	6501607(5163364-8475727)	59458(51650-70470)	2081127(874883-3973567)	10834(4556-20682)	103.2
1.1	1.15	1.2	7272289(5484637-9955130)	61543(52551-74465)	2846203(1196439-5434639)	12908(5427-24642)	128.7

Supplementary Table 3 represents the estimated cumulative deaths, cumulative cases, and excess deaths and cases in different scenarios of changing Rt at points of easing lockdown in comparison with the baseline scenario of Rt remaining constant at 0.752.

Supplementary Table 4: Cumulative cases and deaths in lockdown easing scenarios in model with long serial interval

Rt 1st June	Rt 15th June	Rt 4th July	Cumulative cases	Cumulative deaths	Cases difference from baseline	Death difference from baseline
0.691	0.691	0.691	4404236(4183512-4622330)	48411(45990-50805)	0(0-0)	0(0-0)
0.6	0.6	0.6	4371866(4158398-4583033)	48078(45733-50400)	-32402(-52309--17783)	-330(-533--181)
0.65	0.65	0.65	4387428(4171118-4603369)	48241(45863-50609)	-16515(-26661--9065)	-166(-268--91)
0.7	0.7	0.7	4408635(4186726-4627236)	48451(46022-50849)	4158(2283-6712)	41(23-66)
0.7	0.7	0.75	4413466(4190714-4632818)	48487(46047-50893)	8904(4865-14430)	75(41-121)
0.7	0.75	0.75	4421929(4197326-4644215)	48571(46126-50997)	17612(9599-28584)	160(87-259)
0.7	0.75	0.8	4428891(4202474-4652651)	48618(46159-51054)	24664(13432-40046)	206(112-334)
0.75	0.75	0.75	4436254(4207602-4661170)	48717(46226-51175)	31769(17441-51275)	307(168-494)
0.75	0.75	0.8	4444061(4214414-4673025)	48768(46266-51240)	39710(21767-64168)	358(197-579)
0.75	0.8	0.8	4456057(4225564-4689908)	48884(46358-51403)	51923(28426-84007)	473(259-765)
0.75	0.8	0.85	4468509(4233843-4705662)	48955(46423-51508)	63758(34875-103225)	544(298-881)
0.8	0.8	0.8	4474042(4238163-4713852)	49060(46515-51637)	69645(38242-112391)	652(358-1051)
0.8	0.8	0.85	4487239(4247137-4731986)	49147(46570-51756)	82925(45486-133921)	732(402-1180)
0.8	0.85	0.85	4504368(4260726-4758965)	49297(46674-51983)	100288(54965-162081)	887(487-1433)
0.8	0.85	0.9	4524968(4276399-4786847)	49405(46756-52128)	120111(65789-194244)	997(546-1610)
0.85	0.85	0.85	4528097(4278796-4789875)	49515(46843-52296)	122997(67554-198451)	1107(609-1785)
0.85	0.85	0.9	4550519(4295317-4824987)	49639(46934-52475)	145167(79670-234360)	1229(675-1983)
0.85	0.9	0.9	4575600(4314157-4857930)	49864(47104-52757)	170201(93348-274900)	1441(791-2326)
0.85	0.9	0.95	4608999(4336767-4903946)	50032(47229-53038)	203356(111471-328606)	1608(883-2597)
0.9	0.9	0.9	4605943(4334310-4898052)	50138(47312-53190)	200055(109912-322694)	1716(944-2766)
0.9	0.9	0.95	4643792(4360620-4952873)	50326(47454-53431)	237006(130136-382485)	1902(1046-3067)
0.9	0.95	0.95	4680570(4385671-5007504)	50621(47645-53806)	273569(150145-441642)	2192(1204-3535)
0.9	0.95	1	4735012(4422134-5091796)	50870(47843-54169)	328896(180416-531139)	2446(1343-3946)
0.95	0.95	0.95	4720210(4412771-5069359)	50965(47911-54323)	313876(172518-506126)	2540(1397-4093)
0.95	0.95	1	4781115(4451857-5160478)	51266(48080-54750)	375333(206196-605461)	2822(1552-4549)
0.95	1	1	4836432(4486836-5241471)	51661(48368-55340)	429433(235844-692907)	3219(1770-5191)
0.95	1	1.05	4928929(4549055-5385960)	52032(48613-55941)	521422(286255-841597)	3603(1980-5811)
1	1	1	4892268(4526735-5326989)	52097(48655-56020)	485265(266854-782184)	3667(2018-5906)
1	1	1.05	4995150(4589022-5491882)	52523(48926-56643)	587186(322775-946757)	4092(2252-6593)
1	1.05	1.05	5076092(4640904-5614490)	53078(49274-57480)	668188(367218-1077551)	4639(2552-7476)
1	1.05	1.1	5227156(4734604-5849954)	53653(49678-58359)	820638(450864-1323717)	5217(2869-8409)
1.05	1.05	1.05	5154698(4689409-5733251)	53657(49684-58359)	747364(411252-1204170)	5221(2876-8408)
1.05	1.05	1.1	5321327(4792479-5997797)	54309(50099-59370)	915842(503787-1475921)	5860(3227-9437)
1.05	1.1	1.1	5444111(4858418-6188703)	55069(50551-60585)	1038372(571090-1673570)	6615(3642-10657)
1.05	1.1	1.15	5698707(4995152-6588038)	55936(51099-61889)	1290002(709312-2079494)	7480(4117-12052)
1.1	1.1	1.1	5558967(4921706-6371716)	55831(51060-61736)	1153037(634971-1857036)	7382(4069-11884)
1.1	1.1	1.15	5838364(5075136-6811946)	56779(51632-63173)	1430388(787494-2304058)	8336(4593-13420)
1.1	1.15	1.15	6021434(5176394-7116504)	57812(52276-64824)	1617286(890276-2605292)	9384(5170-15109)
1.1	1.15	1.2	6430941(5404442-7783304)	59109(53001-66839)	2030763(1117659-3271715)	10673(5879-17187)

Supplementary Table 4 represents the estimated cumulative deaths, cumulative cases, and excess deaths and cases in different scenarios of changing Rt at points of easing lockdown in comparison with the baseline scenario of Rt remaining constant at 0.691.

Supplementary Figure 1: Distribution of R-hat for parameters from final model

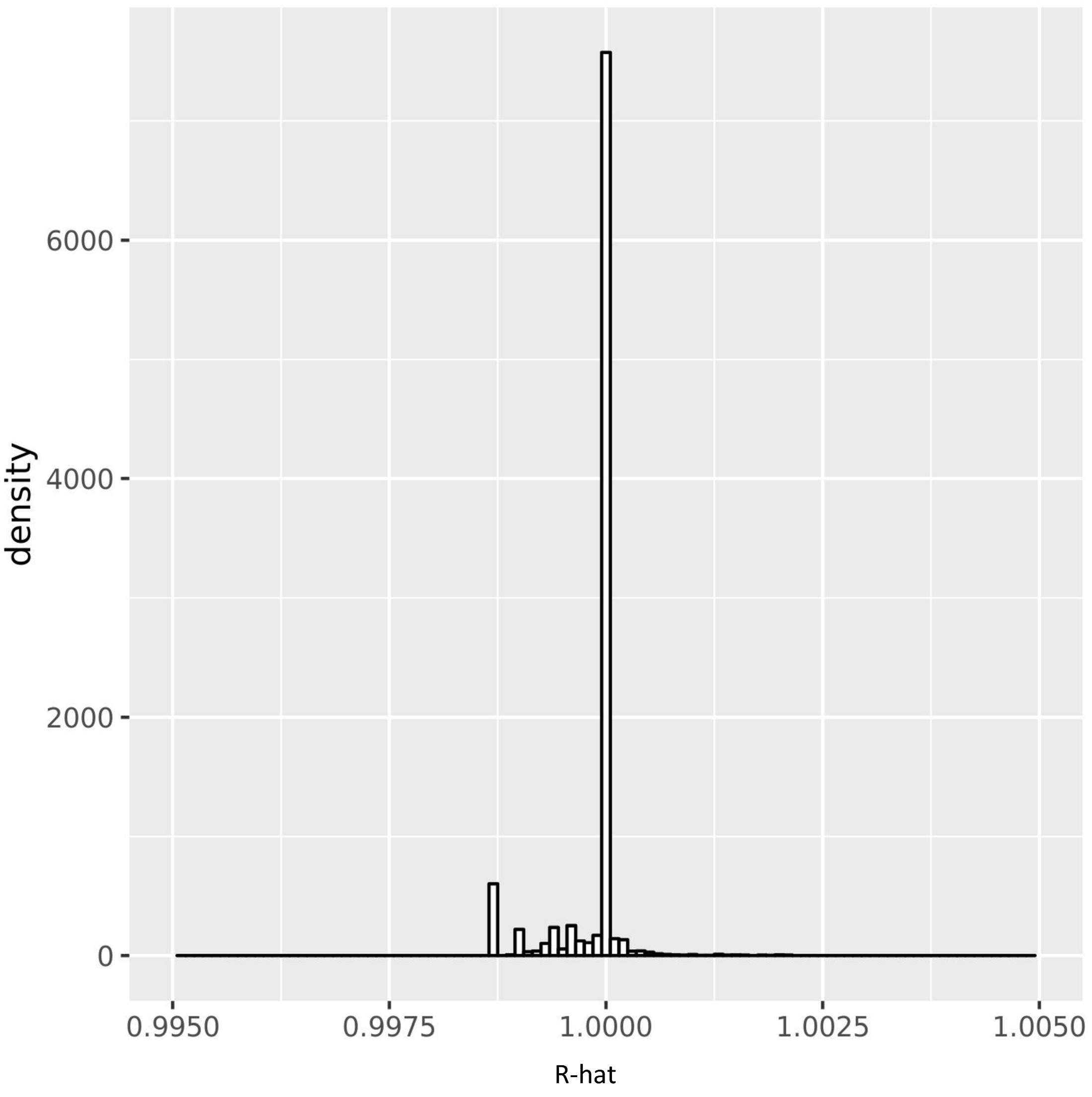

Supplementary Figure 1 represents the estimated R-hat for parameters of the final model with duplicates removed. The mean R-hat was 1.000057. An R-hat near 1 suggests that between-chain variance for a given parameter is equal to the within-chain variance, suggesting convergence of the model. All values were well below 1.05.

Supplementary Figure 2: Pareto shape parameter k distribution for final model

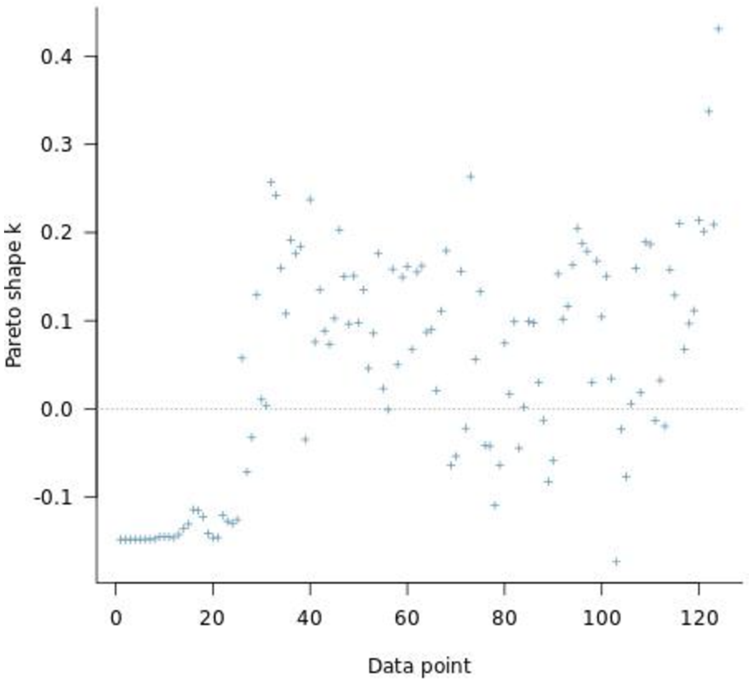

The estimated shape parameter k of the generalized Pareto distribution can be used to assess the reliability of the estimate from approximations of Leave-one-out cross-validation (LOO). The k shape values are all below 0.5, suggesting our estimates are reliable.

Supplementary Figure 3: Rt estimates with broad and uninformative priors

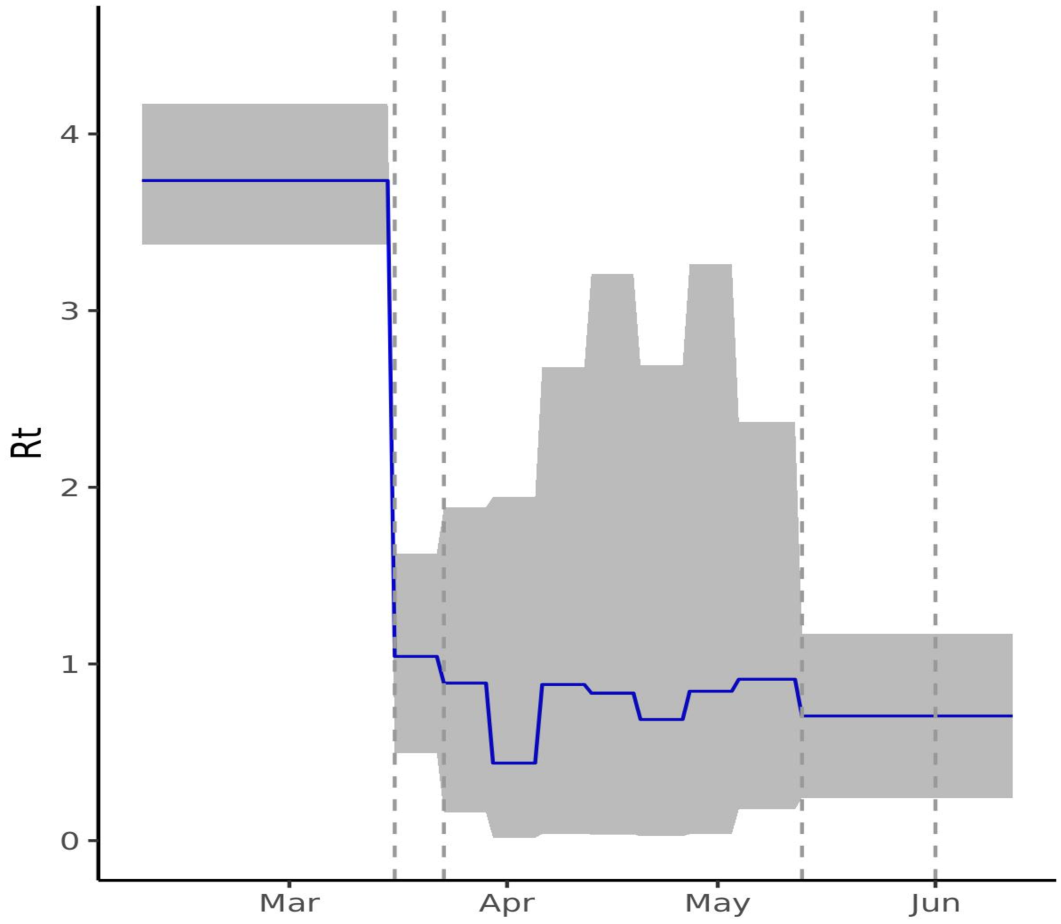

Supplementary Figure 3. Represents estimates of Rt when uninformative priors are used for estimation. We find that although uncertainty is greater around estimates, median estimates, and patterns of changes are similar as for the original model for all time intervals, suggesting that these are not constrained by specification of the prior in the final model.

Supplementary Figure 4: Estimated reproduction number in model with longer serial interval

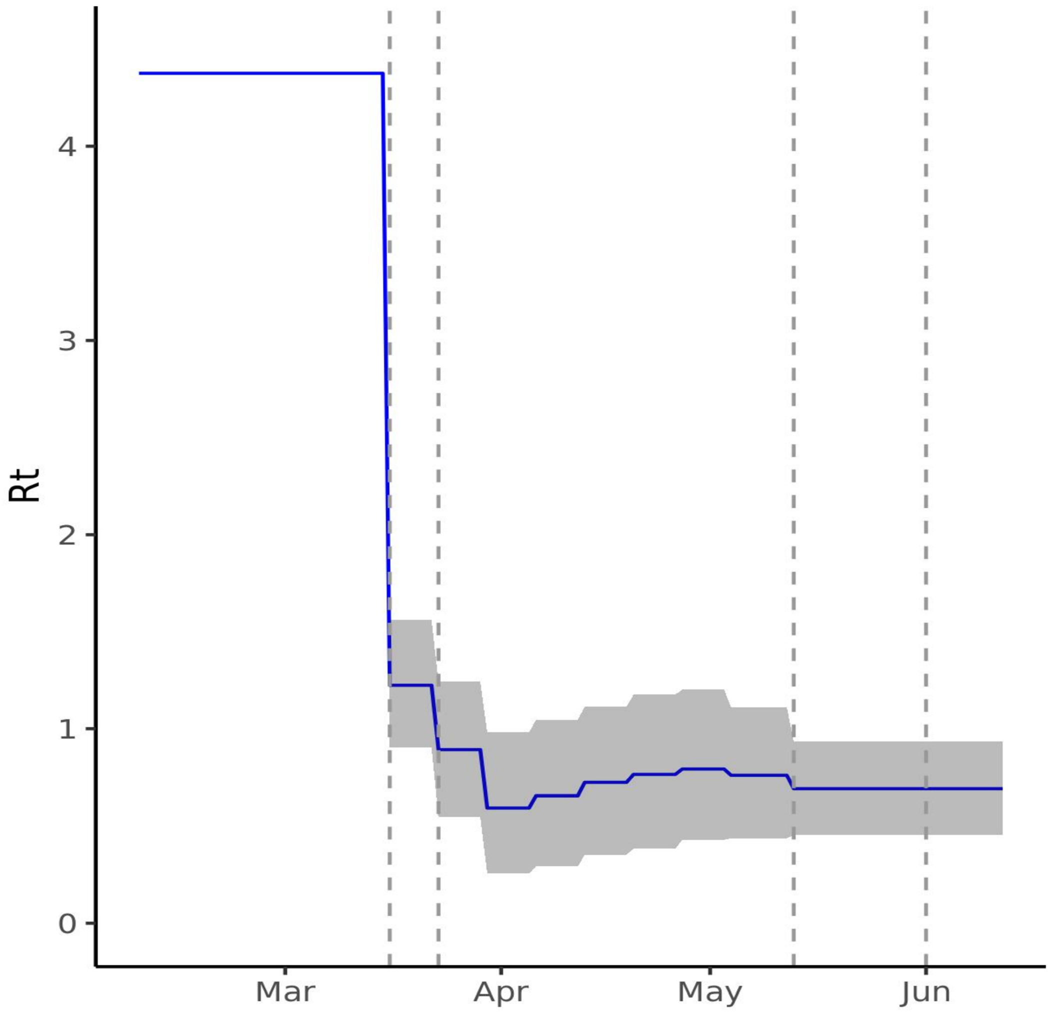

The figure shows the R_t estimated by a model with a a serial interval of mean 6.5 and coefficient of variation of 0.72. While estimates of R0 are higher in this model, estimates during other time intervals following lockdown are very similar to our primary model. 95% credible intervals are represented by grey bands.

BMJ Publishing Group Limited (BMJ) disclaims all liability and responsibility arising from any reliance placed on this supplemental material which has been supplied by the author(s)

Supplementary Figure 5: Predicted and observed deaths in model with longer serial interval

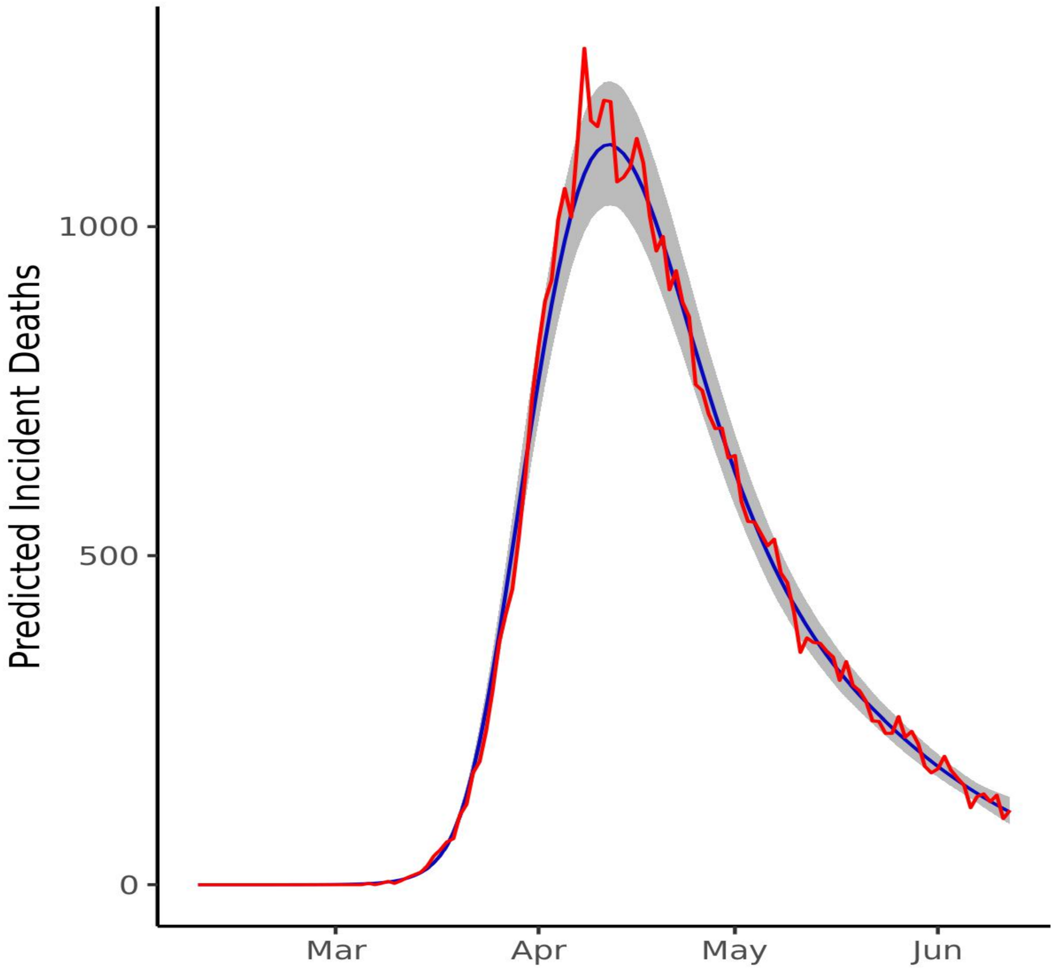

Daily deaths predicted by a model specifying longer serial intervals (blue) with 95% credible intervals (grey) show a good fit to the observed deaths from the ONS (red)
