# Supplementary material for: Modelling the impact of lockdown-easing measures on cumulative COVID-19 cases and deaths in England
Source: BMJ Open. 2021 Sep 8;11(9):e042483. doi: 10.1136/bmjopen-2020-042483 (PMC8438582; doi:10.1136/bmjopen-2020-042483)
Supplement: Author's manuscript [file bmjopen-2020-042483.draft_revisions.pdf]

BMJ Open is committed to open peer review. As part of this commitment we make the peer review history of every article we publish publicly available.

When an article is published we post the peer reviewers' comments and the authors' responses online. We also post the versions of the paper that were used during peer review. These are the versions that the peer review comments apply to.

The versions of the paper that follow are the versions that were submitted during the peer review process. They are not the versions of record or the final published versions. They should not be cited or distributed as the published version of this manuscript.

BMJ Open is an open access journal and the full, final, typeset and author-corrected version of record of the manuscript is available on our site with no access controls, subscription charges or pay-per-view fees (http://bmjopen.bmj.com).

If you have any questions on BMJ Open's open peer review process please email info.bmjopen@bmj.com

BMJ Open

Modelling the impact of lockdown easing measures on cumulative COVID-19 cases and deaths in England

Journal:	*BMJ Open*
Manuscript ID	bmjopen-2020-042483
Article Type:	Original research
Date Submitted by the Author:	07-Jul-2020
Complete List of Authors:	Ziauddeen, Hisham; University of Cambridge<br>Subramaniam, Naresh; University of Cambridge<br>Gurdasani, Deepti; Queen Mary University of London, William Harvey Research Institute
Keywords:	Epidemiology < TROPICAL MEDICINE, EPIDEMIOLOGY, Health policy < HEALTH SERVICES ADMINISTRATION & MANAGEMENT, Public health < INFECTIOUS DISEASES, Infection control < INFECTIOUS DISEASES

SCHOLARONE™
Manuscripts

I, the Submitting Author has the right to grant and does grant on behalf of all authors of the Work (as defined in the below author licence), an exclusive licence and/or a non-exclusive licence for contributions from authors who are: i) UK Crown employees; ii) where BMJ has agreed a CC-BY licence shall apply, and/or iii) in accordance with the terms applicable for US Federal Government officers or employees acting as part of their official duties; on a worldwide, perpetual, irrevocable, royalty-free basis to BMJ Publishing Group Ltd ("BMJ") its licensees and where the relevant Journal is co-owned by BMJ to the co-owners of the Journal, to publish the Work in this journal and any other BMJ products and to exploit all rights, as set out in our licence.

The Submitting Author accepts and understands that any supply made under these terms is made by BMJ to the Submitting Author unless you are acting as an employee on behalf of your employer or a postgraduate student of an affiliated institution which is paying any applicable article publishing charge ("APC") for Open Access articles. Where the Submitting Author wishes to make the Work available on an Open Access basis (and intends to pay the relevant APC), the terms of reuse of such Open Access shall be governed by a Creative Commons licence – details of these licences and which Creative Commons licence will apply to this Work are set out in our licence referred to above.

Other than as permitted in any relevant BMJ Author's Self Archiving Policies, I confirm this Work has not been accepted for publication elsewhere, is not being considered for publication elsewhere and does not duplicate material already published. I confirm all authors consent to publication of this Work and authorise the granting of this licence.

For peer review only

Modelling the impact of lockdown easing measures on cumulative COVID-19 cases and deaths in England

Ziauddeen H, PhD [1,2,3], Subramaniam N, BSc [1,2], Gurdasani D, PhD† [4].

[1] Dept. of Psychiatry, University of Cambridge, Cambridge UK

[2] Wellcome Trust-MRC Institute of Metabolic Science, University of Cambridge, Cambridge UK

[3] Cambridgeshire & Peterborough Foundation Trust, Cambridge UK

[4] William Harvey Research Institute, Barts and The London School of Medicine and Dentistry, Queen Mary University of London, London UK

† **Corresponding author**:

Dr Deepti Gurdasani
William Harvey Research Institute,
Barts and The London School of Medicine and Dentistry,
Queen Mary University of London, London UK
Email: d.gurdasani@qmul.ac.uk
Phone: 07737650357

For peer review only

Abstract:

Objectives:
To assess the potential impacts of successive lockdown easing measures in England, at a point in the COVID-19 pandemic when community transmission levels were relatively high. We specifically focus on scenarios where the reproductive number (*R*) remains ≤1 in line with the UK government's stated aim.

Design:
We developed a Bayesian model to infer incident cases and *R* in England, from incident death data. We then used this to forecast excess cases and deaths in multiple plausible scenarios in which *R* increases at one or more time points.

Setting:
England

Participants:
Publicly available national incident death data for COVID-19 were examined.

Primary Outcome:
Excess cumulative cases and deaths forecast at 90 days, in simulated scenarios of plausible increases in *R* as a result of successive easing of lockdown in England, compared to a baseline scenario where *R* remained constant.

Results:
Our model inferred an *R* of 0.75 on the 13th May when England first started easing lockdown. In the most conservative scenario where *R* increases to 0.80 as lockdown was eased further on 1st June and then remained constant, the model predicts an excess 257 (95% 108-492) deaths and 26,447 (95% CI 11,105-50,549) cumulative cases over 90 days. In the scenario with maximal increases in *R* (but staying ≤1) with easing of lockdown, the model predicts 3,174 (95% CI 1,334-6,060) excess cumulative deaths and 421,310 (95% CI 177,012-804,811) excess cases.

Conclusions:
When levels of transmission are high, even small changes in *R* with easing of lockdown can have significant impacts on expected cases and deaths, even if *R* remains ≤1. This will have a major impact on population health, tracing systems and health care services in England. Following an elimination strategy rather than one of maintenance of *R*≤1 would substantially mitigate the impact of the COVID-19 epidemic within England.

Strengths and limitations

1. This study provides urgently needed information about the potential impact of successive lockdown easing measures in England when community transmission of SARS-CoV2 is relatively high.

For peer review only

2. We utilise a robust Bayesian model based on ONS registered deaths in England, to infer incident cases and reproduction number and then forecast deaths and cases considering multiple plausible scenarios of increase in reproduction number with successive easing of lockdown in England.
3. Our study focuses on the impact of easing lockdown in the conservative scenario when R is maintained at or below 1 in line with stated government policy, showing that even this scenario would result in substantial excess of cases and deaths relative to a baseline scenario of not easing lockdown or elimination.
4. The excess cumulative cases are likely to be sensitive to the specified infection fatality ratio, although this is not expected to materially change are results and inferences.
5. The model inference in dependent on reliable reported statistics on incident deaths. Underestimation of recent registered deaths would lead to more conservative R inference, and underestimation of the impact of easing lockdown.

Introduction:

As countries around the world negotiate the first wave of the COVID-19 pandemic, governments have had to make critical decisions about when and how they ease the lockdown measures that were instituted to control the pandemic. Given the risks of a resurgence of the pandemic and the consequent implications, these decisions need to be informed by best available scientific evidence available at the time.

Different countries have eased lockdown in different ways, and at different points in their epidemic trajectory.[1] The UK imposed lockdown relatively late in its epidemic trajectory and began easing lockdown relatively early, when community transmission levels (incident cases) were still high.[2] By contrast, Germany, Denmark, Italy and Spain started easing lockdown when incident cases and deaths were at much lower levels. Despite mitigating strategies such as test, trace and isolation systems in place, countries like Germany have seen increases in reproduction number (*R)* after easing lockdown, with increases to above 1 in June.[3] South Korea, and China have also recently seen a resurgence in new cases, leading to new localised restrictions being put in place to control the spread of infections.

Easing lockdown when community transmission remains high likely increases the risk of a resurgence of the epidemic but the more precise impacts are insufficiently understood. Several experts, including SAGE, the scientific advisory body to the UK government, cautioned against easing lockdown at this point,[2] warning that the testing and contact tracing services that are meant to mitigate the impact of easing lockdown, could be overwhelmed and the health service greatly impacted. Nevertheless, the UK has proceeded with easing lockdown with the stated aim of doing so while keeping $R \leq 1$. On the 13th May, people who could not work from home were asked to return to work. On the 1st June schools were re-opened, outdoor markets and showrooms opened and households were allowed to meet in socially distanced groups of six. On the 15th June non-essential businesses, including the retail sector, were opened. On the 4th of July, pubs, cafes, and hotels are due to open. However in the week of the 29th June, a surge in cases was reported in Leicester, England, leading to the re-imposition of restrictive measures, and concern that other regions in England may experience

For peer review only - http://bmjopen.bmj.com/site/about/guidelines.xhtml

similar increases in case numbers.[4] As of now the government are proceeding with their proposed plan for the 4th July.

Understanding and quantifying the potential impact of lockdown easing measures at this point is crucial to informing public health strategy within England. Here, we model these impacts across a range of plausible scenarios. We use an epidemiological model of COVID-19 spread with Bayesian inference to infer parameters of the epidemic within England using daily death data from the Office of National Statistics (ONS). We estimate the time varying *R* and daily cases, and then use these to forecast cases and deaths in several plausible scenarios in which *R* increases as a result of easing lockdown, particularly focusing on scenarios in which *R* remains ≤1, and contrasting these with elimination strategies that aim to suppress *R* as much as possible.

Methods

Data for model development:

In order to model the impact of easing lockdown, we need to know the current levels of transmission, and growth parameters of the regional epidemic. Given the limited community testing and case detection in the UK, incident case numbers are likely to be substantially underestimated. We therefore based our model on the number of incident deaths by date of occurrence, which are likely to be more reliable.[5] Incident deaths are a function of incident cases in the previous weeks and the reproduction rate of the epidemic, and both these parameters can be inferred from the death data.[5] We included data till the 12th of June for England, as released by the ONS on the 30th of June 2020 (25th week of published data).[6] These data are based on deaths registered by the 27th of June. As reporting delays mean that more recent deaths are underestimated, we only considered deaths up to the 12th June.

Patient and public involvement

As only publicly available aggregate incident death statistics were utilised, there was no direct patient or public involvement.

Primary outcomes:

We assessed the excess cumulative predicted cases and deaths, over a 90-day period from the 1st June. We assumed different scenarios of changing *R* at the points of lockdown easing, in comparison with a baseline scenario in which *R* remained constant during this period.

Estimation of incident cases:

Incident cases, and time-varying *R* numbers were estimated using a Bayesian model, similar to that previously described by Flaxman et al,[5] accounting for the delay between onset of infection and death. The number of infected individuals is modelled using a discrete renewal process, as has been described before.[5] This is related to the commonly used Susceptible-Infected-Recovered (SIR) model, but is not expressed in differential form.

We modelled cases from 30 days prior to the first day that 10 cumulative deaths were observed in England, similar to previous methods.[5] The numbers of incident cases for the first 6 days of this period were set as parameters to be estimated by the model (**Supplementary Table 1**). Subsequent incident case numbers would then be a function of these initial cases,

For peer review only - http://bmjopen.bmj.com/site/about/guidelines.xhtml

and estimated R values. We assumed a serial interval (SI) with a lognormal distribution with mean 4.7 and standard deviation (SD) of 2.9 days, as in Nishiura et al [7]. The SI was discretised as follows:

$$g_s = \int_{t=s-1}^{s} g(t)dt$$

For s=1,2…N, where N is the total number of intervals (each interval being 1 day) estimated. We estimated the distribution for 201 days, to align with the 111 days of data up to the 29th May, plus 90 days of forecasting. Given a SI distribution, the number of infections c_t on a given day t, is given by the following discrete convolution function:

$$c_t = R_t \sum_{j=0}^{t-1} c_j g_{t-j},$$

The incident cases on a given day t, are therefore a function of R at point t and incident cases up to time $t-1$, weighted by the distribution of the serial interval.

Estimation of time-varying reproduction number

The baseline reproduction number (R_0), and the subsequent time varying effective reproduction number (R_t) were estimated up to the 12th June. We allowed R_t to change on at least three points: (1) 16th March, when the UK first introduced social distancing measures; (2) 23rd March, when lockdown measures came into place with stay at home instructions and closures of schools and non-essential businesses; and (3) 13th May, the first easing of lockdown. We also considered models in which R_t was allowed to change on the 1st June. Given the limited death data i.e. only up to the 12th June, we were unlikely to be able to estimate changes in R_t after the 13th May with sufficient certainty. Observed deaths from the 1st June are likely to be a function of cases 2-3 weeks prior to this, and were unlikely to reflect changes in R_t from the 1st of June.

Model selection

We assessed and compared models that allowed R_t to change at the 4 points described above (Model 1), with more flexible models that allowed more frequent changes (Models 2 and 3), as follows:

1. Model 1: 16th March, 23rd March, 13th May and 1st June
2. Model 2: Every week from the beginning of the modelling period, including on the 16th March, 23rd March, 13th May and the 1st June
3. Model 3: 16th March, 23rd March, and 13th May, and every week between the 23rd March and 13th May i.e. during lockdown.

For each model, we used the R package *loo* to calculate expected log pointwise predictive density (ELPD) using Leave-one-out cross-validation (LOO) individually for each left out data point based on the model fit to the other data points. We then calculated between-model differences in ELPDs, to assess whether particular models predicted data better than others, as discussed previously.[8] As the assumptions in estimation of ELPD may be violated given these are time-series data, and therefore correlated, we also compared the root mean

For peer review only

squared errors (RMSE) across models to assess fit. The final model used was arrived upon based on these comparisons.

In addition, we also compared Model 1 (four change points) with models where each of the change points were left out in turn, as done by Dehnig et al,[9] to assess if these dates do correspond to change points in R_t.

Estimation of deaths:
Incident deaths from COVID-19 are a function of the infection fatality rate (IFR), the proportion of infections that result in death, and incident cases that have occurred over the past 2-3 weeks. For observed daily deaths (D_t) for days $t \in 1, \ldots, n$, the expectation of observed daily deaths (d_t) is given by:

$$d_t = \mathrm{E}(D_t)$$

As described in Flaxman et al., we model the number of observed daily deaths D_t as following a negative binomial distribution with mean d_t and variance $d_t + \frac{d_t^2}{\psi}$, where ψ follows a half normal distribution:

$$D_t \sim Negative\ Binomial\ \left(d_t,\ d_t + \frac{d_t^2}{\psi}\right), \qquad \text{where } \psi \sim Normal^{+}(0,5).$$

Similar to estimation of incident cases, deaths at time point t (d_t) were modelled as a function of incident cases up to time $t{-}1$, weighted by the distribution of time of infection to time of death (π). The π distribution was modelled as the sum of the distribution of infection onset to symptom onset (the incubation period), and the distribution of symptom onset to death. As has been previously done,[5] both of these were modelled as gamma distributions with means of 5.1 days (coefficient of variation 0.86) and 18.8 days (coefficient of variation 0.45), respectively as follows:

$$\pi \sim IFR * (Gamma(5.1, 0.86) + Gamma(18.8, 0.45))$$

IFR was assumed to be 1.1%, based on the most recent estimates from the University of Cambridge MRC Nowcasting and Forecasting model.[10]

To discretise this distribution, we estimated the probability of death within each discrete time interval (1 day), conditional on surviving previous intervals. First we calculate the hazard (h_t) the instantaneous probability of failure (i.e. dying) within a time interval, as follows:

$$h_t = \frac{\int_{t\,=\,s\,-\,0.5}^{s\,+\,0.5} \pi(t)dt}{1 - \pi_{s\,-\,0.5}}$$

As the denominator excludes individuals who have died, this ensures that h_t is calculated only among those surviving. The probability of survival within each interval is:

$$s_t = 1 - h_t$$

For peer review only - http://bmjopen.bmj.com/site/about/guidelines.xhtml

The cumulative survival probability of surviving up to the interval $t-1$ is therefore:

$$S_{T>t-1} = \prod_{j=1}^{t-1} s_j$$

, where T is the time of death of an individual. In other words the cumulative probability of survival up to interval t is simply the product of survival within each interval up to $t-1$, where the probability of survival within each interval (s_t) is $1-h_t$, where h_t is the probability of dying within that interval.

Given this, we now estimate the probability of death within interval t, conditional on surviving up to $t-1$ as:

$$\omega_t = P(T = t \mid T > t-1) = S_{T>t-1} * h_t$$

Here ω represents the discretised distribution of infection onset to death, with the probability of death within interval t conditional on surviving previous intervals. Deaths can therefore be calculated as a function of incident cases of infection within previous intervals, as follows:

$$d_t = \sum_{j=0}^{t-1} c_j \omega_{t-j}$$

Here, the number of deaths within interval t (on a given day) is a sum of the number of daily cases up to the previous day, with previous cases weighted by the discretised probability distribution of time from onset of infection to death.

Estimated parameters and model priors:
We estimated the set of model parameters θ={$c_{1\text{-}6}$, R_0, R_t, φ, $\boldsymbol{\tau}$} using Bayesian inference with Markov-chain Monte-Carlo (MCMC) (**Supplementary Table 1**). We estimated the number of cases in the first six days of the modelled period, as subsequent cases are simply a function of cases on these days, the SI, and R_t . As described above, R_0 was constrained up to the 16th March and then again after the 13th of May. For the period prior to 16th March, we assigned a normal prior for R_0 with mean 2.5 and SD 0.5. For the period that R_t was allowed to vary i.e. every week from the 16th of March till the 13th of May, we assigned a normal prior with a mean 0.8 and SD 0.25. These priors are based on estimates of time changing R_t from the University of Cambridge MRC biostatistics nowcasting and forecasting models[10] and SAGE estimates of R,[11] and consistent with Flaxman et al.[5] For the number of cases on day 1, we assigned a prior exponential distribution:

$$y \sim exponential\left(\frac{1}{\tau}\right)$$

where $$\tau \sim exponential(0.03)$$

Model estimation:
Parameters were estimated using the Stan package in R with Markov chain Monte Carlo (MCMC) algorithms used to approximate a posterior distribution of parameters by randomly sampling the parameter space. We used 4 chains with 1000 warm up samples (which were

For peer review only - http://bmjopen.bmj.com/site/about/guidelines.xhtml

discarded), and 3000 subsequent samples in each chain (12,000 samples in total) to approximate a posterior distribution using the Gibbs Sampling algorithm. From these we obtained the best-fit values and the 95% credible intervals for all parameters. We used these parameters to estimate the number of incident cases and deaths in England. We examined the fit of the model predicted deaths to the observed daily deaths from the ONS, and also the consistency of the model parameters with known values in the literature, estimated from global data. We assessed the distribution of *R-hat* values for all parameters, to assess convergence between chains.

Sensitivity analyses:

We carried out sensitivity analyses using broader, and uninformative priors for R_0 and R_t, to examine the sensitivity of R_t estimates to prior specification. We also examined the impact of the SI by comparing the baseline model (SI of mean 4.7 and SD 2.9 days), with a longer SI modelled as a gamma distribution with mean 6.5 and coefficient of variation of 0.72, as estimated by Chan et al.[12]

Forecasting cases and deaths:

All forecasts were carried out up to 90 days (29th August 2020) after the 1st of June. We considered a set of scenarios in which R_t increased from baseline on the 1st of June and then remained constant, as well as those in which further increases in R_t occur on the 15th June and the 4th July (**Figures 3a, 4a** and **5a**). We considered an increase in R_t of up to 0.25 in increments of 0.05, this being a plausible degree of change in response to easing lockdown, based on the empirical data from other countries,[3,13] as well as the modelling by UK SAGE.[14] Finally, for comparison with a strategy of elimination, namely suppressing R_t to the lowest level possible before easing lockdown measures, as has been done South Korea, New Zealand and Australia, we also modelled scenarios with R_t values of 0.6 and 0.7.

For each of these scenarios, we predicted the number of incident cases, and incident deaths, using the functions from the inference model above. Briefly cases are a function of R_t, incident cases on previous days and the SI discretised distribution:

$$c_t = R_t \textstyle\sum_{j=0}^{t-1} c_j g_{t-j},$$

Deaths are a function of incident cases over previous weeks, and the distribution of onset of infection to death times:

$$d_t = \sum_{j=0}^{t-1} c_j \omega_{t-j}$$

All scenarios were compared to a baseline scenario of no change in R_t from the 13th of May onwards.

Results

Model selection and model inferences

Model 3, which allowed weekly changes in R_t during lockdown, produced the best fit to the data (**Supplementary Table 2**), with estimation of fewer parameters compared with Model 2.

For peer review only - http://bmjopen.bmj.com/site/about/guidelines.xhtml

This was therefore used as the primary model and unless otherwise stated, all inferences described subsequently are from this model.

We infer R_0 of 3.65 (95% credible intervals (CI) 3.36-3.96), consistent with previous estimates within the UK.[5] The R_t is estimated to have declined substantially following initiation of social distancing, and lockdown measures, reaching a low of 0.66 (95% CI 0.34-1.04) during the week 30th March-5th April 2020. The most recent R_t from the 13th of May is estimated as 0.752 (95% CI 0.50-1.00) (**Figure 1**). The alternative models allowing change of R_t on the 1st of June inferred a very similar R_t for the 1st -12th June suggesting that there was insufficient data to accurately infer any changes to R_t following the easing of lockdown on 1st June. On examining the impact of constraining R_t on model fit at any of the 4 change points, this appears greatest for the 16th March (when social distancing measures were put into place) (**Supplementary Table 3**) with only modest impacts on model fit of constraining R_t on 23rd March and 13th May, and no impact on constraining R_t on the 1st June.

The model showed a good fit to the observed distribution of deaths up to the 12th June (**Figure 2**). *Rhat* estimates were < 1.05 for all estimated parameters (**Supplementary Figure 1**). Leave one out cross-validation also supported a good model fit, with the shape parameter k<0.5 for all values (**Supplementary Figure 2**). The median number of incident cases inferred on the 1st June was 4,317/day (95% CI 2,062-8,155), which is broadly consistent with the estimates from the ONS survey for England based on a random sample of the population within the same time period.

Forecasts of lockdown easing scenarios

In the baseline forecasting scenario where R_t remains constant (Rt_{est}=0.75) through the 90-day forecasting period (1st June to 29th August 2020), the model predicts 48,501 (46,170-50,989) cumulative deaths in England (**Supplementary Table 4**). By comparison, the ONS reported 46,539 cumulative deaths up to 12th June in England (registered up to 27th June).

In the scenarios where R_t increases on the 1st of June and then remains constant, for increases from the median 0.75 to 0.80, 0.85, 0.90, 0.95 and 1, the model predicts median excess deaths of 257 (95% CI 108-492), 632 (95% CI 265-1,208), 1,173 (95% 493-2,240), 1,971(95% 828-3,764) and 3,174 (95% CI 1,334-6,060) respectively. Increases of R_t to 1.05 and 1.1, with resultant exponential growth, lead to excess median deaths of 5017 (95% CI 2,109-9,578), and 7,878 (3,313-15,037) respectively (**Figure 3** and **Supplementary Table 4**).

In scenarios where R_t increases on the 1st June, 15th June and 4th July, we find that compared to the baseline scenario, modest increases of R_t to 0.80, 0.85, 0.90, on these dates respectively would lead to 508 (95% CI 213-972) excess deaths. If R_t increases to 0.90, 0.95 and 1 at these time points, then excess estimated deaths increase to 1,848 (95% CI 776-3,534). In these scenarios R_t remains ≤1 (**Figures 3-5** and **Supplementary Table 4**). Increases of R_t above 1 at any point of results in rapid increases in cases, and deaths, predicting a second wave of the epidemic within England (**Figure 4-5** and **Supplementary Table 4**).

Even in a conservative scenario where R_t increases from 0.75 to 0.80 on the 1st of June and then remains constant thereafter, the model predicts an excess of 26,447 (95% CI 11,105-50,549) cumulative cases over 90 days. On the other hand, the scenario with the largest

For peer review only - http://bmjopen.bmj.com/site/about/guidelines.xhtml

changes in R_t, but still remaining ≤1, predicts an excess of up to 421,310 (95% CI 177,012-804,811) (**Figures 6-8** and **Supplementary Table 4**).

Forecasts from an elimination scenario

Compared to the baseline scenario of R_t staying at 0.75, we find that maintaining R_t at 0.60 and 0.70 would result in 44,302(95% CI 84684-18600) and 19,968 (95% CI 38168-8384) fewer cumulative cases, and 462 (95% CI 194-884) and 204 (95% CI 389-86) fewer deaths over the modelled 90-day period, respectively **(Figure 3, Figure 6, Supplementary Table 4**).

Robustness of model in sensitivity analyses

Using uninformative (no prior specified) priors for R_t did not materially alter the median estimates of R_t, although uncertainty around estimates was predictably increased (**Supplementary Figure 3**). This suggests our estimates are robust to the priors specified.

Using a longer SI leads to an increase in the estimated R_0, although subsequent estimates following easing of lockdown remain broadly similar (**Supplementary Figure 4**). This model is comparable to the primary model with regard to fit to observed deaths (**Supplementary Figure 5**) and in predicted excess deaths and cases in scenarios where R_t increases (**Supplementary Table 5**).

Discussion

In this paper we describe a Bayesian model for inferring incident cases and reproduction numbers from daily death data, and for forecasting the impact of future changes in *R*. Our findings provide important quantification of the likely impact of relaxing lockdown measures in England, and to our knowledge, this is the first study to comprehensively assess this through several plausible scenarios. We show that even in scenarios in which *R* remains ≤1 (in line with the UK government's stated aim), small increases in R_t from lifting lockdown measures, can lead to a substantial excess of deaths with 3,174 (95% CI 1,334-6,060) in the most severe scenario modelled.

Our model inferences are robust to modelling assumptions of serial interval distribution, and specified priors. Our estimated R_t of 0.75 following 13th May is consistent with estimates from the SAGE group advising government.[11] We have assessed increases in R_t that are entirely plausible, given the data from other European countries that have started easing lockdown.[3] Our model predicts a substantial excess of cases and deaths in scenarios where *R* remains ≤1. Rises in R_t above 1 would lead to exponential increases in cases, and subsequently deaths. In contrast, we show that pursuing an elimination strategy where R_t would be suppressed to 0.6 or 0.7 could prevent a median estimated 462 and 204 deaths, and 44,302, and 19,968 cases, respectively.

Unlike other European countries, the UK began to ease lockdown when community transmission was still high with an estimated incidence of infection of >8000 cases and >300 deaths being observed per day in England. In Denmark and Germany some of the increases in *R* since easing lockdown, have likely been mitigated by the low levels of transmission at the point of easing lockdown. Another important factor may be the use of aggressive case detection and contact tracing approaches, which the UK seems unlikely to have fully

For peer review only - http://bmjopen.bmj.com/site/about/guidelines.xhtml

operational till later this year, and the existing system is at risk of being overwhelmed by major increases in incident cases. Given the lack of comprehensive testing, the UK's current estimates of R_t rely on incident deaths (as used by the MRC Nowcasting and Forecasting model)[10], which means that changes in R_t reflect changes in community transmission from a median of 2-3 weeks ago.[11] With lockdown being eased in 2-weekly steps, this means that by the time we detected the impact of one step, the next one would already have been instituted so mitigating these impacts would be extremely challenging. The UK SAGE has also expressed concerns that increases in R up to 1.2 may continue undetected for longer periods of time.[14] These concerns have been borne out by the recent surge in cases observed in Leicester,[4] where the increase in case numbers were only detected two weeks after the event. Our findings strongly suggest that despite small increases in R, we would likely see substantial increases in cases and deaths, which may be detected too late to mitigate impact of the lockdown easing measures that led to these. This is particularly important when we consider the impact on health services, which have managed to deal with the pandemic by suspending much of routine healthcare, which is likely to substantially increase indirect causes of deaths from cancer, and cardiovascular disease. This also has important implications for population health, as we observe multi-system long-term sequelae among those infected with COVID-19.

We acknowledge some important limitations of our model. The first is that it is based on a back calculation of cases based on incident deaths, which are likely to underestimated due to reporting delays and underreporting. Second, our model is reliant on inferring cases, and reproduction numbers, which depend on the assumed distributions of the serial interval, and the time of onset to death distributions. While we have based our assumptions on the literature, misspecification of these would influence our estimates. While we have evaluated this, greater deviations from true estimates would make our forecasting less reliable. Third, similar to Flaxman et al, our model uses the IFR as a multiplier for the distribution of time from infection to death, in the absence of reliable population level case fatality rates (CFR). While this would not affect the estimation of deaths, if the CFR were higher (due to large proportions of cases being asymptomatic), then the predicted case numbers would be overestimated by our model. We note, however that the estimate of IFR we used (1.1%) is consistent with the CFR estimated in previously from Beijing.[15] We have also, for simplicity, assumed that IFR remains constant throughout the pandemic and the forecasting period, and this may not reflect complex heterogeneity in IFR over time. Finally, we do not consider the impact of mitigatory measures in our current modelling. However mitigatory measures are likely to be implemented with significant delays from when community transmission increases, namely when changes in R are detected. If such measures, like re-introducing lockdown, or school closures, were re-implemented, they may reduce the impact of the modelled scenarios.

In summary, we show that increases in R_t as a result of easing lockdown would have a substantial impact on incident transmission and deaths for even modest increases that still maintain $R_t \leq 1$. We argue for a more cautious approach with a focus on elimination, by reducing R_t and incident cases to low levels prior to easing lockdown measures and then too with careful monitoring.

For peer review only - http://bmjopen.bmj.com/site/about/guidelines.xhtml

Authors' contributions:
DG conceived the study and designed the model with NS. DG programmed the model and made the figures. HZ and NS consulted on the model design. All authors interpreted the results, contributed to writing the Article, and approved the final version for submission.

Declaration of interests:
None.

Data sharing:
All data on daily deaths used in this study were taken from the Office of National Statistics website (https://www.ons.gov.uk/peoplepopulationandcommunity/birthsdeathsandmarriages/deaths/datasets/weeklyprovisionalfiguresondeathsregisteredinenglandandwales).

The code for the model, and dataset analysed is available at:
https://github.com/dgurdasani1/lockdownsim

Ethics
No ethical approval was obtained for this study, as only publicly available aggregate data on incident deaths was analysed.

Acknowledgements and Funding:
HZ is partly funded by the Bernard Wolfe Health Neuroscience Fund at the University of Cambridge. NS is funded by a Strategic Award from the Wellcome Trust. DG is funded by the UKRI/Rutherford HDR-UK fellowship programme (reference MR/S003711/2).
We would like to particularly acknowledge and thank Flaxman et al and the team at Imperial College London for making their code available for us to use.

References:

1. Coronavirus: How lockdown is being lifted across Europe. BBC. 2020.
2. Gallagher J. Coronavirus: Risk in UK lockdown easing too soon, warn scientists. BBC. 2020.
3. Robert Koch Institute G. Coronavirus Disease 2019 (COVID-19), Daily Situation Report of the Robert Koch Institute 2020.
4. Leicester lockdown tightened as coronavirus cases rise. BBC. 2020.
5. Flaxman S MS, Gandy, A, Unwin HJT, Coupland H, Mellan, TA, Zhu H, Berah T, Eaton JW et al. Estimating the effects of non-pharmaceutical interventions on COVID-19 in Europe. Nature; 2020. https://doi.org/10.1038/s41586-020-2405-7
6. Deaths registered weekly in England and Wales, provisional. In: Statistics OoN, editor.; 2020.
7. Nishiura H, Linton NM, Akhmetzhanov AR. Serial interval of novel coronavirus (COVID-19) infections. *Int J Infect Dis* 2020; **93**: 284-6.
8. Vehtari A, Gelman, A., and Gabry, J. Practical Bayesian model evaluation using leave-one-out cross-validation and WAIC. Statistics and Computing. *Statistics and Computing* 2017; **27**(5): 1413-32.

For peer review only - http://bmjopen.bmj.com/site/about/guidelines.xhtml

9. Dehning J, Zierenberg J, Spitzner FP, et al. Inferring change points in the spread of COVID-19 reveals the effectiveness of interventions. *Science* 2020.
10. Joint PHE Modelling Cell MBUC-WG. COVID-19: nowcast and forecast, 2020.
11. Gov.uk. Government publishes coronavirus (COVID-19) R value and growth rates. 2020.
12. Chan Y-W D FS, Lam T-L T, Leung M-H J, Wong M-L, Lam, H-Y, Chuang S-K. Transmission dynamics, serial interval and epidemiology of COVID-19 diseases in Hong Kong under different control measures. Wellcome Open Research; 2020.
13. STATUS FOR SMITTETRYKKET I DANMARK, 29/4 2020: Statens Serum Institut.
14. SAGE. Thirty-eighth SAGE meeting on COVID-19, 21st May 2020, 2020.
15. Leung K, Wu JT, Liu D, Leung GM. First-wave COVID-19 transmissibility and severity in China outside Hubei after control measures, and second-wave scenario planning: a modelling impact assessment. *Lancet* 2020; **395**(10233): 1382-93.

Figure Legends

Figure 1: Estimated time-varying reproduction number (R_t) for England
The figure shows the R_t estimated by Model 3 (blue) with 95% credible intervals (grey) with a serial interval of mean 4.7 and SD 2.9 days. From 3.65 (CI 3.36-3.96), R_t drops on the 16th March and 23rd March (indicated by vertical dashed lines) when social distancing and lockdown were instituted, reaching a low of 0.66 (95% CI 0.34-1.04) in the week of the 30th March. The last estimated R_t is 0.75 (95% CI 0.50-1.00) following the 13th May.

Figure 2: Model fit to observed death data
Daily deaths predicted by Model 3 (blue) with 95% credible intervals (grey) show a good fit to the observed deaths from the ONS (red)

Figure 3. Predicted deaths with R_t increasing on 1st June
(A) The model compared scenarios in which R_t increases to 0.80 (light green), 0.85 (green), 0.90 (dark blue), 0.95 (red), 1 (purple) and 1.05 (brown) and then remains constant for the 90-day forecasting period. The comparator baseline scenario is of R_t remaining at 0.75 (black) and two elimination strategies of R_t reducing to 0.7 (yellow) and 0.6 (light blue) were also considered. Vertical dashed lines represent time-points of easing lockdown. (B), (C) the incident and cumulative deaths increase in all scenarios in which R_t increases and reduces in the two elimination scenarios.

Figure 4. Predicted deaths in scenarios of R_t increase on 1st and 15th June compared with baseline scenario
(A) The model compared scenarios in which R_t increases to 0.80 (light green), 0.85 (green), 0.90 (blue), 0.95 (red), 1 (purple) and 1.05(brown) and then further by 0.05 on the 15th June and then remaining constant for the 90-day forecasting period. The comparator baseline scenario is of R_t remaining at 0.75 (black). Vertical dashed lines represent time-points of easing lockdown. (B), (C) The incident and cumulative deaths increase in all scenarios in which R_t increases.

Figure 5. Predicted deaths in scenarios of R_t increase on 1st June, 15th June and 4th July compared with baseline scenario

For peer review only - http://bmjopen.bmj.com/site/about/guidelines.xhtml

(A) The model compared scenarios in which R_t increases to 0.80 (light green), 0.85 (green), 0.90 (blue), 0.95 (red), 1 (purple) and 1.05(brown) and then further by 0.05 on the 15th June and then again by 0.05 on the 3rd July before remaining constant for the 90-day forecasting period. The comparator baseline scenario is of R_t remaining at 0.752 (black). Vertical dashed lines represent time-points of easing lockdown. (B), (C) The incident and cumulative deaths increase in all scenarios in which R_t increases.

Figure 6. Predicted cases in scenarios of R_t increase on 1st June compared with baseline and elimination scenarios

(A) The model compared scenarios in which R_t increases to 0.80 (light green), 0.85 (green), 0.90 (dark blue), 0.95 (red), 1 (purple) and 1.05(brown) and then remains constant for the 90-day forecasting period. The comparator baseline scenario is of R_t remaining at 0.752 (black) and two elimination strategies of R_t reducing to 0.7 (yellow) and 0.6(light blue) were also considered. Vertical dashed lines represent time-points of easing lockdown. (B), (C) the incident and cumulative cases increase in all scenarios in which R_t increases and reduces in the two elimination scenarios.

Figure 7. Predicted cases in scenarios of R_t increase on 1st June and 15th June compared with the baseline scenario

(A) The model compared scenarios in which R_t increases to 0.80 (light green), 0.85 (green), 0.90 (blue), 0.95 (red), 1 (purple) and 1.05(brown) and then further by 0.05 on the 15th June and then remaining constant for the 90-day forecasting period. The comparator baseline scenario is of R_t remaining at 0.752 (black). Vertical dashed lines represent time-points of easing lockdown. (B), (C) The incident and cases increase in all scenarios in which R_t increases.

Figure 8. Predicted cases in scenarios of R_t increase on 1st June and 15th June and 4th July compared with the baseline scenario

(A) The model compared scenarios in which R_t increases to 0.80 (light green), 0.85 (green), 0.90 (blue), 0.95 (red), 1 (purple) and 1.05(brown) and then further by 0.05 on the 15th June and then again by 0.05 on the 3rd July before remaining constant for the 90-day forecasting period. The comparator baseline scenario is of R_t remaining at 0.752 (black). Vertical dashed lines represent time-points of easing lockdown. (B), (C) The incident and cumulative cases increase in all scenarios in which R_t increases.

For peer review only

Figure 1: Estimated time-varying reproduction number (R_t) for England
The figure shows the R_t estimated by Model 3 (blue) with 95% credible intervals (grey) with a serial interval of mean 4.7 and SD 2.9 days. From 3.65 (CI 3.36-3.96), R_t drops on the 16th March and 23rd March (indicated by vertical dashed lines) when social distancing and lockdown were instituted, reaching a low of 0.66 (95% CI 0.34-1.04) in the week of the 30th March. The last estimated R_t is 0.75 (95% CI 0.50-1.00) following the 13th May.

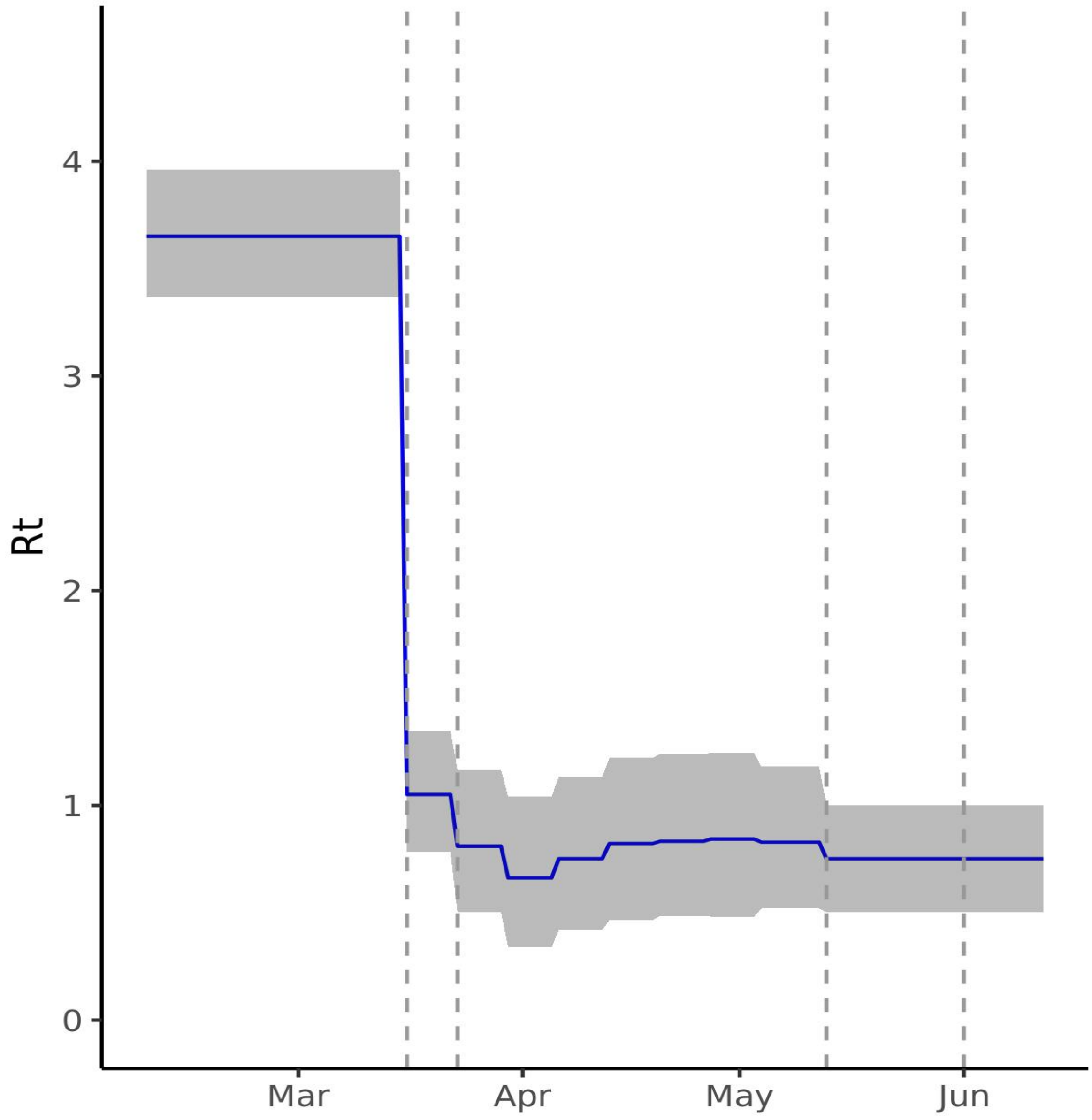

Figure 2: Model fit to observed death data
Daily deaths predicted by Model 3 (blue) with 95% credible intervals (grey) show a good fit to the observed deaths from the ONS (red)

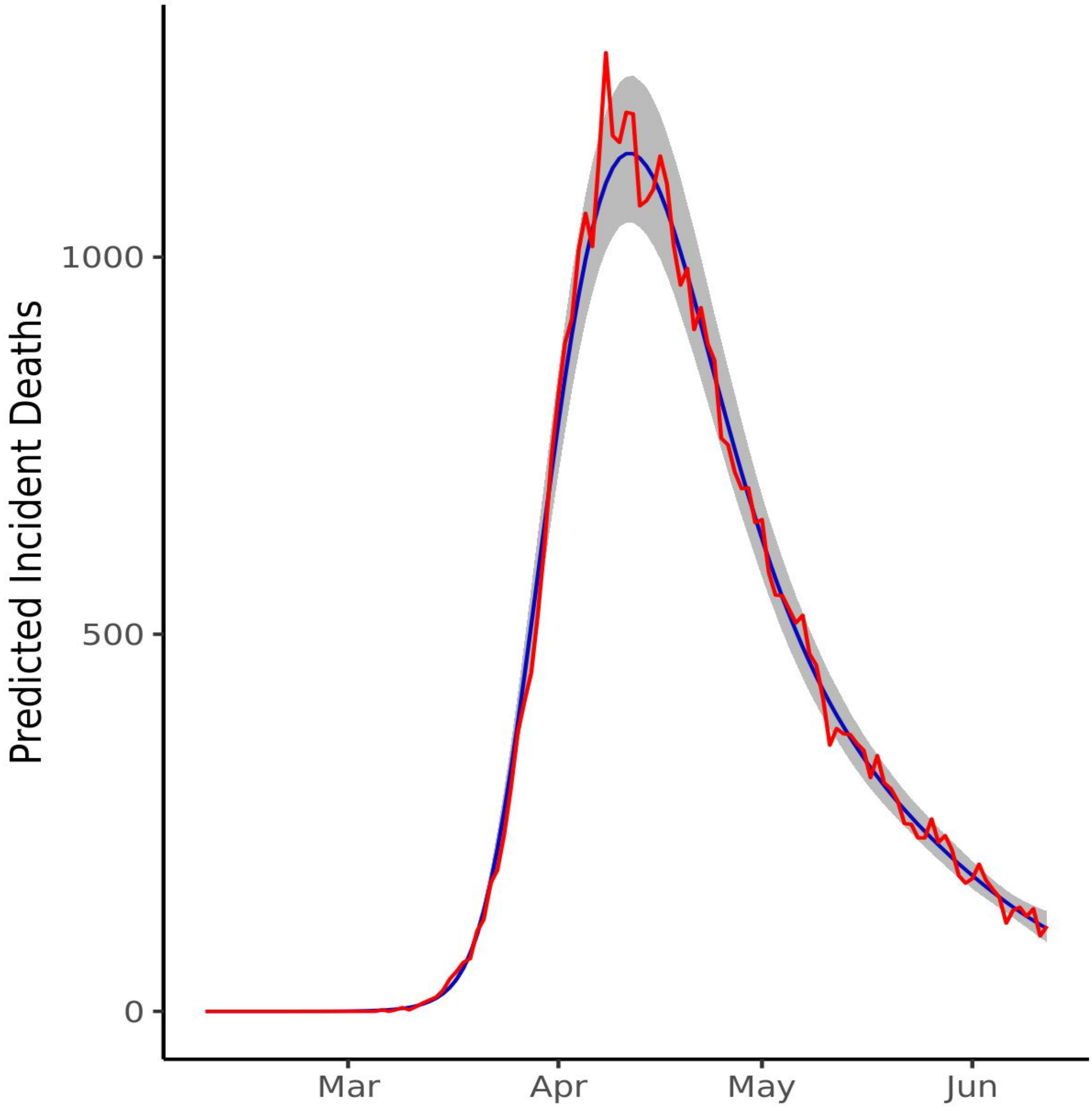

Figure 3. Predicted deaths with R_t increasing on 1st June

(A) The model compared scenarios in which R_t increases to 0.80 (light green), 0.85 (green), 0.90 (dark blue), 0.95 (red), 1 (purple) and 1.05 (brown) and then remains constant for the 90-day forecasting period. The comparator baseline scenario is of R_t remaining at 0.75 (black) and two elimination strategies of R_t reducing to 0.7 (yellow) and 0.6 (light blue) were also considered. Vertical dashed lines represent time-points of easing lockdown. (B), (C) the incident and cumulative deaths increase in all scenarios in which R_t increases and reduces in the two elimination scenarios.

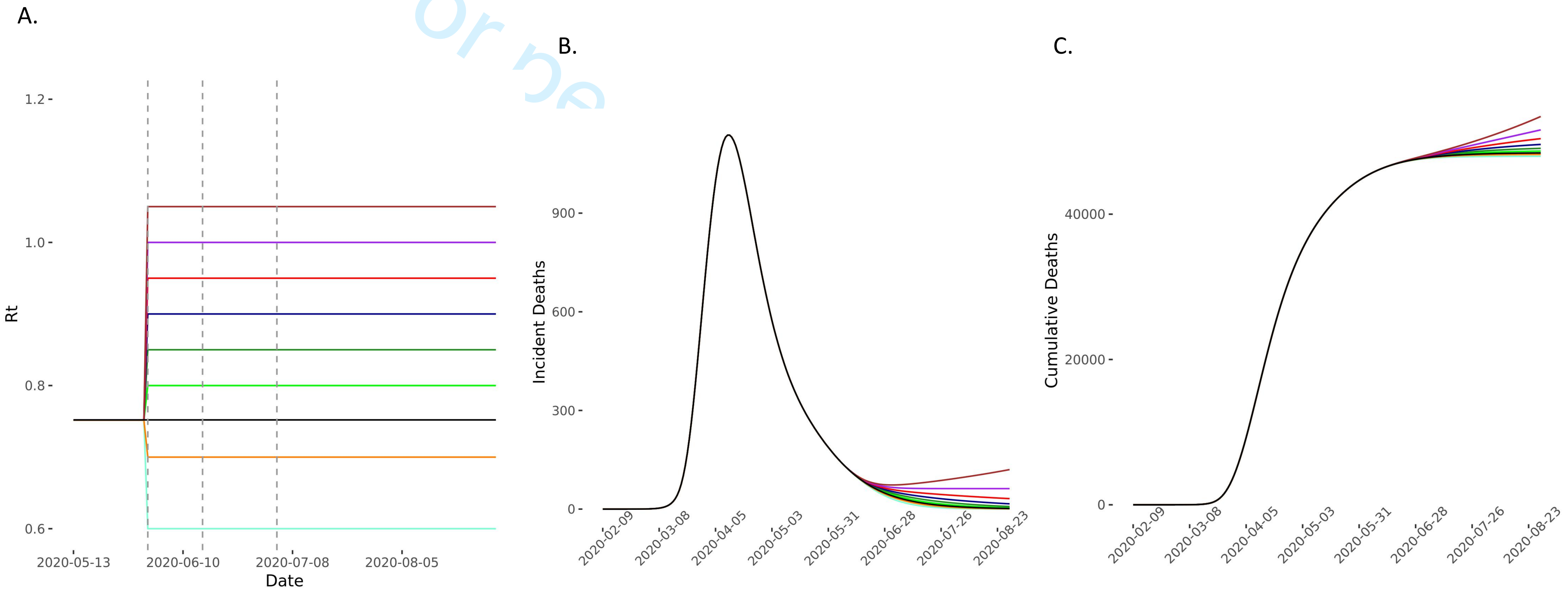

Figure 4. Predicted deaths in scenarios of R_t increase on 1st and 15th June compared with baseline scenario

(A) The model compared scenarios in which R_t increases to 0.80 (light green), 0.85 (green), 0.90 (blue), 0.95 (red), 1 (purple) and 1.05(brown) and then further by 0.05 on the 15th June and then remaining constant for the 90-day forecasting period. The comparator baseline scenario is of R_t remaining at 0.75 (black). Vertical dashed lines represent time-points of easing lockdown. (B), (C) The incident and cumulative deaths increase in all scenarios in which R_t increases.

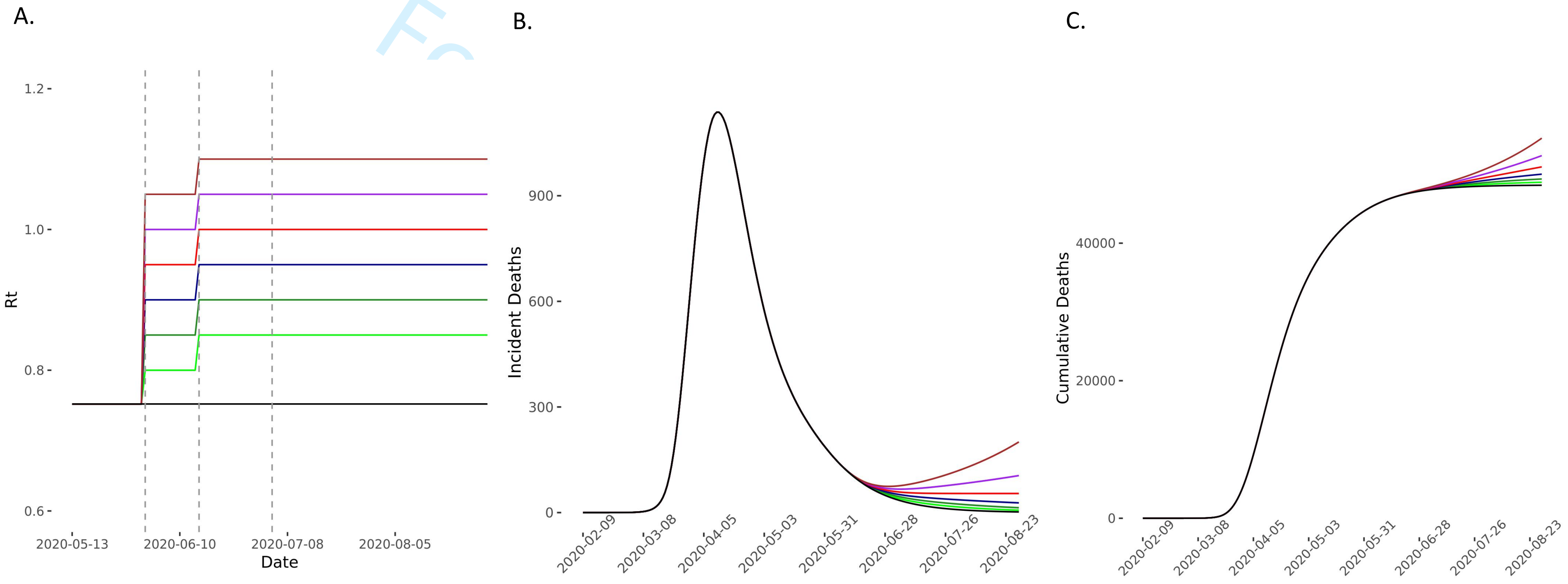

Figure 5. Predicted deaths in scenarios of R_t increase on 1st June, 15th June and 4th July compared with baseline scenario

(A) The model compared scenarios in which R_t increases to 0.80 (light green), 0.85 (green), 0.90 (blue), 0.95 (red), 1 (purple) and 1.05(brown) and then further by 0.05 on the 15th June and then again by 0.05 on the 3rd July before remaining constant for the 90-day forecasting period. The comparator baseline scenario is of R_t remaining at 0.752 (black). Vertical dashed lines represent time-points of easing lockdown. (B), (C) The incident and cumulative deaths increase in all scenarios in which R_t increases.

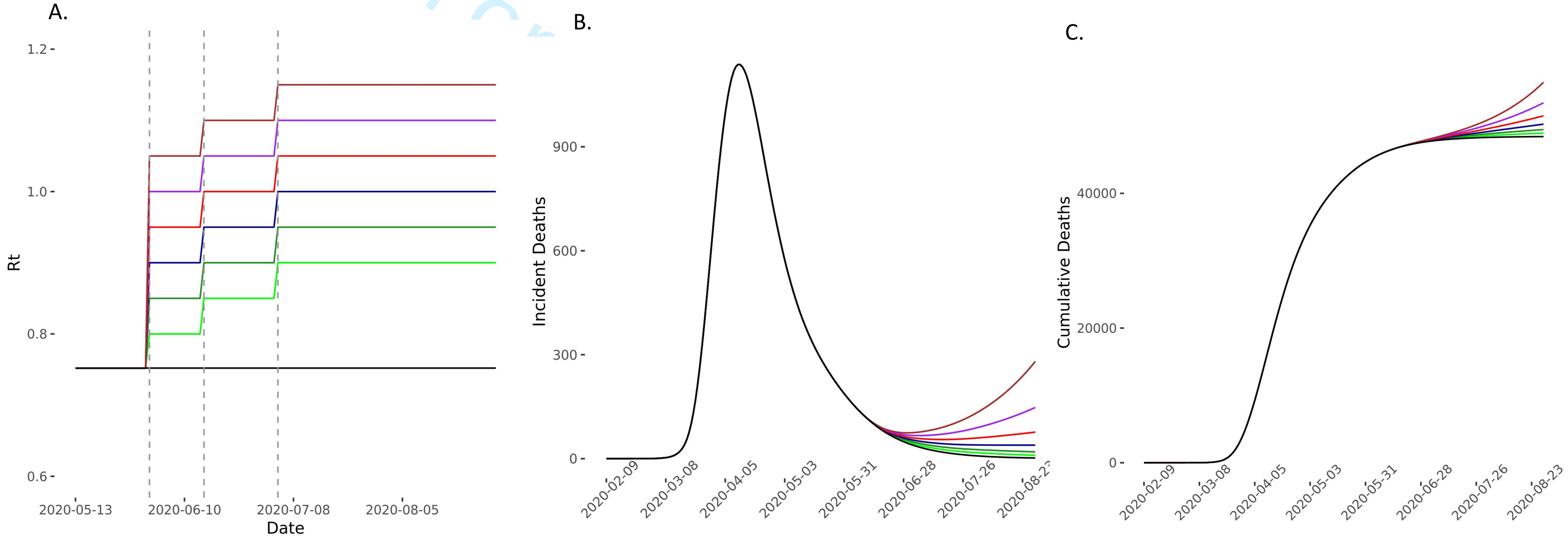

Figure 6. Predicted cases in scenarios of R_t increase on 1st June compared with baseline and elimination scenarios
(A) The model compared scenarios in which R_t increases to 0.80 (light green), 0.85 (green), 0.90 (dark blue), 0.95 (red), 1 (purple) and 1.05(brown) and then remains constant for the 90-day forecasting period. The comparator baseline scenario is of R_t remaining at 0.752 (black) and two elimination strategies of R_t reducing to 0.7 (yellow) and 0.6(light blue) were also considered. Vertical dashed lines represent time-points of easing lockdown. (B), (C) the incident and cumulative cases increase in all scenarios in which R_t increases and reduces in the two elimination scenarios.

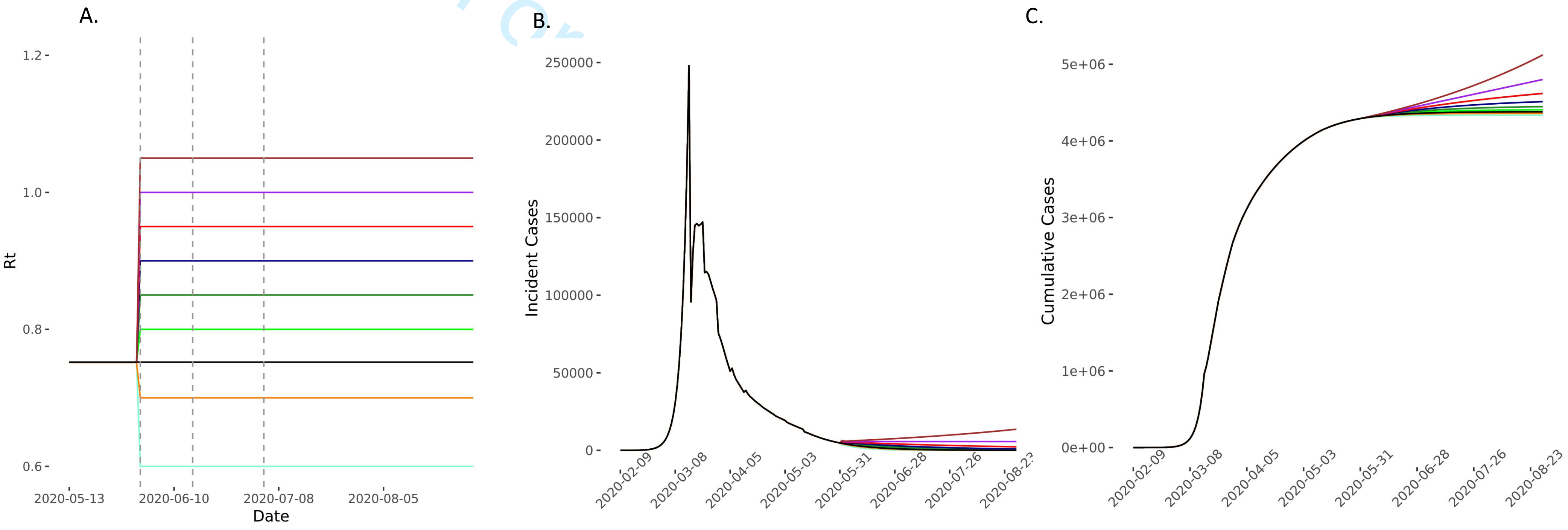

Figure 7. Predicted cases in scenarios of R_t increase on 1st June and 15th June compared with the baseline scenario

(A) The model compared scenarios in which R_t increases to 0.80 (light green), 0.85 (green), 0.90 (blue), 0.95 (red), 1 (purple) and 1.05(brown) and then further by 0.05 on the 15th June and then remaining constant for the 90-day forecasting period. The comparator baseline scenario is of R_t remaining at 0.752 (black). Vertical dashed lines represent time-points of easing lockdown. (B), (C) The incident and cases increase in all scenarios in which R_t increases.

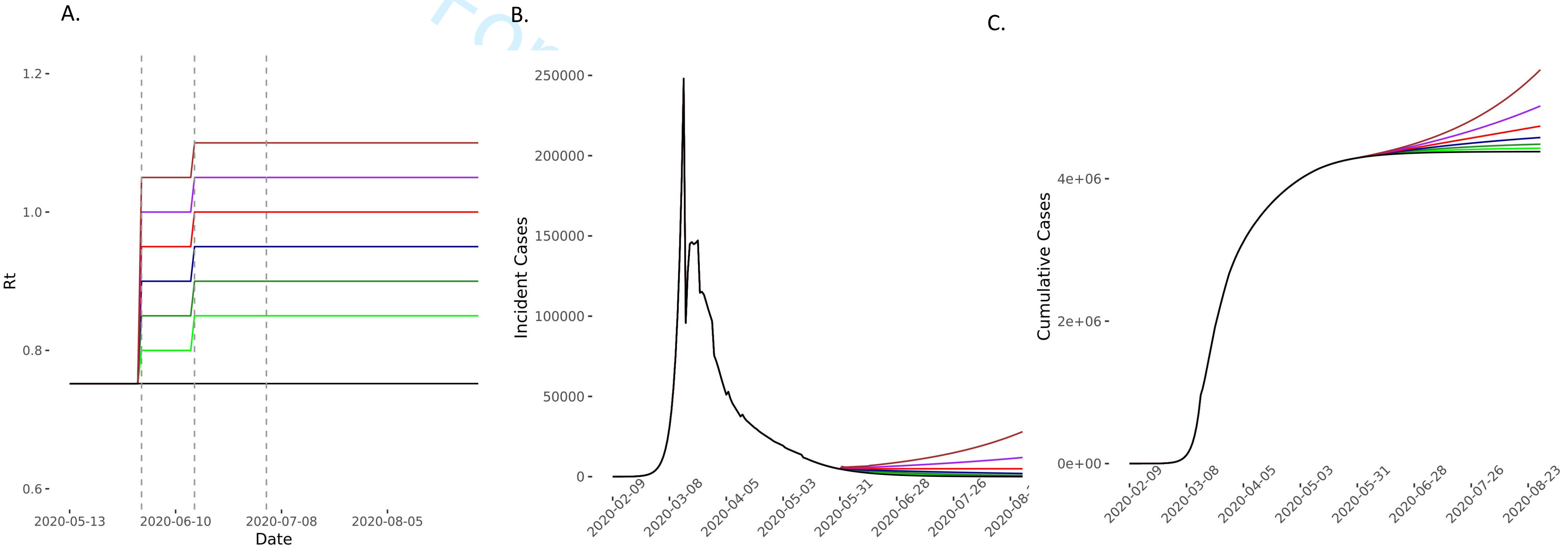

Figure 8. Predicted cases in scenarios of R_t increase on 1st June and 15th June and 4th July compared with the baseline scenario

(A) The model compared scenarios in which R_t increases to 0.80 (light green), 0.85 (green), 0.90 (blue), 0.95 (red), 1 (purple) and 1.05(brown) and then further by 0.05 on the 15th June and then again by 0.05 on the 3rd July before remaining constant for the 90-day forecasting period. The comparator baseline scenario is of R_t remaining at 0.752 (black). Vertical dashed lines represent time-points of easing lockdown. (B), (C) The incident and cumulative cases increase in all scenarios in which R_t increases.

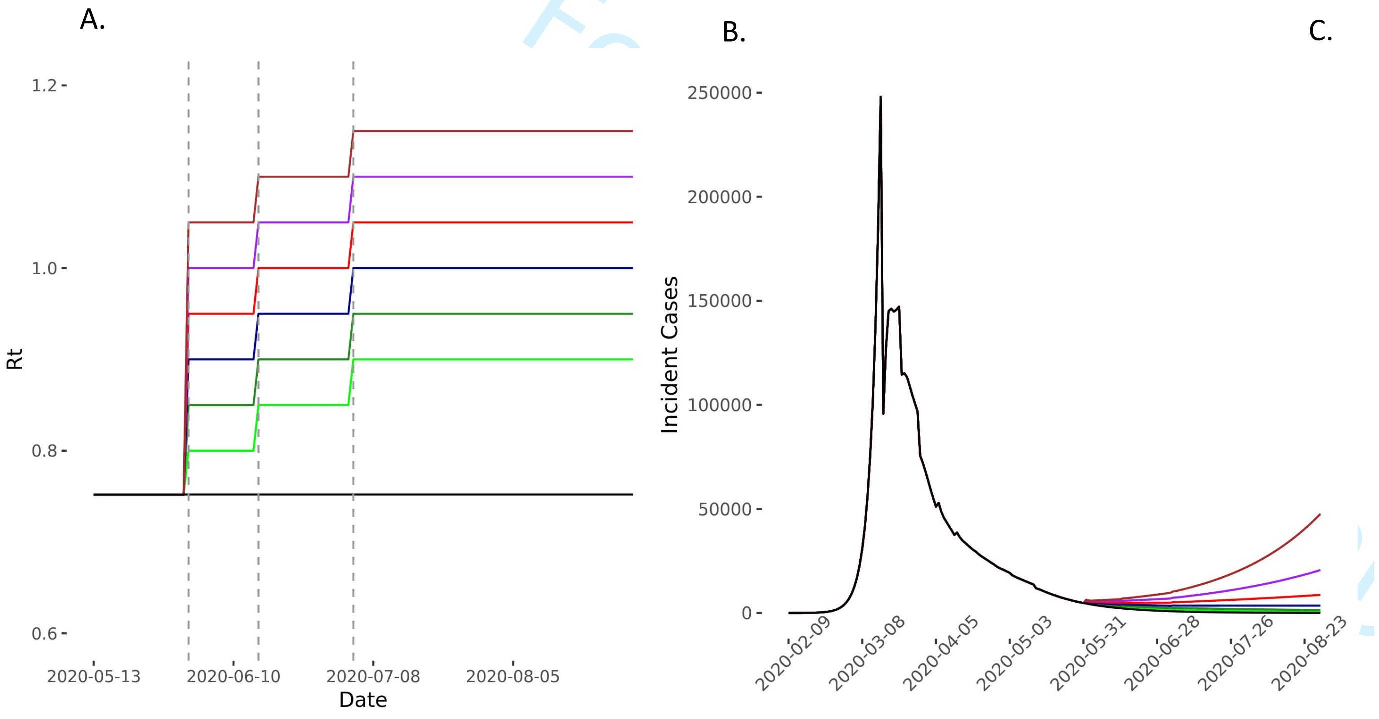

SUPPLEMENTARY MATERIALS

Modelling the impact of lockdown easing measures on cumulative COVID-19 cases and deaths in England

Ziauddeen H, PhD [1,2,3], Subramaniam N, BSc [1,2], Gurdasani D, PhD† [4].

Supplementary Table 1: Parameters estimated by Bayesian model

Variable	Parameter	No.	Priors
c_t, where t=1…6	Number of initial cases on first six days	6	exponential(1.0/tau)
R_0	Baseline Reproduction Number	1	Normal(2.4,0.5)
R_t	Time varying effective reproduction number	9	Normal(0.8,0.25)
φ	variance parameter for negative binomial distribution of deaths	1	normal(0,5)
τ	parameter in prior of c_t	1	exponential(0.03)

Supplementary Table 2: Comparison of Bayesian models with different constraints on changes in R_t

Model	RMSE	EPLD	SE	diff_EPLD_model3	diff_SE_model3
model 1	38.0	-505.1	25.0	-1.5	0.5
model 2	28.3	-504.8	24.5	-1.2	0.9
model 3	28.0	-503.6	24.8	NA	NA

Supp. Table 2 represents model comparisons between Models 1-3, as specified in the text. RMSE represents the Root mean squared error between estimated and observed deaths for each model. EPLD represents the expected log pointwise predictive density which approximates leave-one-out (LOO) cross-validation. Less negative scores suggest better fit. SE is the standard error of EPLD. We assess the difference in EPLD between all models and the best performing model (Model 3 in this case), comparing the difference in EPLD (diff_EPLD_model3) with the standard error of the difference (diff_SE_model3). Although all three models appear comparable in performance, Model 3 appears to show the best fit with the lowest RMSE, and the least negative EPLD.

For peer review only

Supplementary Table 3: Comparison of models excluding specific change points for R_t

change points removed	RMSE	EPLD	SE	diff_EPLD	diff_SE
16th March	118.1	-576.0	26.1	-70.9	5.3
23rd March	44.6	-506.7	25.2	-1.6	1.0
13th May	40.2	-504.8	25.0	0.3	0.4
1st June	38.1	-505.2	25.0	-0.1	0.0
None (all included)	38.0	-505.1	25.0	NA	NA

Supp. Table 3 represents model comparisons between Models that constrain Rt at each of the 4 hypothesised change points at which point social distancing or lockdown measures were introduced (16th March and 23rd March), or when lockdown measures were eased (13th May and 1st June). The first column represents the change point left out in each model, with the last model with all three change points being the comparator, as specified in the text. RMSE represents the Root mean squared error between estimated and observed deaths for each model. EPLD represents the expected log pointwise predictive density which approximates leave-one-out (LOO) cross-validation. Less negative scores suggest better fit. SE is the standard error of EPLD. We assess the difference in EPLD between all models and the model with all three change points, comparing the difference in EPLD (diff_EPLD) with the standard error of the difference (diff_SE). The model leaving out 16th March as a change point, i.e. constraining Rt to remain constant at this point appears to adversely impact fit the most.

For peer review only

Supplementary Table 4: Cumulative cases and deaths in lockdown easing scenarios in primary model

Rt 1st June	Rt 15th June	Rt 4th July	Cumulative cases	Cumulative deaths	Cases difference from baseline	Death difference from baseline
0.752	0.752	0.752	4411594(4199223-4639250)	48501(46170-50989)	0(0,0)	0(0,0)
0.6	0.6	0.6	4364386(4162299-4580834)	48006(45783-50386)	-44302(-84684--18600	-462(-884--194)
0.65	0.65	0.65	4374559(4170697-4593499)	48115(45875-50523)	-33831(-64668--14204)	-350(-669--147)
0.7	0.7	0.7	4391027(4183302-4610584)	48286(46007-50696)	-19968(-38168--8384)	-204(-389--86)
0.75	0.75	0.75	4410590(4198499-4637531)	48494(46163-50977)	-908(-1736--381)	-9(-17--4)
0.75	0.75	0.8	4415149(4201945-4645342)	48518(46186-51016)	3069(1285-5890)	19(8-37)
0.75	0.8	0.8	4424153(4209052-4658126)	48612(46255-51149)	11497(4814-22058)	102(43-195)
0.75	0.8	0.85	4430866(4213721-4668154)	48654(46293-51225)	18197(7620-34906)	145(61-278)
0.8	0.8	0.8	4439684(4219884-4679358)	48771(46375-51380)	26447(11105-50549)	257(108-492)
0.8	0.8	0.85	4447876(4225283-4692920)	48827(46413-51458)	34303(14397-65598)	308(129-589)
0.8	0.85	0.85	4461240(4232489-4716984)	48954(46492-51680)	47523(19934-90933)	431(181-825)
0.8	0.85	0.9	4474630(4243279-4736698)	49036(46538-51811)	60851(25519-116475)	508(213-972)
0.85	0.85	0.85	4481739(4247839-4745973)	49166(46614-52010)	67576(28376-129149)	632(265-1208)
0.85	0.85	0.9	4498639(4257710-4770487)	49246(46692-52138)	83109(34888-158891)	722(303-1379)
0.85	0.9	0.9	4521199(4273493-4806484)	49446(46808-52428)	104334(43782-199536)	907(381-1733)
0.85	0.9	0.95	4547931(4291851-4848863)	49592(46917-52640)	130823(54887-250256)	1043(438-1994)
0.9	0.9	0.9	4549190(4292969-4850945)	49730(47007-52865)	132381(55595-252977)	1173(493-2240)
0.9	0.9	0.95	4579138(4311424-4905124)	49887(47120-53118)	163082(68471-311726)	1330(559-2542)
0.9	0.95	0.95	4613802(4328984-4965451)	50162(47308-53602)	197988(83107-378532)	1610(676-3077)
0.9	0.95	1	4667450(4358502-5053567)	50397(47439-54007)	250499(105129-479023)	1848(776-3534)
0.95	0.95	0.95	4655308(4352263-5032669)	50517(47499-54226)	239051(100411-456749)	1971(828-3764)
0.95	0.95	1	4718132(4385399-5139609)	50790(47648-54680)	299596(125815-572543)	2246(943-4290)
0.95	1	1	4779022(4412370-5246023)	51226(47883-55381)	358354(150465-684935)	2672(1122-5106)
0.95	1	1.05	4880364(4464544-5437916)	51650(48103-56116)	462002(193954-883178)	3087(1296-5899)
1	1	1	4840595(4444280-5359379)	51743(48160-56290)	421310(177012-804811)	3174(1334-6060)
1	1	1.05	4959206(4498273-5586656)	52235(48368-57131)	540234(226938-1032145)	3649(1533-6970)
1	1.05	1.05	5055758(4548961-5767421)	52892(48712-58297)	641220(269327-1225202)	4303(1808-8220)
1	1.05	1.1	5264669(4632979-6143068)	53598(49059-59632)	844596(354706-1613979)	5018(2108-9587)
1.05	1.05	1.05	5156984(4594880-5946919)	53594(49059-59623)	741957(311832-1416940)	5017(2109-9578)
1.05	1.05	1.1	5397044(4692140-6391841)	54411(49421-61165)	974252(409410-1860772)	5833(2452-11138)
1.05	1.1	1.1	5574165(4770085-6732948)	55401(49905-63038)	1150799(483559-2198106)	6843(2876-13067)
1.05	1.1	1.15	5969381(4946974-7481001)	56609(50491-65190)	1546934(649955-2954983)	8065(3390-15402)
1.1	1.1	1.1	5744209(4843263-7044331)	56428(50400-64839)	1317940(554129-2516086)	7878(3313-15037)
1.1	1.1	1.15	6189495(5035202-7885232)	57860(50954-67461)	1768512(743504-3376542)	9269(3898-17692)
1.1	1.15	1.15	6501607(5163364-8475727)	59458(51650-70470)	2081127(874883-3973567)	10834(4556-20682)
1.1	1.15	1.2	7272289(5484637-9955130)	61543(52551-74465)	2846203(1196439-5434639)	12908(5427-24642)

Supplementary Table 4 represents the estimated cumulative deaths, cumulative cases, and excess deaths and cases in different scenarios of changing Rt at points of easing lockdown in comparison with the baseline scenario of Rt remaining constant at 0.752.

For peer review only

Supplementary Table 5: Cumulative cases and deaths in lockdown easing scenarios in model with long serial interval

Rt 1st June	Rt 15th June	Rt 4th July	Cumulative cases	Cumulative deaths	Cases difference from baseline	Death difference from baseline
0.691	0.691	0.691	4404236(4183512-4622330)	48411(45990-50805)	0(0-0)	0(0-0)
0.6	0.6	0.6	4371866(4158398-4583033)	48078(45733-50400)	-32402(-52309--17783)	-330(-533--181)
0.65	0.65	0.65	4387428(4171118-4603369)	48241(45863-50609)	-16515(-26661--9065)	-166(-268--91)
0.7	0.7	0.7	4408635(4186726-4627236)	48451(46022-50849)	4158(2283-6712)	41(23-66)
0.7	0.7	0.75	4413466(4190714-4632818)	48487(46047-50893)	8904(4865-14430)	75(41-121)
0.7	0.75	0.75	4421929(4197326-4644215)	48571(46126-50997)	17612(9599-28584)	160(87-259)
0.7	0.75	0.8	4428891(4202474-4652651)	48618(46159-51054)	24664(13432-40046)	206(112-334)
0.75	0.75	0.75	4436254(4207602-4661170)	48717(46226-51175)	31769(17441-51275)	307(168-494)
0.75	0.75	0.8	4444061(4214414-4673025)	48768(46266-51240)	39710(21767-64168)	358(197-579)
0.75	0.8	0.8	4456057(4225564-4689908)	48884(46358-51403)	51923(28426-84007)	473(259-765)
0.75	0.8	0.85	4468509(4233843-4705662)	48955(46423-51508)	63758(34875-103225)	544(298-881)
0.8	0.8	0.8	4474042(4238163-4713852)	49060(46515-51637)	69645(38242-112391)	652(358-1051)
0.8	0.8	0.85	4487239(4247137-4731986)	49147(46570-51756)	82925(45486-133921)	732(402-1180)
0.8	0.85	0.85	4504368(4260726-4758965)	49297(46674-51983)	100288(54965-162081)	887(487-1433)
0.8	0.85	0.9	4524968(4276399-4786847)	49405(46756-52128)	120111(65789-194244)	997(546-1610)
0.85	0.85	0.85	4528097(4278796-4789875)	49515(46843-52296)	122997(67554-198451)	1107(609-1785)
0.85	0.85	0.9	4550519(4295317-4824987)	49639(46934-52475)	145167(79670-234360)	1229(675-1983)
0.85	0.9	0.9	4575600(4314157-4857930)	49864(47104-52757)	170201(93348-274900)	1441(791-2326)
0.85	0.9	0.95	4608999(4336767-4903946)	50032(47229-53038)	203356(111471-328606)	1608(883-2597)
0.9	0.9	0.9	4605943(4334310-4898052)	50138(47312-53190)	200055(109912-322694)	1716(944-2766)
0.9	0.9	0.95	4643792(4360620-4952873)	50326(47454-53431)	237006(130136-382485)	1902(1046-3067)
0.9	0.95	0.95	4680570(4385671-5007504)	50621(47645-53806)	273569(150145-441642)	2192(1204-3535)
0.9	0.95	1	4735012(4422134-5091796)	50870(47843-54169)	328896(180416-531139)	2446(1343-3946)
0.95	0.95	0.95	4720210(4412771-5069359)	50965(47911-54323)	313876(172518-506126)	2540(1397-4093)
0.95	0.95	1	4781115(4451857-5160478)	51266(48080-54750)	375333(206196-605461)	2822(1552-4549)
0.95	1	1	4836432(4486836-5241471)	51661(48368-55340)	429433(235844-692907)	3219(1770-5191)
0.95	1	1.05	4928929(4549055-5385960)	52032(48613-55941)	521422(286255-841597)	3603(1980-5811)
1	1	1	4892268(4526735-5326989)	52097(48655-56020)	485265(266854-782184)	3667(2018-5906)
1	1	1.05	4995150(4589022-5491882)	52523(48926-56643)	587186(322775-946757)	4092(2252-6593)
1	1.05	1.05	5076092(4640904-5614490)	53078(49274-57480)	668188(367218-1077551)	4639(2552-7476)
1	1.05	1.1	5227156(4734604-5849954)	53653(49678-58359)	820638(450864-1323717)	5217(2869-8409)
1.05	1.05	1.05	5154698(4689409-5733251)	53657(49684-58359)	747364(411252-1204170)	5221(2876-8408)
1.05	1.05	1.1	5321327(4792479-5997797)	54309(50099-59370)	915842(503787-1475921)	5860(3227-9437)
1.05	1.1	1.1	5444111(4858418-6188703)	55069(50551-60585)	1038372(571090-1673570)	6615(3642-10657)
1.05	1.1	1.15	5698707(4995152-6588038)	55936(51099-61889)	1290002(709312-2079494)	7480(4117-12052)
1.1	1.1	1.1	5558967(4921706-6371716)	55831(51060-61736)	1153037(634971-1857036)	7382(4069-11884)
1.1	1.1	1.15	5838364(5075136-6811946)	56779(51632-63173)	1430388(787494-2304058)	8336(4593-13420)
1.1	1.15	1.15	6021434(5176394-7116504)	57812(52276-64824)	1617286(890276-2605292)	9384(5170-15109)
1.1	1.15	1.2	6430941(5404442-7783304)	59109(53001-66839)	2030763(1117659-3271715)	10673(5879-17187)

For peer review only

Supplementary Table 5 represents the estimated cumulative deaths, cumulative cases, and excess deaths and cases in different scenarios of changing Rt at points of easing lockdown in comparison with the baseline scenario of Rt remaining constant at 0.691.

Supplementary Figure 1: Distribution of R-hat for parameters from final model

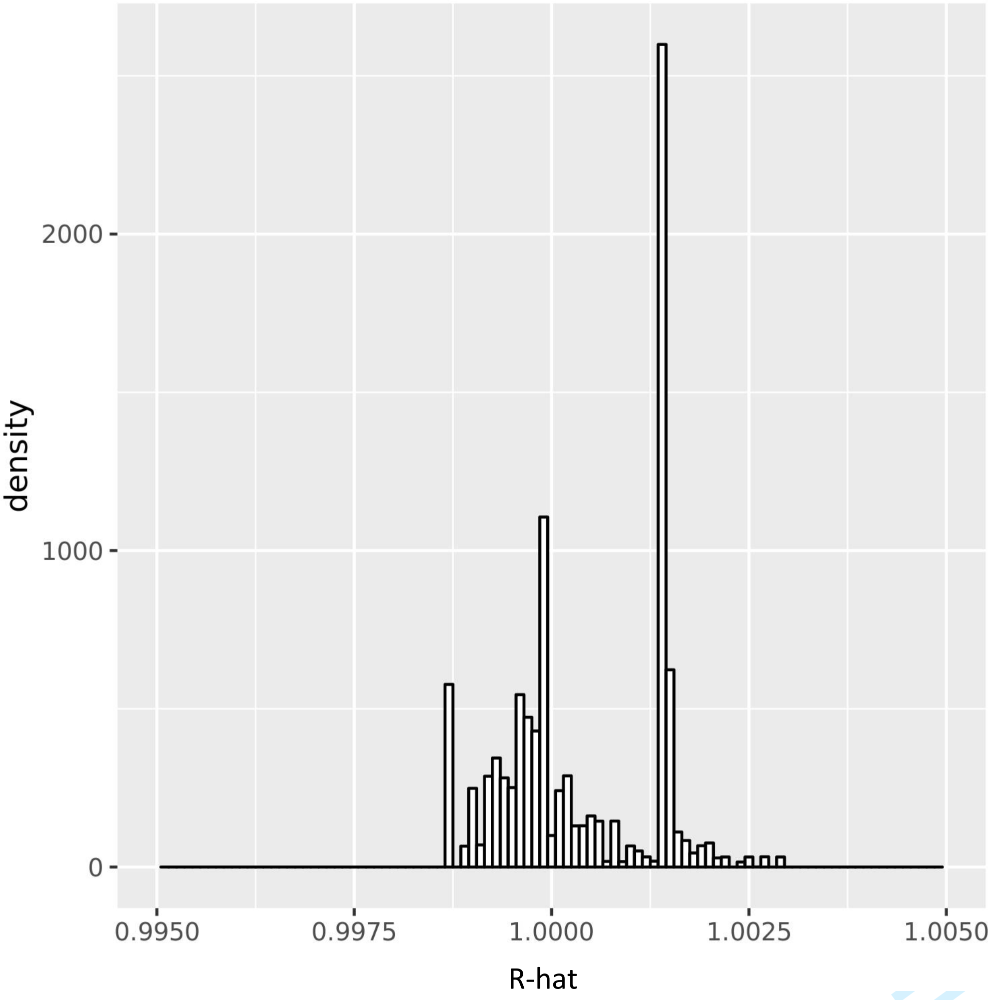

Supplementary Figure 1 represents the estimated R-hat for parameters of the final model. An R-hat near 1 suggests that between-chain variance for a given parameter is equal to the within-chain variance, suggesting convergence of the model. All values were well below 1.05.

Supplementary Figure 2: Pareto shape parameter k distribution for final model

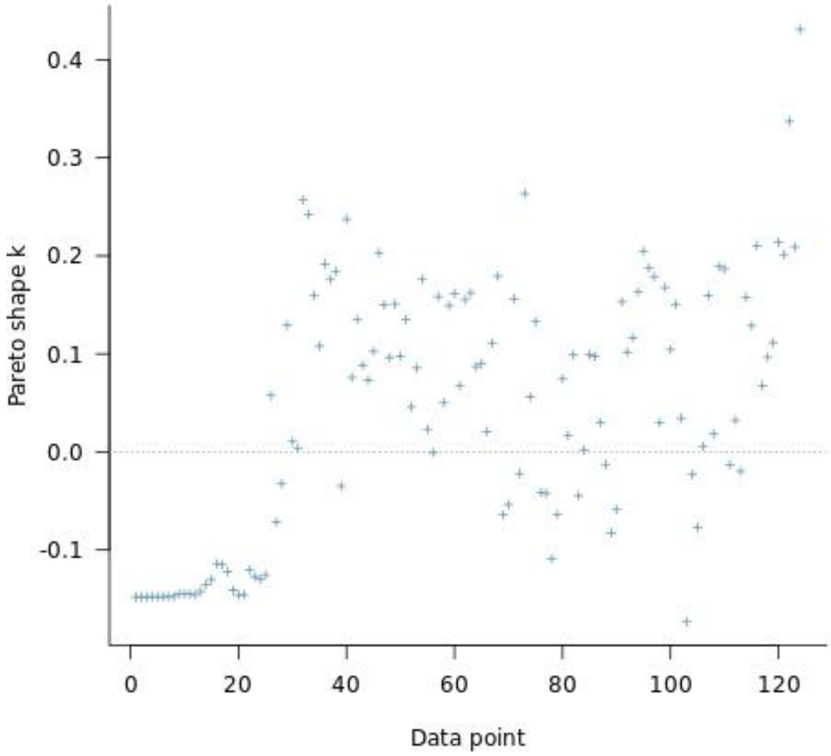

The estimated shape parameter k of the generalized Pareto distribution can be used to assess the reliability of the estimate from approximations of Leave-one-out cross-validation (LOO). The k shape values are all below 0.5, suggesting our estimates are reliable.

eview only

Supplementary Figure 3: Rt estimates with uninformative priors

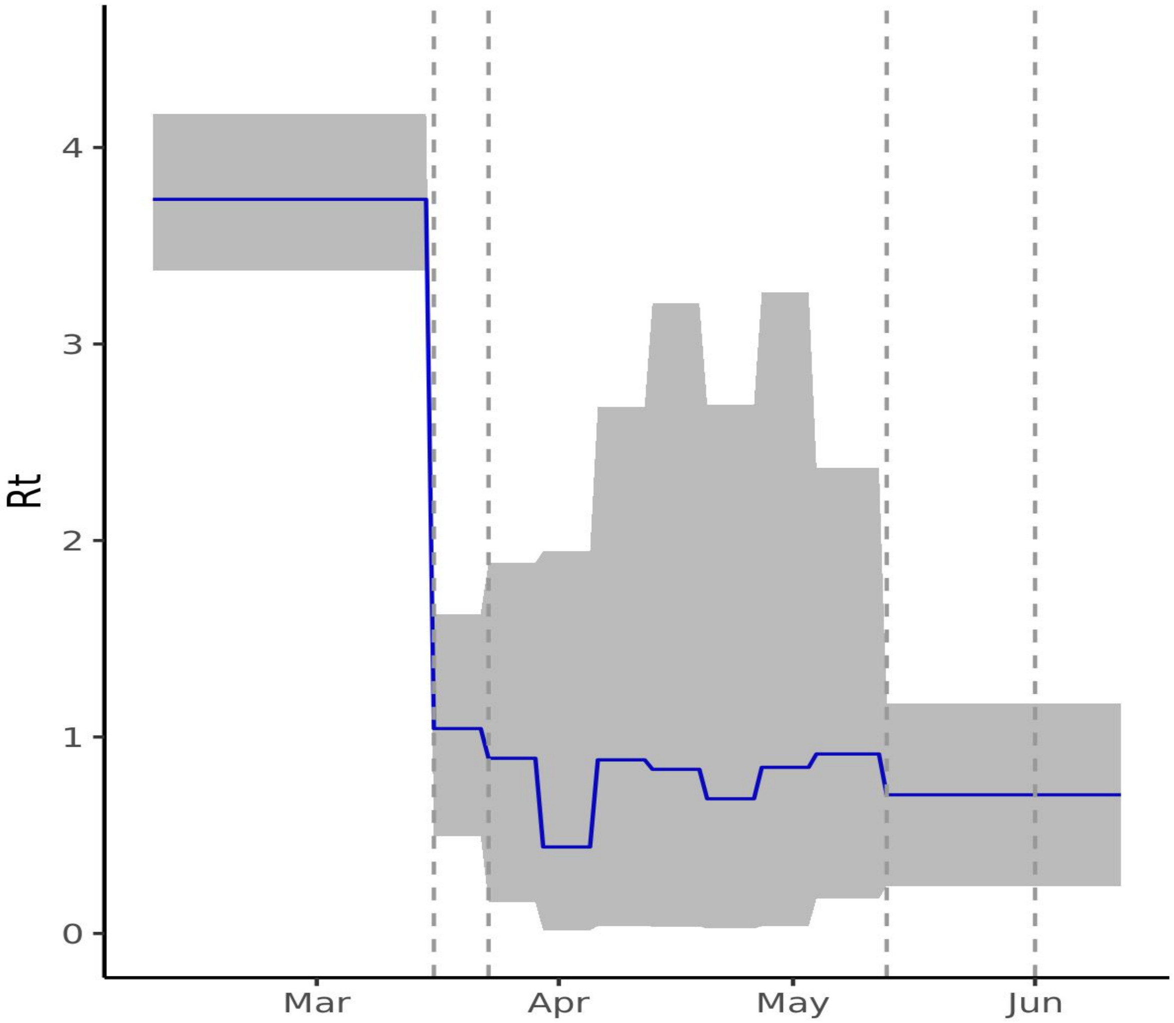

Supplementary Figure 3. Represents estimates of Rt when uninformative priors are used for estimation. We find that although uncertainty is greater around estimates, median estimates, and patterns of changes are similar as for the original model for all time intervals, suggesting that these are not constrained by specification of the prior in the final model.

Supplementary Figure 4: Estimated reproduction number in model with longer serial interval

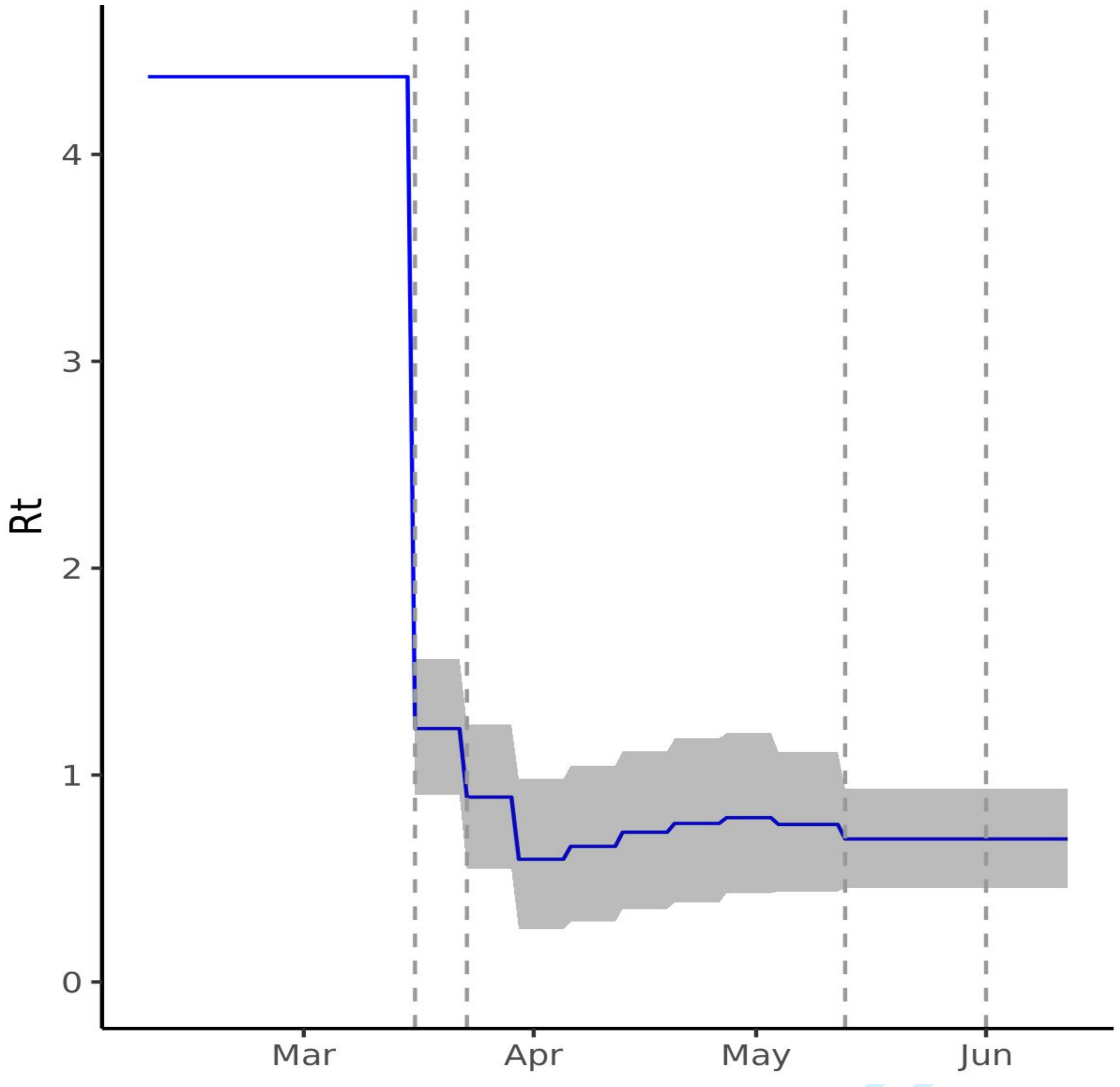

The figure shows the R_t estimated by a model with a a serial interval of mean 6.5 and coefficient of variation of 0.72. While estimates of R0 are higher in this model, estimates during other time intervals following lockdown are very similar to our primary model. 95% credible intervals are represented by grey bands.

Supplementary Figure 5: Predicted and observed deaths in model with longer serial interval

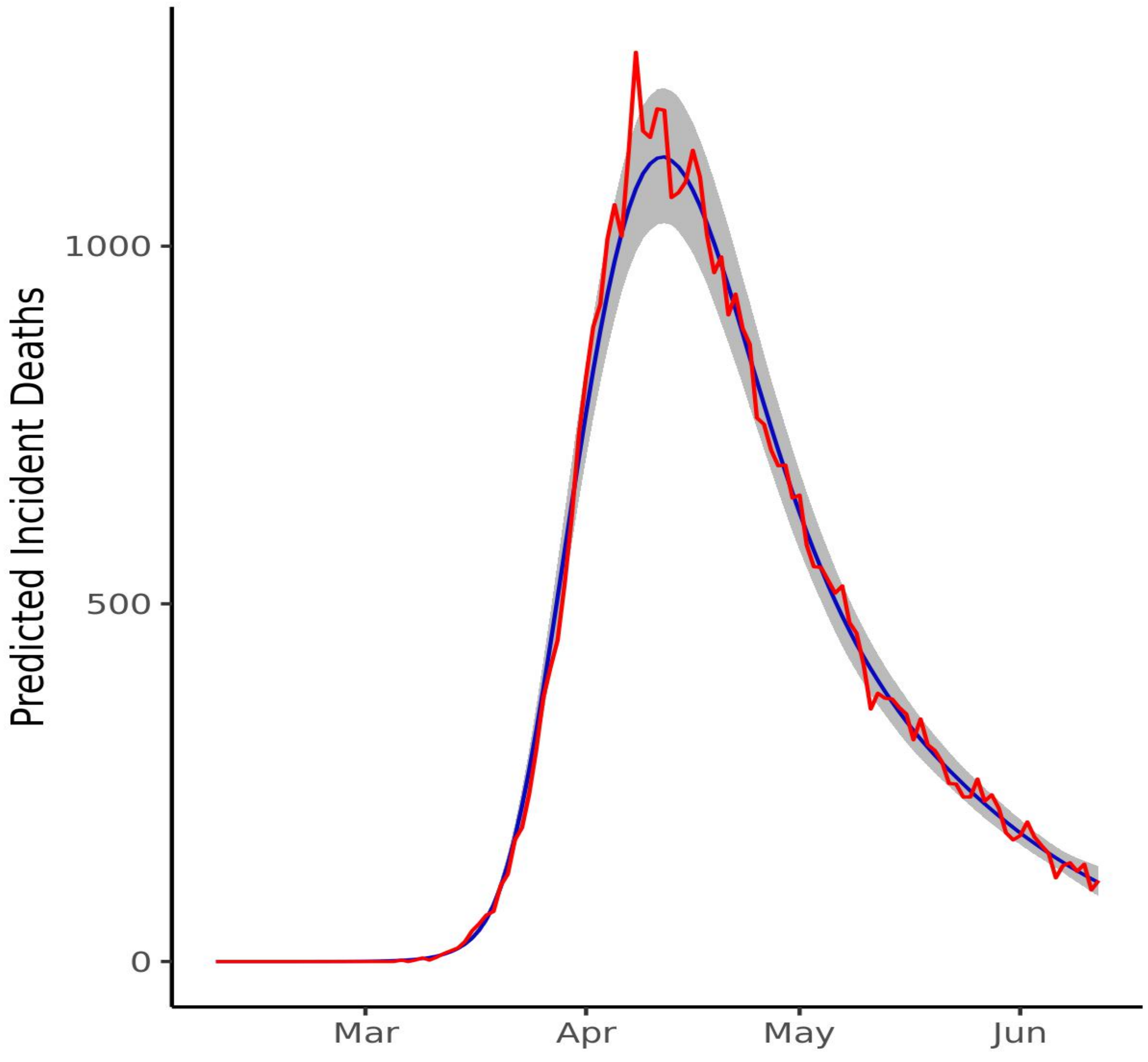

Daily deaths predicted by a model specifying longer serial intervals (blue) with 95% credible intervals (grey) show a good fit to the observed deaths from the ONS (red)

BMJ Open

Modelling the impact of lockdown easing measures on cumulative COVID-19 cases and deaths in England

Journal:	*BMJ Open*
Manuscript ID	bmjopen-2020-042483.R1
Article Type:	Original research
Date Submitted by the Author:	12-Oct-2020
Complete List of Authors:	Ziauddeen, Hisham; University of Cambridge<br>Subramaniam, Naresh; University of Cambridge<br>Gurdasani, Deepti; Queen Mary University of London, William Harvey Research Institute
**Primary Subject Heading**:	Epidemiology
Secondary Subject Heading:	Epidemiology
Keywords:	EPIDEMIOLOGY, Health policy < HEALTH SERVICES ADMINISTRATION & MANAGEMENT, Public health < INFECTIOUS DISEASES, Infection control < INFECTIOUS DISEASES

SCHOLARONE™
Manuscripts

I, the Submitting Author has the right to grant and does grant on behalf of all authors of the Work (as defined in the below author licence), an exclusive licence and/or a non-exclusive licence for contributions from authors who are: i) UK Crown employees; ii) where BMJ has agreed a CC-BY licence shall apply, and/or iii) in accordance with the terms applicable for US Federal Government officers or employees acting as part of their official duties; on a worldwide, perpetual, irrevocable, royalty-free basis to BMJ Publishing Group Ltd ("BMJ") its licensees and where the relevant Journal is co-owned by BMJ to the co-owners of the Journal, to publish the Work in this journal and any other BMJ products and to exploit all rights, as set out in our licence.

The Submitting Author accepts and understands that any supply made under these terms is made by BMJ to the Submitting Author unless you are acting as an employee on behalf of your employer or a postgraduate student of an affiliated institution which is paying any applicable article publishing charge ("APC") for Open Access articles. Where the Submitting Author wishes to make the Work available on an Open Access basis (and intends to pay the relevant APC), the terms of reuse of such Open Access shall be governed by a Creative Commons licence – details of these licences and which Creative Commons licence will apply to this Work are set out in our licence referred to above.

Other than as permitted in any relevant BMJ Author's Self Archiving Policies, I confirm this Work has not been accepted for publication elsewhere, is not being considered for publication elsewhere and does not duplicate material already published. I confirm all authors consent to publication of this Work and authorise the granting of this licence.

For peer review only

Modelling the impact of lockdown easing measures on cumulative COVID-19 cases and deaths in England

Ziauddeen H, PhD [1,2,3], Subramaniam N, BSc [1,2], Gurdasani D, PhD† [4].

[1] Dept. of Psychiatry, University of Cambridge, Cambridge UK

[2] Wellcome Trust-MRC Institute of Metabolic Science, University of Cambridge, Cambridge UK

[3] Cambridgeshire & Peterborough Foundation Trust, Cambridge UK

[4] William Harvey Research Institute, Barts and The London School of Medicine and Dentistry, Queen Mary University of London, London UK

† **Corresponding author**:

Dr Deepti Gurdasani
William Harvey Research Institute,
Barts and The London School of Medicine and Dentistry,
Queen Mary University of London, London UK
Email: d.gurdasani@qmul.ac.uk
Phone: 07737650357

For peer review only

Abstract:

Objectives:
To assess the potential impacts of successive lockdown easing measures in England, at a point in the COVID-19 pandemic when community transmission levels were relatively high.

Design:
We developed a Bayesian model to infer incident cases and *R* in England, from incident death data. We then used this to forecast excess cases and deaths in multiple plausible scenarios in which *R* increases at one or more time points.

Setting:
England

Participants:
Publicly available national incident death data for COVID-19 were examined.

Primary Outcome:
Excess cumulative cases and deaths forecast at 90 days, in simulated scenarios of plausible increases in *R* after successive easing of lockdown in England, compared to a baseline scenario where *R* remained constant.

Results:
Our model inferred an *R* of 0.75 on the 13th May when England first started easing lockdown. In the most conservative scenario modelled where *R* increased to 0.80 as lockdown was eased further on 1st June and then remained constant, the model predicted an excess 257 (95% 108-492) deaths and 26,447 (95% CI 11,105-50,549) cumulative cases over 90 days. In the scenario with maximal increases in *R* (but staying ≤1), the model predicts 3,174 (95% CI 1,334-6,060) excess cumulative deaths and 421,310 (95% CI 177,012-804,811) cases. Observed data from the forecasting period aligned most closely to the scenario in which *R* increased to 0.85 on the 1st June, and 0.9 on the 4th July.

Conclusions:
When levels of transmission are high, even small changes in *R* with easing of lockdown can have significant impacts on expected cases and deaths, even if *R* remains ≤1. This will have a major impact on population health, tracing systems and health care services in England. Following an elimination strategy rather than one of maintenance of *R*≤1 would substantially mitigate the impact of the COVID-19 epidemic within England.

Strengths and limitations

1. This study provides urgently needed information about the potential impact of successive lockdown easing measures in England when community transmission of SARS-CoV2 is relatively high.
2. We utilise a robust Bayesian model based on ONS registered deaths in England, to infer incident cases and reproduction number and then forecast deaths and cases

For peer review only

considering multiple plausible scenarios of increase in reproduction number with successive easing of lockdown in England.

3. Our study focuses on the impact of easing lockdown in the conservative scenario when R is maintained at or below 1 in line with stated government policy, showing that even this scenario would result in substantial excess of cases and deaths relative to a baseline scenario of not easing lockdown or elimination.
4. The excess cumulative cases are likely to be sensitive to the specified infection fatality ratio, although this is not expected to materially change the results and inferences. We have assumed a constant infection fatality rate across time, which would not account for changes in the age-composition of the infected cases over time.
5. The model inference in dependent on reliable reported statistics on incident deaths. Underestimation of recent registered deaths would lead to more conservative R inference, and underestimation of the impact of easing lockdown.

Introduction:

As countries around the world negotiated the first wave of the COVID-19 pandemic, governments had to make critical decisions about when and how they eased the lockdown measures instituted to control the pandemic. Given the significant risks of a resurgence of the pandemic and the consequent implications, these decisions have had important consequences on pandemic control following easing of lockdown restrictions globally.

Different countries eased lockdown in different ways, and at different points in their epidemic trajectory.[1] The UK imposed lockdown relatively late in its epidemic trajectory and began easing lockdown relatively early, when community transmission levels (incident cases) were still high.[2] By contrast, Germany, Denmark, Italy and Spain started easing lockdown when incident cases and deaths were at much lower levels. However despite mitigating strategies such as test, trace and isolation systems in place, countries like Germany saw increases in reproduction number (*R)* after easing lockdown, with increases to above 1 in June.[3] South Korea, and China too saw a resurgence in new cases after easing their lockdowns and went on to put in place localised restrictions to control the spread of infections.

Several experts, including SAGE, the scientific advisory body to the UK government, cautioned against easing lockdown in May 2020 [2], when community transmission was still high, warning that this could overwhelm the still nascent testing and contact tracing services that could mitigate the impact of easing lockdown, and greatly impact the health service. Nevertheless, the UK proceeded with easing lockdown with the stated aim of doing so while keeping $R \leq 1$. On the 13th May, people who could not work from home were asked to return to work. On the 1st June schools were re-opened, outdoor markets and showrooms opened and households were allowed to meet in socially distanced groups of six. On the 15th June non-essential businesses, including the retail sector, were opened. In the week of the 29th June, a surge in cases was reported in Leicester, England, leading to the re-imposition of restrictive measures, and concern that other regions in England may experience similar increases in case numbers.[4] Nevertheless, the government went ahead with the next planned easing of lockdown on the 4th of July, when pubs, cafes, and hotels opened.

For peer review only - http://bmjopen.bmj.com/site/about/guidelines.xhtml

As the country proceeded to rapidly ease lockdown, it was vital to understand and quantify the potential impact of this so as best inform public health strategy. In June 2020 we modeled these impacts across a range of plausible scenarios over the 90 day period from the 1st of June to the 29th of August. Using an epidemiological model of COVID-19 spread with Bayesian inference, we inferred parameters of the epidemic in England using daily death data from the Office of National Statistics (ONS). We estimated the time varying *R* and daily cases, and then used these to forecast cases and deaths in several plausible scenarios in which *R* increased with the easing of lockdown, particularly focusing on those in which *R* remained ≤1, and contrasted these with elimination strategies that aim to suppress *R* as much as possible.

During the manuscript review process, we were able to examine the observed data that accrued through the original forecasting period and compare it against the model predictions.

Methods

The original model inference and forecasting were carried out in June 2020 and the model development is described below. Following this, we describe the comparison of the model predictions from the original forecasts to the observed data from the forecasting period.

Data for model development:
In order to model the impact of easing lockdown, we needed to know the levels of transmission, and growth parameters of the regional epidemic. Given the limited community testing and case detection in the UK, incident case numbers at that point were likely to be substantially underestimated. We therefore based our model on the number of incident deaths by date of occurrence, which are likely to be more reliable.[5] Incident deaths are a function of incident cases in the previous weeks and the reproduction rate of the epidemic, and both these parameters can be inferred from the death data.[5] We included data till the 12th of June for England, as released by the ONS on the 30th of June 2020 (25th week of published data).[6] These data are based on deaths registered by the 27th of June. As reporting delays mean that more recent deaths are underestimated, we only considered deaths up to the 12th June.

Patient and public involvement
As only publicly available aggregate incident death statistics were utilised, there was no direct patient or public involvement.

Primary outcomes:
We assessed the excess cumulative predicted cases and deaths, over a 90-day period from the 1st June. We assumed different scenarios of changing *R* at the points of lockdown easing, in comparison with a baseline scenario in which *R* remained constant during this period.

Estimation of incident cases:
Incident cases, and time-varying *R* numbers were estimated using a Bayesian model, similar to that previously described by Flaxman et al,[5] accounting for the delay between onset of infection and death. The number of infected individuals is modelled using a discrete renewal process, as has been described before.[5] This is related to the commonly used Susceptible-Infected-Recovered (SIR) model, but is not expressed in differential form.

For peer review only - http://bmjopen.bmj.com/site/about/guidelines.xhtml

We modelled cases from 30 days prior to the first day that 10 cumulative deaths were observed in England, similar to previous methods.[5] The numbers of incident cases for the first 6 days of this period were set as parameters to be estimated by the model (**Table 1**).

Table 1: Parameters estimated by Bayesian model

Variable	Parameter	No.	Priors
c_t, where t=1…6	Number of initial cases on first six days	6	exponential(1.0/tau)
R_0	Baseline Reproduction Number	1	Normal(2.4,0.5)
R_t	Time varying effective reproduction number	9	Normal(0.8,0.25)
φ	variance parameter for negative binomial distribution of deaths	1	normal(0,5)
τ	parameter in prior of c_t	1	exponential(0.03)

Subsequent incident case numbers would then be a function of these initial cases, and estimated *R* values. We assumed a serial interval (SI) with a lognormal distribution with mean 4.7 and standard deviation (SD) of 2.9 days, as in Nishiura et al [7]. The SI was discretised as follows:

$$g_s = \int_{t=s-1}^{s} g(t)dt$$

For s=1,2…N, where N is the total number of intervals (each interval being 1 day) estimated. We estimated the distribution for 201 days, to align with the 111 days of data up to the 29th May, plus 90 days of forecasting. Given a SI distribution, the number of infections c_t on a given day *t*, is given by the following discrete convolution function:

$$c_t = R_t \sum_{j=0}^{t-1} c_j g_{t-j},$$

The incident cases on a given day *t,* are therefore a function of *R* at point *t* and incident cases up to time *t−1*, weighted by the distribution of the serial interval.

Estimation of time-varying reproduction number

The baseline reproduction number (R_0), and the subsequent time varying effective reproduction number (R_t) were estimated up to the 12th June. We allowed R_t to change on at least three points: (1) 16th March, when the UK first introduced social distancing measures; (2) 23rd March, when lockdown measures came into place with stay at home instructions and closures of schools and non-essential businesses; and (3) 13th May, the first easing of lockdown. We also considered models in which R_t was allowed to change on the 1st June. Given the limited death data i.e. only up to the 12th June, we were unlikely to be able to estimate changes in R_t after the 13th May with sufficient certainty. Observed deaths from the

For peer review only - http://bmjopen.bmj.com/site/about/guidelines.xhtml

1st June are likely to be a function of cases 2-3 weeks prior to this, and were unlikely to reflect changes in R_t from the 1st of June.

Model selection

We assessed and compared models that allowed R_t to change at the 4 points described above (Model 1), with more flexible models that allowed more frequent changes (Models 2 and 3), as follows:

1. Model 1: 16th March, 23rd March, 13th May and 1st June
2. Model 2: Every week from the beginning of the modelling period, including on the 16th March, 23rd March, 13th May and the 1st June
3. Model 3: 16th March, 23rd March, and 13th May, and every week between the 23rd March and 13th May i.e. during lockdown.

For each model, we used the R package *loo* to calculate expected log pointwise predictive density (ELPD) using Leave-one-out cross-validation (LOO) individually for each left out data point based on the model fit to the other data points. We then calculated between-model differences in ELPDs, to assess whether particular models predicted data better than others, as discussed previously.[8] As the assumptions in estimation of ELPD may be violated given these are time-series data, and therefore correlated, we also compared the root mean squared errors (RMSE) across models to assess fit. The final model used was arrived upon based on these comparisons, prioritising differences in ELPD, as this has been used in a similar context to assess change points, previously.[9] We assessed whether models were significantly different (ELPD difference/SE of difference >2). When models were not statistically significantly different in performance, for simplicity, we prioritised the model where the least number of parameters needed estimation.

In addition, we also compared Model 1 (four change points) with models where each of the change points were left out in turn, as done by Dehnig et al,[9] to assess if these dates do correspond to change points in R_t.

Estimation of deaths:

Incident deaths from COVID-19 are a function of the infection fatality rate (IFR), the proportion of infections that result in death, and incident cases that have occurred over the past 2-3 weeks. For observed daily deaths (D_t) for days $t \in 1, \ldots, n$, the expectation of observed daily deaths (d_t) is given by:

$$d_t = \mathrm{E}(D_t)$$

As described in Flaxman et al., we model the number of observed daily deaths D_t as following a negative binomial distribution with mean d_t and variance $d_t + \frac{d_t^2}{\psi}$, where ψ follows a half normal distribution:

$$D_t \sim Negative\ Binomial\left(d_t,\ d_t + \frac{d_t^2}{\psi}\right), \quad \text{where } \psi \sim Normal^{+}(0,5).$$

For peer review only - http://bmjopen.bmj.com/site/about/guidelines.xhtml

Similar to estimation of incident cases, deaths at time point t (d_t) were modelled as a function of incident cases up to time $t-1$, weighted by the distribution of time of infection to time of death (π). The π distribution was modelled as the sum of the distribution of infection onset to symptom onset (the incubation period), and the distribution of symptom onset to death. As has been previously done,[5] both of these were modelled as gamma distributions with means of 5.1 days (coefficient of variation 0.86) and 18.8 days (coefficient of variation 0.45), respectively as follows:

$$\pi \sim IFR * (Gamma(5.1, 0.86) + Gamma(18.8, 0.45))$$

IFR was assumed to be 1.1%, based on the most recent estimates from the University of Cambridge MRC Nowcasting and Forecasting model.[10] This estimate is in line with estimates from Flaxman et al. (Imperial), of 1% that have been widely used in modelling of COVID-19 deaths across the UK.[11] These estimates are based on the those reported by Verity et al.,[12] during early epidemiological inference from the outbreak in Wuhan, and are corrected for age structure, and contact patterns for the UK, as previously outlined.[11] Misspecification of the IFR estimate would lead to biased inference of case numbers, but not deaths, as this can be considered as a scaling factor, that is used first to estimate the cases, which are then used to accurately predict observed deaths, and future deaths based on different scenarios. Therefore, the predicted death numbers can be thought of as independent of these estimates. For simplicity, we consider a fixed IFR over time.

To discretise the time to death distribution, we estimated the probability of death within each discrete time interval (1 day), conditional on surviving previous intervals. First, we calculate the hazard (h_t) the instantaneous probability of failure (i.e. dying) within a time interval, as follows:

$$h_t = \frac{\int_{t = s - 0.5}^{s + 0.5} \pi(t) dt}{1 - \pi_{s - 0.5}}$$

As the denominator excludes individuals who have died, this ensures that h_t is calculated only among those surviving. The probability of survival within each interval is:

$$s_t = 1 - h_t$$

The cumulative survival probability of surviving up to the interval $t-1$ is therefore:

$$S_{T > t - 1} = \prod_{j = 1}^{t - 1} s_j$$

, where T is the time of death of an individual. In other words the cumulative probability of survival up to interval t is simply the product of survival within each interval up to $t-1$, where the probability of survival within each interval (s_t) is $1-h_t$, where h_t is the probability of dying within that interval.

Given this, we now estimate the probability of death within interval t, conditional on surviving up to $t-1$ as:

For peer review only - http://bmjopen.bmj.com/site/about/guidelines.xhtml

$$\omega_t = P(T = t | T > t-1) = S_{T > t-1} * h_t$$

Here ω represents the discretised distribution of infection onset to death, with the probability of death within interval *t* conditional on surviving previous intervals. Deaths can therefore be calculated as a function of incident cases of infection within previous intervals, as follows:

$$d_t = \sum_{j=0}^{t-1} c_j \omega_{t-j}$$

Here, the number of deaths within interval *t* (on a given day) is a sum of the number of daily cases up to the previous day, with previous cases weighted by the discretised probability distribution of time from onset of infection to death.

Estimated parameters and model priors:
We estimated the set of model parameters θ={$c_{1\text{-}6}$, R_0, R_t, φ, $\boldsymbol{\tau}$} using Bayesian inference with Markov-chain Monte-Carlo (MCMC) (**Table 1**). We estimated the number of cases in the first six days of the modelled period, as subsequent cases are simply a function of cases on these days, the SI, and R_t . As described above, R_0 was constrained up to the 16th March and then again after the 13th of May. For the period prior to 16th March, we assigned a normal prior for R_0 with mean 2.5 and SD 0.5. For the period that R_t was allowed to vary i.e. every week from the 16th of March till the 13th of May, we assigned a normal prior with a mean 0.8 and SD 0.25. These priors are based on estimates of time changing R_t from the University of Cambridge MRC biostatistics nowcasting and forecasting models[10] and SAGE estimates of *R*,[13] and consistent with Flaxman et al.[5] For the number of cases on day 1, we assigned a prior exponential distribution:

$$y \sim exponential\left(\frac{1}{\tau}\right)$$

where $\tau \sim exponential(0.03)$

Model estimation:
Parameters were estimated using the Stan package in R with Markov chain Monte Carlo (MCMC) algorithms used to approximate a posterior distribution of parameters by randomly sampling the parameter space. We used 4 chains with 1000 warm up samples (which were discarded), and 3000 subsequent samples in each chain (12,000 samples in total) to approximate a posterior distribution using the Gibbs Sampling algorithm. From these we obtained the best-fit values and the 95% credible intervals for all parameters. We used these parameters to estimate the number of incident cases and deaths in England. We examined the fit of the model predicted deaths to the observed daily deaths from the ONS, and also the consistency of the model parameters with known values in the literature, estimated from global data. We assessed the distribution of *R-hat* values for all parameters, to assess convergence between chains.

Sensitivity analyses:
We carried out sensitivity analyses using uninformative priors for R_0 and R_t, to examine the sensitivity of R_t estimates to prior specification. We also examined the impact of the SI by comparing the baseline model (SI of mean 4.7 and SD 2.9 days), with a longer SI modelled as

For peer review only - http://bmjopen.bmj.com/site/about/guidelines.xhtml

a gamma distribution with mean 6.5 and coefficient of variation of 0.72, as estimated by Chan et al.[14]

Forecasting cases and deaths:

All forecasts were carried out up to 90 days (29th August 2020) after the 1st of June. We considered a set of scenarios in which R_t increased from baseline on the 1st of June and then remained constant, as well as those in which further increases in R_t occur on the 15th June and the 4th July. We considered an increase in R_t of up to 0.25 in increments of 0.05, this being a plausible degree of change in response to easing lockdown, based on the empirical data from other countries,[3,15] as well as the modelling by UK SAGE.[16] Finally, for comparison with a strategy of elimination, namely suppressing R_t to the lowest level possible before easing lockdown measures, as has been done South Korea, New Zealand and Australia, we also modelled scenarios with R_t values of 0.6 and 0.7.

For each of these scenarios, we predicted the number of incident cases, and incident deaths, using the functions from the inference model above. Briefly cases are a function of R_t, incident cases on previous days and the SI discretised distribution:

$$c_t = R_t \sum_{j=0}^{t-1} c_j g_{t-j},$$

Deaths are a function of incident cases over previous weeks, and the distribution of onset of infection to death times:

$$d_t = \sum_{j=0}^{t-1} c_j \omega_{t-j}$$

All scenarios were compared to a baseline scenario of no change in R_t from the 13th of May onwards.

Comparison of model predictions to observed data:

The observed death data for daily deaths in England up to the 28th of August as obtained from the ONS (from data up to the 11th September) were plotted against the original model predictions from June, and the root mean square error was calculated between the observed data and the predicted deaths in the different modelled scenarios. The model was rerun with these data, to infer values of R_t till the 28th of August. As the purpose of this exploratory model was inference of parameters, R_t was allowed to change weekly from the 16th March, as well as at time points of easing lockdown: 13th of May, 1st June, 15th June and 4th July as in the original forecasting and the 25th of July (gyms and pools reopened), and the 15th of August (casinos, bowling alleys and soft play areas reopened). Where these dates fell on the weekly change point, they were not included separately.

Results

Model selection and model inferences

Model 3, which allowed weekly changes in R_t during lockdown, produced the best fit to the data (**Supplementary Table 1**), with estimation of fewer parameters compared with Model 2.

For peer review only - http://bmjopen.bmj.com/site/about/guidelines.xhtml

This was therefore used as the primary model and unless otherwise stated, all inferences described subsequently are from this model.

We inferred R_0 of 3.65 (95% credible intervals (CI) 3.36-3.96), consistent with previous estimates within the UK.[5] The R_t is estimated to have declined substantially following initiation of social distancing, and lockdown measures, reaching a low of 0.66 (95% CI 0.34-1.04) during the week 30th March-5th April 2020. The most recent R_t from the 13th of May is estimated as 0.752 (95% CI 0.50-1.00) (**Figure 1**). The alternative models allowing change of R_t on the 1st of June inferred a very similar R_t for the 1st -12th June suggesting that there was insufficient data to accurately infer any changes to R_t following the easing of lockdown on 1st June. On examining the impact of constraining R_t on model fit at any of the 4 change points, this appears greatest for the 16th March (when social distancing measures were put into place) (**Supplementary Table 2**) with only modest impacts on model fit of constraining R_t on 23rd March and 13th May, and no impact on constraining R_t on the 1st June.

The model showed a good fit to the observed distribution of deaths up to the 12th June (**Figure 2**). *Rhat* estimates were < 1.05 for all estimated parameters (**Supplementary Figure 1**). Leave one out cross-validation also supported a good model fit, with the shape parameter k<0.5 for all values (**Supplementary Figure 2**). The median number of incident cases inferred on the 1st June was 4,317/day (95% CI 2,062-8,155), which was broadly consistent with the estimates from the ONS survey for England based on a random sample of the population within the same time period.

Forecasts of lockdown easing scenarios

In the baseline forecasting scenario where R_t remained constant (R_{test}=0.75) through the 90-day forecasting period (1st June to 29th August 2020), the model predicted 48,501 (46,170-50,989) cumulative deaths in England (**Supplementary Table 3**). By comparison, the ONS reported 46,539 cumulative deaths up to 12th June in England (registered up to 27th June).

In the scenarios where R_t increased on the 1st of June and then remained constant, for increases from the median 0.75 to 0.80, 0.85, 0.90, 0.95 and 1, the model predicted median excess deaths of 257 (95% CI 108-492), 632 (95% CI 265-1,208), 1,173 (95% 493-2,240), 1,971(95% 828-3,764) and 3,174 (95% CI 1,334-6,060) respectively. Increases of R_t to 1.05 and 1.1, with resultant exponential growth, led to excess median deaths of 5017 (95% CI 2,109-9,578), and 7,878 (3,313-15,037) respectively (**Figure 3** and **Supplementary Table 3**).

In scenarios where R_t increased on the 1st June, 15th June and 4th July, we found that compared to the baseline scenario, modest increases of R_t to 0.80, 0.85, 0.90, on these dates respectively would lead to 508 (95% CI 213-972) excess deaths. If R_t increased to 0.90, 0.95 and 1 at these time points, then excess estimated deaths increase to 1,848 (95% CI 776-3,534). In these scenarios R_t remains ≤1 (**Figures 3-5** and **Supplementary Table 3**). Increases of R_t above 1 at any point resulted in rapid increases in cases, and deaths, with between 3,600-13,000 excess deaths in different scenarios for R_t rising up to between 1 and 1.2, predicting a second wave of the epidemic within England (**Figure 4-5** and **Supplementary Table 3**).

Even in the conservative scenario where R_t increased from 0.75 to 0.80 on the 1st of June and then remained constant thereafter, the model predicted an excess of 26,447 (95% CI 11,105-

For peer review only - http://bmjopen.bmj.com/site/about/guidelines.xhtml

50,549) cumulative cases over 90 days. On the other hand, the scenario with the largest changes in R_t, but still remaining ≤1, predicted an excess of up to 421,310 (95% CI 177,012-804,811) (**Figures 6-8** and **Supplementary Table 3**). For scenarios where R_t rose beyond 1 (up to 1.2), we would expect between 540,000 to 2.8 million excess cases, in line with a second wave (**Supplementary Table 3**).

Forecasts from an elimination scenario

Compared to the baseline scenario of R_t staying at 0.75, we found that maintaining R_t at 0.60 and 0.70 would result in 44,302(95% CI 84684-18600) and 19,968 (95% CI 38168-8384) fewer cumulative cases, and 462 (95% CI 194-884) and 204 (95% CI 389-86) fewer deaths over the modelled 90-day period, respectively **(Figure 3, Figure 6, Supplementary Table 3**).

Robustness of model in sensitivity analyses

Using uninformative (no prior specified) priors for R_t did not materially alter the median estimates of R_t, although uncertainty around estimates was predictably increased (**Supplementary Figure 3**). This suggests our estimates are robust to the priors specified.

Using a longer SI leads to an increase in the estimated R_0, although subsequent estimates following easing of lockdown remain broadly comparable (**Supplementary Figure 4**). This model is comparable to the primary model with regard to fit to observed deaths (**Supplementary Figure 5**) although we note that predicted excess deaths and cases in all scenarios where $R_t<1.1$, are higher than in the primary model with shorter serial interval (**Supplementary Table 4**), suggesting the primary model is likely to be conservative.

Comparison of model predictions to observed data:

The observed cases and deaths are plotted against the modelled scenarios in **Figure 9**. Among the scenarios studied, the observed daily deaths seems to align most closely with the scenario in which R values are 0.85, 0.85 and 0.9 at the 3 change points. The RMSE between the observed and predicted deaths is lowest for this scenario (**Supplementary Table 3**). The inferred R_t values concur with this (although uncertainty estimates are wide), and also suggest that it is in late July that R_t started to creep above 1 (**Figure 10**). We also note that the observed cumulative deaths by the 28th August represent an excess of 1,291 deaths over our baseline scenario.

Discussion

In this paper we describe a Bayesian model for inferring incident cases and reproduction numbers from daily death data, and for forecasting the impact of future changes in *R*. Our findings provide important quantification of the likely impact of relaxing lockdown measures in England, and to our knowledge, this is the first study to have comprehensively assessed this through several plausible scenarios. We show that even in scenarios in which *R* remains ≤1 (in line with the UK government's stated aim), small increases in R_t from lifting lockdown measures, can lead to a substantial excess of deaths with 3,174 (95% CI 1,334-6,060) in the most severe scenario modelled.

For peer review only - http://bmjopen.bmj.com/site/about/guidelines.xhtml

Our model inferences are robust to modelling assumptions of specified priors for R_t. We note, however, that using a longer serial interval would results in a higher numbers of excess deaths for each scenario, suggesting that our primary scenario is conservative (**Supplementary Tables 3** and **4**). Our estimated R_t of 0.75 following 13th May is consistent with estimates from the SAGE group advising government at the time.[13] We assessed increases in R_t that were entirely plausible, given the data from other European countries that have started easing lockdown.[3] Our model predicted a substantial excess of cases and deaths in several scenarios where R remained ≤1, as well as scenarios where R increased up to 1.2. When we compared our predictions to the observed data from the original forecasting period we found that these aligned most closely to the scenario in which R increased to 0.85 on the 1st June, and then to 0.9 on the 4th July. In contrast, our model showed that had an elimination strategy been pursued and R_t suppressed to 0.6 or 0.7, this could have prevented a median estimated 462 and 204 deaths, and 44,302, and 19,968 cases, respectively from the baseline scenario.

Countries like Denmark and Germany started easing lockdown when community transmission was low and this likely mitigated increases in R with the lifting of lockdowns, alongside the use of aggressive case detection and contact tracing approaches. The UK began to ease lockdown when community transmission was still high (with daily estimated >8000 cases and >300 deaths) and still does not have a fully operational test, trace and isolate system at the time of writing, with the existing system overwhelmed by incident cases. The UK's current estimates of R_t still rely on incident deaths (as used by the MRC Nowcasting and Forecasting model, and SAGE)[10] , and therefore reflect community transmission from a median of 2-3 weeks ago.[13] Easing lockdown in 2-weekly steps, meant that by the time we detected the impact of one step, the next one had already been instituted and not unexpectedly, mitigating these impacts was challenging. At the time lockdown was being rapidly eased UK SAGE expressed concerns that increases in R up to 1.2 could continue undetected for longer periods of time.[16]

In September 2020, the UK is at point where community transmission is once again high and it is clear that we have entered the second wave of the pandemic. Schools reopened in the second week of September, a move that is vitally important to children's health and development, but one that can potentially increase community transmission. Cases and hospitalisations have been increasing exponentially, which has recently translated into an increase in weekly deaths. Using the best available confirmed COVID-19 case data in England published by the UK government on the 21st September (which is likely an underestimate), we modelled the potential impact of increases in transmission on daily cases and deaths over the next two months, assessing different scenarios of increase in R_t. As R_t reaches 1.5, the daily deaths approach 1,000 by late November (**Figure 11**). We note that the number of deaths forecast during this period could be overestimated if transmission is disproportionately higher among younger age groups, as overall IFR would be lower than the assumed 1%. However, as current data suggests, transmission is likely to spill over into more vulnerable, and older age groups over time. This has profound implications for the health service and the limited ICU capacity available in the NHS, which is at great risk of being overwhelmed. Our modelling suggests that small changes in R_t moving forward could have substantially large effects on case numbers, and deaths, suggesting that mitigatory strategies implemented in a timely manner could have a large impact.

For peer review only

We acknowledge some important limitations of our model. The first is that it is based on a back calculation of cases based on incident deaths, which are likely to underestimated due to reporting delays and underreporting. Second, our model is reliant on inferring cases, and reproduction numbers, which depend on the assumed distributions of the serial interval, and the time of onset to death distributions. Though we based our assumptions on the literature, misspecification of these would influence our estimates. While we have evaluated this, greater deviations from true estimates would make our forecasting less reliable. Third, similar to Flaxman et al,[11] our model uses the IFR as a multiplier for the distribution of time from infection to death, in the absence of reliable population level case fatality rates (CFR). While this would not affect the estimation of deaths, if the CFR were higher (due to large proportions of cases being asymptomatic), then the predicted case numbers would be overestimated by our model. We note, however that the estimate of IFR we used (1.1%) is consistent with the CFR estimated previously from Beijing[17] and Flaxman et al.[11] We have also, for simplicity, assumed that IFR remains constant throughout the pandemic and the forecasting period. Given that age is an important determinant of mortality, our model may not reflect the changes in the age-composition of infected individuals, and changes in healthcare, and treatments over time, influencing the accuracy of inference, and forecasting. Unfortunately, the ONS does not provide age-stratified daily death data for England to allow us to model differences in age-structure. We have therefore, not considered these in our inference or forecasting. We note that if cases occur disproportionately in younger populations following easing of lockdown, excess deaths may be overestimated during our forecasting period. Fourth, we did not consider the impact of mitigatory measures in our current modelling. However, as we have seen, mitigatory measures were implemented with significant delays from when community transmission increased, as many experts had expected. Nevertheless if implemented with sufficient rigour and coverage, mitigatory measures would reduce the impact of the modelled scenarios. We note that our inferred R_t based on recent death data should reflect the impact of mitigatory measures, such as testing, contract tracing and isolating, as well as mask use, as inferred R_t values were allowed to change every week. Finally, we only modelled a limited set of scenarios, mainly restricted to those in which R_t remained ≤ 1.2 but there are multiple possible scenarios that could be modelled. We note that the scenarios modelled are in line with R_t ranges that were subsequently inferred from current death data.

In summary, we show that increases in R_t as a result of easing lockdown would have a substantial impact on incident transmission and deaths for even modest increases that still maintain $R_t \leq 1$, and an even greater impact should R_t rise above 1. This has subsequently been borne out by the observed data. As we enter the second wave of COVID-19 in the UK, our findings and the observed data thus far argue strongly for a much more cautious approach in public health management, an urgent need for a properly functioning test, trace and isolate system and serious consideration of elimination strategy to control the pandemic.

Authors' contributions:

DG conceived the study and designed the model with NS. DG programmed the model and made the figures. HZ and NS consulted on the model design. All authors interpreted the results, contributed to writing the Article, and approved the final version for submission.

Declaration of interests:

For peer review only - http://bmjopen.bmj.com/site/about/guidelines.xhtml

None.

Data sharing:
All data on daily deaths used in this study were taken from the Office of National Statistics website (https://www.ons.gov.uk/peoplepopulationandcommunity/birthsdeathsandmarriages/deaths/datasets/weeklyprovisionalfiguresondeathsregisteredinenglandandwales).

The code for the model, and dataset analysed is available at: https://github.com/dgurdasani1/lockdownsim

Ethics
No ethical approval was obtained for this study, as only publicly available aggregate data on incident deaths was analysed.

Funding
HZ is partly funded by the Bernard Wolfe Health Neuroscience Fund at the University of Cambridge. NS is funded by a Strategic Award from the Wellcome Trust. DG is funded by the UKRI/Rutherford HDR-UK fellowship programme (reference MR/S003711/2).

Acknowledgements
We would like to particularly acknowledge and thank Flaxman et al and the team at Imperial College London for making their code available for us to use.

References:

1. Coronavirus: How lockdown is being lifted across Europe. BBC. 2020.
2. Gallagher J. Coronavirus: Risk in UK lockdown easing too soon, warn scientists. BBC. 2020.
3. Robert Koch Institute G. Coronavirus Disease 2019 (COVID-19), Daily Situation Report of the Robert Koch Institute 2020.
4. Leicester lockdown tightened as coronavirus cases rise. BBC. 2020.
5. Flaxman S MS, Gandy, A, Unwin HJT, Coupland H, Mellan, TA, Zhu H, Berah T, Eaton JW Report 13: Estimating the number of infections and the impact of non-pharmaceutical interventions on COVID-19 in 11 European countries: Imperial College, London, 2020.
6. Deaths registered weekly in England and Wales, provisional. In: Statistics OoN, editor.; 2020.
7. Nishiura H, Linton NM, Akhmetzhanov AR. Serial interval of novel coronavirus (COVID-19) infections. *Int J Infect Dis* 2020; **93**: 284-6.
8. Vehtari A, Gelman, A., and Gabry, J. Practical Bayesian model evaluation using leave-one-out cross-validation and WAIC. Statistics and Computing. *Statistics and Computing* 2017; **27**(5): 1413-32.
9. Dehning J, Zierenberg J, Spitzner FP, et al. Inferring change points in the spread of COVID-19 reveals the effectiveness of interventions. *Science* 2020.
10. Joint PHE Modelling Cell MBUC-WG. COVID-19: nowcast and forecast, 2020.
11. Flaxman S, Mishra S, Gandy A, et al. Estimating the effects of non-pharmaceutical interventions on COVID-19 in Europe. *Nature* 2020.

12. Verity R, Okell LC, Dorigatti I, et al. Estimates of the severity of coronavirus disease 2019: a model-based analysis. *Lancet Infect Dis* 2020; **20**(6): 669-77.
13. Gov.uk. Government publishes coronavirus (COVID-19) R value and growth rates. 2020.
14. Chan Y-W D FS, Lam T-L T, Leung M-H J, Wong M-L, Lam, H-Y, Chuang S-K. Transmission dynamics, serial interval and epidemiology of COVID-19 diseases in Hong Kong under different control measures. Wellcome Open Research; 2020.
15. STATUS FOR SMITTETRYKKET I DANMARK, 29/4 2020: Statens Serum Institut.
16. SAGE. Thirty-eighth SAGE meeting on COVID-19, 21st May 2020, 2020.
17. Leung K, Wu JT, Liu D, Leung GM. First-wave COVID-19 transmissibility and severity in China outside Hubei after control measures, and second-wave scenario planning: a modelling impact assessment. *Lancet* 2020; **395**(10233): 1382-93.

Figure Legends

Figure 1: Estimated time-varying reproduction number (R_t) for England

The figure shows the R_t estimated by Model 3 (blue) with 95% credible intervals (grey) with a serial interval of mean 4.7 and SD 2.9 days. From 3.65 (CI 3.36-3.96), R_t drops on the 16th March and 23rd March (indicated by vertical dashed lines) when social distancing and lockdown were instituted, reaching a low of 0.66 (95% CI 0.34-1.04) in the week of the 30th March. The last estimated R_t is 0.75 (95% CI 0.50-1.00) following the 13th May.

Figure 2: Model fit to observed death data

Daily deaths predicted by Model 3 (blue) with 95% credible intervals (grey) show a good fit to the observed deaths from the ONS (red)

Figure 3. Predicted deaths with R_t increasing on 1st June

(A) The model compared scenarios in which R_t increases to 0.80 (light green), 0.85 (green), 0.90 (dark blue), 0.95 (red), 1 (purple) and 1.05 (brown) and then remains constant for the 90-day forecasting period. The comparator baseline scenario is of R_t remaining at 0.75 (black) and two elimination strategies of R_t reducing to 0.7 (yellow) and 0.6 (light blue) were also considered. Vertical dashed lines represent time-points of easing lockdown. (B), (C) the incident and cumulative deaths increase in all scenarios in which R_t increases and reduces in the two elimination scenarios.

Figure 4. Predicted deaths in scenarios of R_t increase on 1st and 15th June compared with baseline scenario

(A) The model compared scenarios in which R_t increases to 0.80 (light green), 0.85 (green), 0.90 (blue), 0.95 (red), 1 (purple) and 1.05(brown) and then further by 0.05 on the 15th June and then remaining constant for the 90-day forecasting period. The comparator baseline scenario is of R_t remaining at 0.75 (black). Vertical dashed lines represent time-points of

For peer review only - http://bmjopen.bmj.com/site/about/guidelines.xhtml

easing lockdown. (B), (C) The incident and cumulative deaths increase in all scenarios in which R_t increases.

Figure 5. Predicted deaths in scenarios of R_t increase on 1st June, 15th June and 4th July compared with baseline scenario

(A) The model compared scenarios in which R_t increases to 0.80 (light green), 0.85 (green), 0.90 (blue), 0.95 (red), 1 (purple) and 1.05(brown) and then further by 0.05 on the 15th June and then again by 0.05 on the 3rd July before remaining constant for the 90-day forecasting period. The comparator baseline scenario is of R_t remaining at 0.752 (black). Vertical dashed lines represent time-points of easing lockdown. (B), (C) The incident and cumulative deaths increase in all scenarios in which R_t increases.

Figure 6. Predicted cases in scenarios of R_t increase on 1st June compared with baseline and elimination scenarios

(A) The model compared scenarios in which R_t increases to 0.80 (light green), 0.85 (green), 0.90 (dark blue), 0.95 (red), 1 (purple) and 1.05(brown) and then remains constant for the 90-day forecasting period. The comparator baseline scenario is of R_t remaining at 0.752 (black) and two elimination strategies of R_t reducing to 0.7 (yellow) and 0.6(light blue) were also considered. Vertical dashed lines represent time-points of easing lockdown. (B), (C) the incident and cumulative cases increase in all scenarios in which R_t increases and reduces in the two elimination scenarios.

Figure 7. Predicted cases in scenarios of R_t increase on 1st June and 15th June compared with the baseline scenario

(A) The model compared scenarios in which R_t increases to 0.80 (light green), 0.85 (green), 0.90 (blue), 0.95 (red), 1 (purple) and 1.05(brown) and then further by 0.05 on the 15th June and then remaining constant for the 90-day forecasting period. The comparator baseline scenario is of R_t remaining at 0.752 (black). Vertical dashed lines represent time-points of easing lockdown. (B), (C) The incident and cases increase in all scenarios in which R_t increases.

Figure 8. Predicted cases in scenarios of R_t increase on 1st June and 15th June and 4th July compared with the baseline scenario

(A) The model compared scenarios in which R_t increases to 0.80 (light green), 0.85 (green), 0.90 (blue), 0.95 (red), 1 (purple) and 1.05(brown) and then further by 0.05 on the 15th June and then again by 0.05 on the 3rd July before remaining constant for the 90-day forecasting period. The comparator baseline scenario is of R_t remaining at 0.752 (black). Vertical dashed lines represent time-points of easing lockdown. (B), (C) The incident and cumulative cases increase in all scenarios in which R_t increases.

Figure 9. Predicted deaths in different scenarios of R_t increase on 1st June, 15th June and 4th July compared with the baseline scenario, and real observed death data from the ONS (light green).

The model compared scenarios in which R_t increases to different values on the 1st, 15th and 4th of July with real observed deaths (light green). The comparator baseline scenario is of R_t remaining at 0.752 (black). Vertical dashed lines represent time-points of easing lockdown. (B), (C) The incident and cumulative deaths increase in all scenarios in which R_t increases. The

For peer review only

daily deaths appear to fit best with the scenarios where R_ts are between 0.85 and 0.95 (dark blue, light blue, and purple) during this period.

Figure 10: Estimated time-varying reproduction number (R_t) for England

The figure shows the R_t estimated from the recent ONS death data (up to September 11, 2020) with 95% credible intervals (grey) with a serial interval of mean 4.7 and SD 2.9 days. We see a gradual upward trend in inferred R_t, with median R_t rising above 1 toward the end of July.

Figure 11: Predicted cases and deaths at different R_t values from current case numbers in England as of 21st September 2020

Figure 11 represents the predicted rise in cases based on different R_t values, and a serial interval of mean 4.7 and SD 2.9 days. The case numbers were calculated as a moving 7 day average from the Public Health England data of confirmed cases within England up to the 21st September. We project case, and death numbers (assuming an IFR of 1%) from these incident case numbers, using different scenarios of R_t. We note that case numbers are likely underestimates, as the testing system within England is currently running at capacity, and not everyone with symptoms is able to access tests.

For peer review only

Figure 1: Estimated time-varying reproduction number (R_t) for England

The figure shows the R_t estimated by Model 3 (blue) with 95% credible intervals (grey) with a serial interval of mean 4.7 and SD 2.9 days. From 3.65 (CI 3.36-3.96), R_t drops on the 16th March and 23rd March (indicated by vertical dashed lines) when social distancing and lockdown were instituted, reaching a low of 0.66 (95% CI 0.34-1.04) in the week of the 30th March. The last estimated R_t is 0.75 (95% CI 0.50-1.00) following the 13th May.

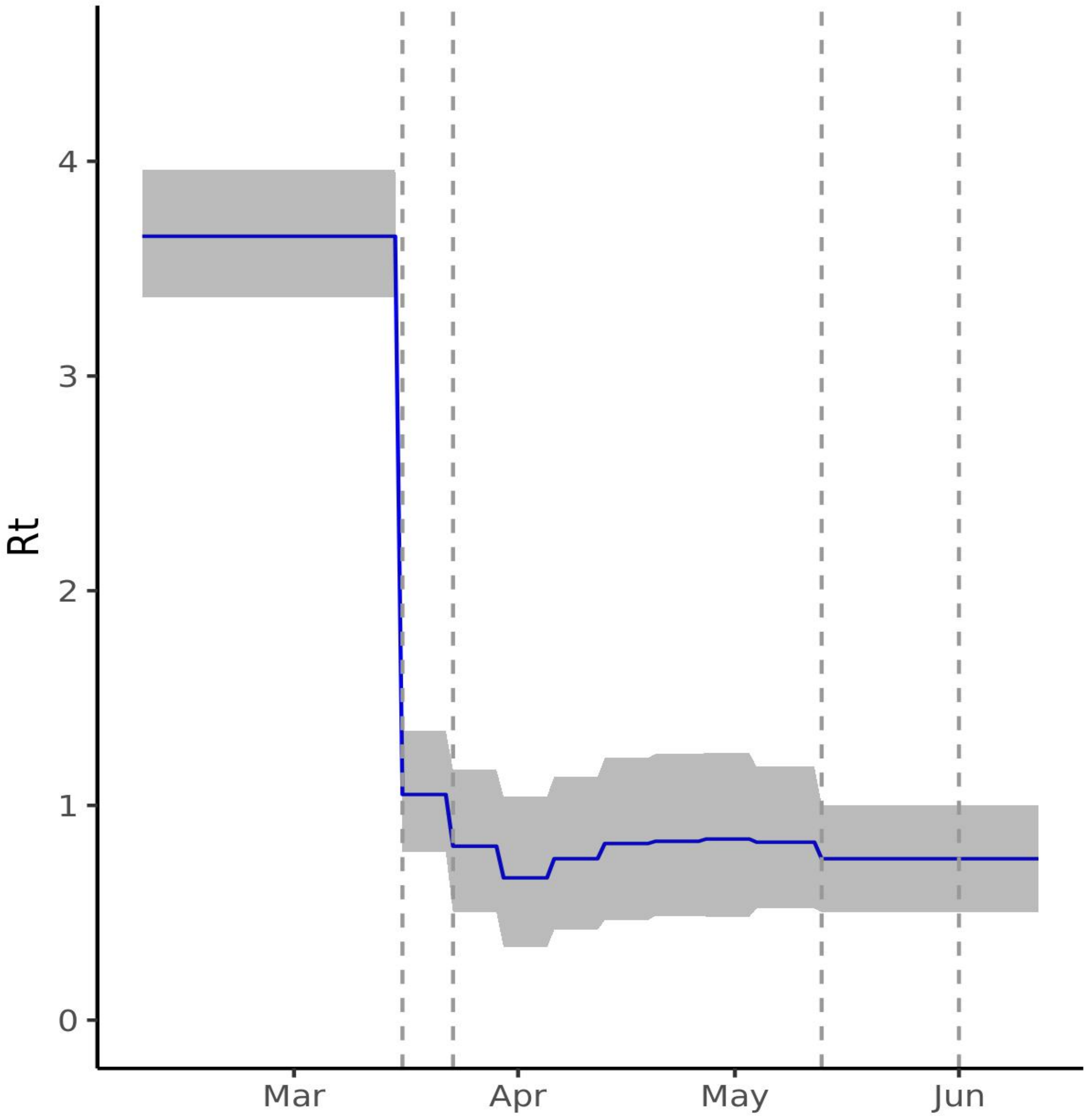

Figure 2: Model fit to observed death data
Daily deaths predicted by Model 3 (blue) with 95% credible intervals (grey) show a good fit to the observed deaths from the ONS (red)

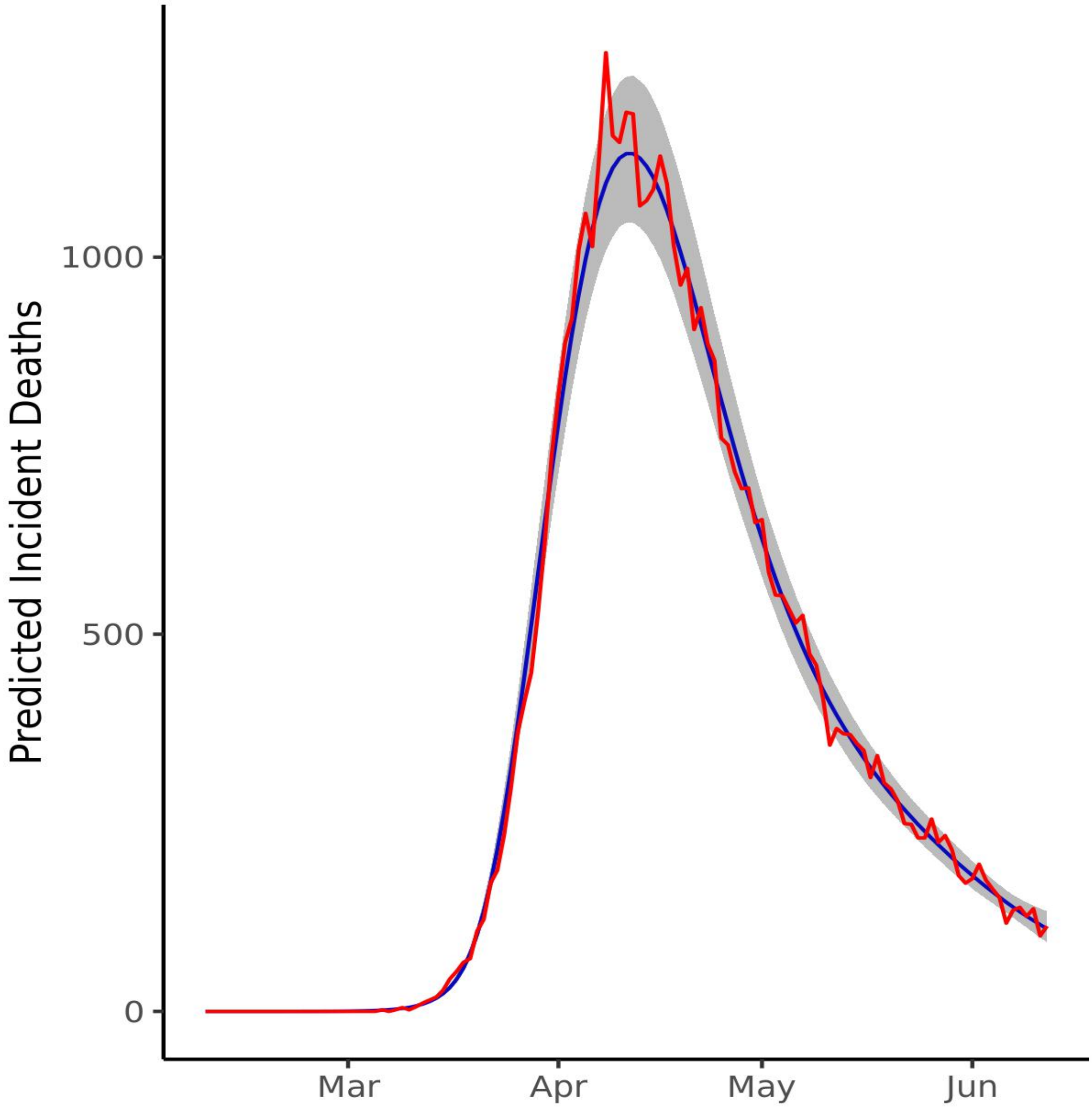

Figure 3. Predicted deaths with R_t increasing on 1st June

(A) The model compared scenarios in which R_t increases to 0.80 (light green), 0.85 (green), 0.90 (dark blue), 0.95 (red), 1 (purple) and 1.05 (brown) and then remains constant for the 90-day forecasting period. The comparator baseline scenario is of R_t remaining at 0.75 (black) and two elimination strategies of R_t reducing to 0.7 (yellow) and 0.6 (light blue) were also considered. Vertical dashed lines represent time-points of easing lockdown. (B), (C) the incident and cumulative deaths increase in all scenarios in which R_t increases and reduces in the two elimination scenarios.

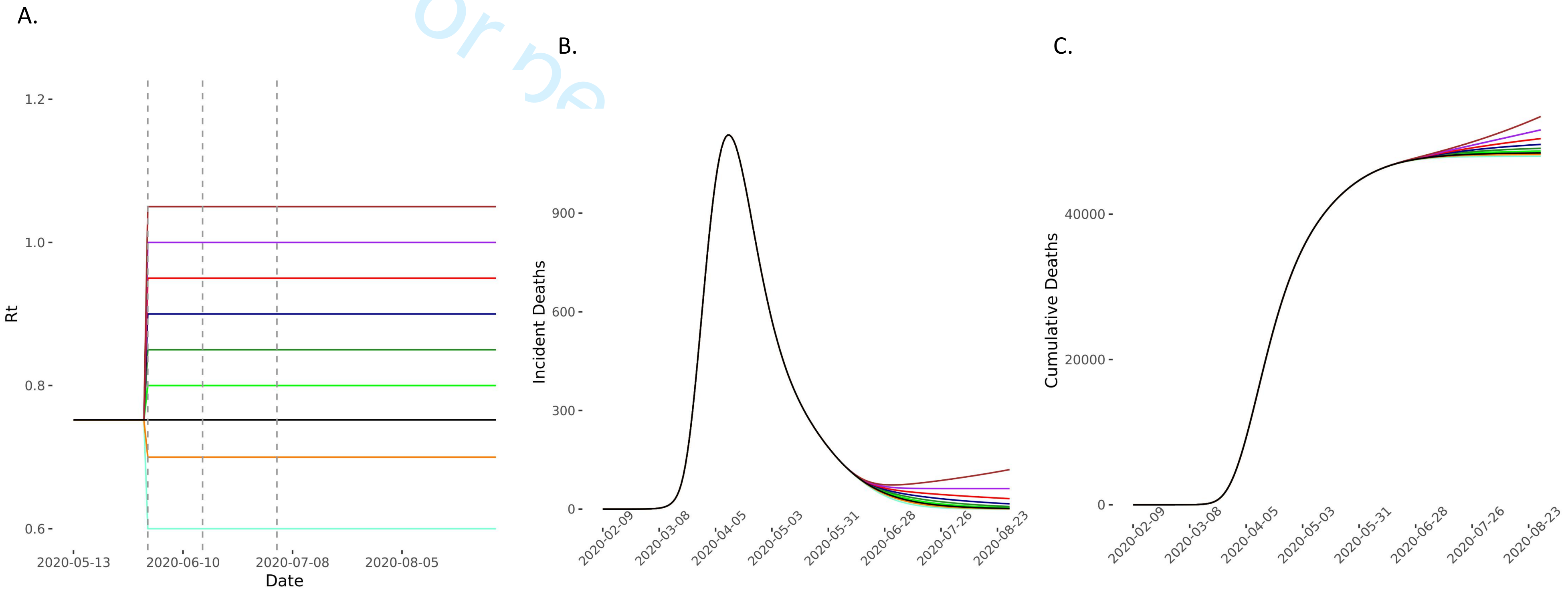

Figure 4. Predicted deaths in scenarios of R_t increase on 1st and 15th June compared with baseline scenario
(A) The model compared scenarios in which R_t increases to 0.80 (light green), 0.85 (green), 0.90 (blue), 0.95 (red), 1 (purple) and 1.05(brown) and then further by 0.05 on the 15th June and then remaining constant for the 90-day forecasting period. The comparator baseline scenario is of R_t remaining at 0.75 (black). Vertical dashed lines represent time-points of easing lockdown. (B), (C) The incident and cumulative deaths increase in all scenarios in which R_t increases.

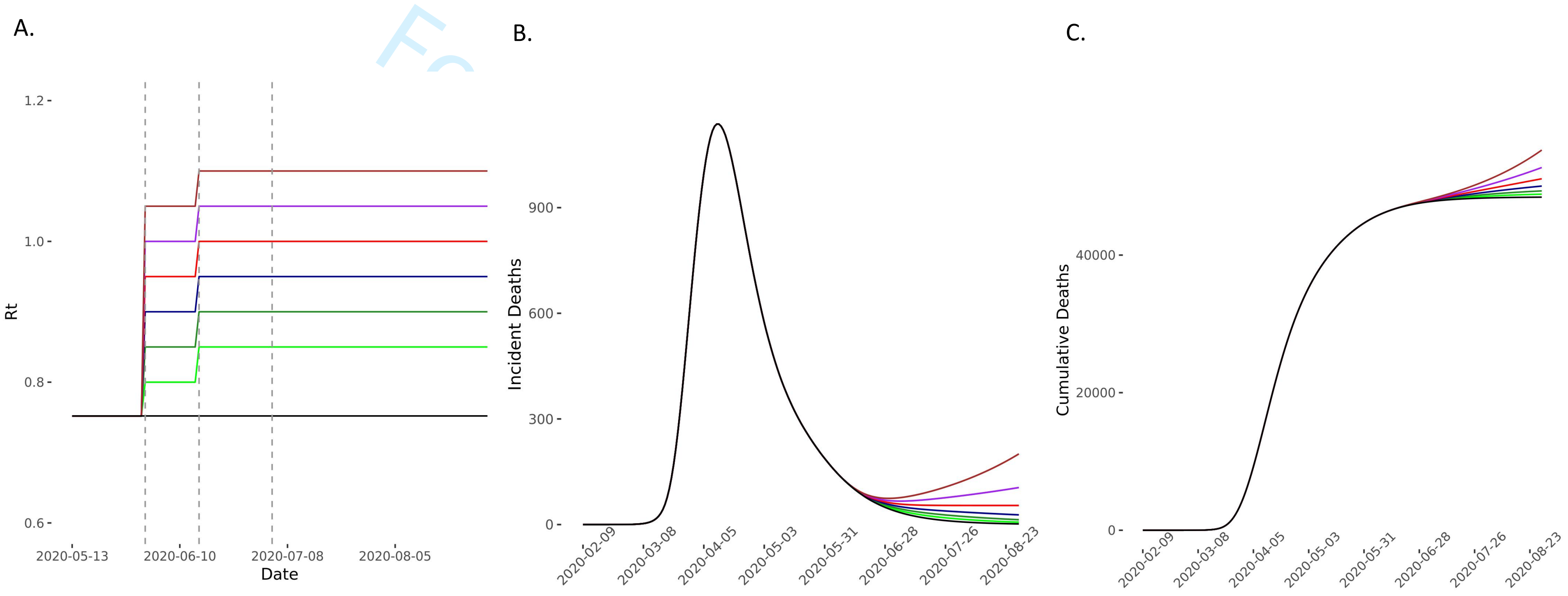

Figure 5. Predicted deaths in scenarios of R_t increase on 1st June, 15th June and 4th July compared with baseline scenario
(A) The model compared scenarios in which R_t increases to 0.80 (light green), 0.85 (green), 0.90 (blue), 0.95 (red), 1 (purple) and 1.05(brown) and then further by 0.05 on the 15th June and then again by 0.05 on the 3rd July before remaining constant for the 90-day forecasting period. The comparator baseline scenario is of R_t remaining at 0.752 (black). Vertical dashed lines represent time-points of easing lockdown. (B), (C) The incident and cumulative deaths increase in all scenarios in which R_t increases.

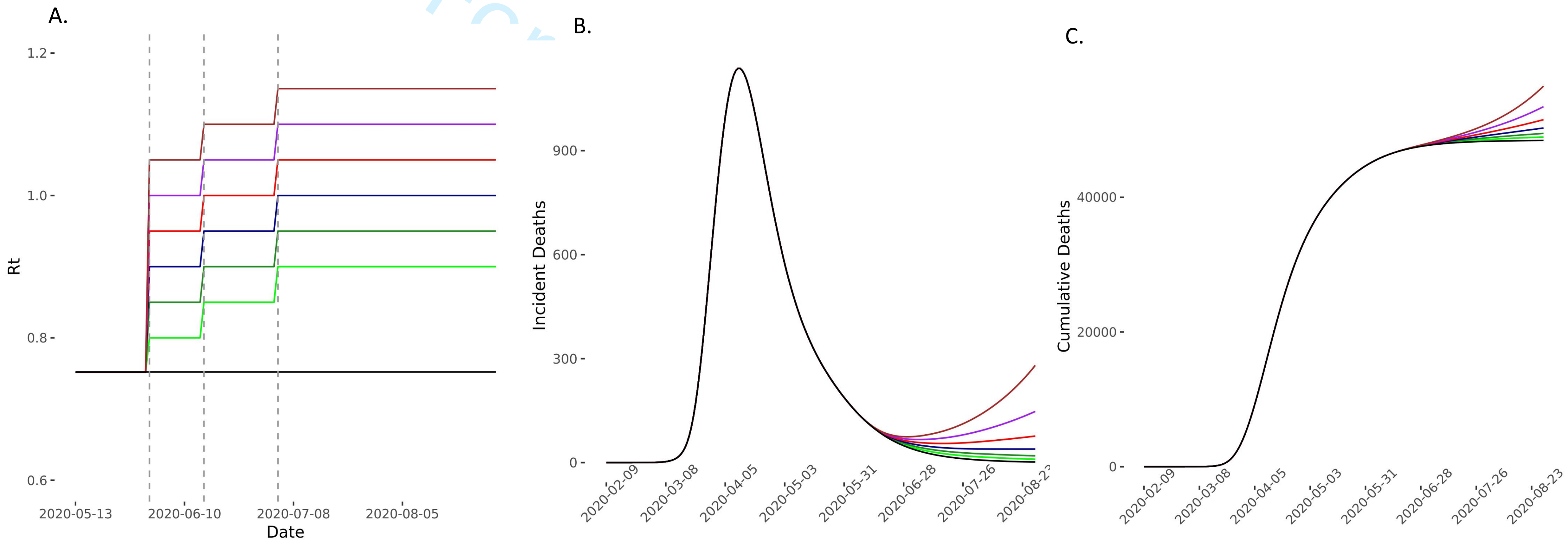

Figure 6. Predicted cases in scenarios of R_t increase on 1st June compared with baseline and elimination scenarios
(A) The model compared scenarios in which R_t increases to 0.80 (light green), 0.85 (green), 0.90 (dark blue), 0.95 (red), 1 (purple) and 1.05(brown) and then remains constant for the 90-day forecasting period. The comparator baseline scenario is of R_t remaining at 0.752 (black) and two elimination strategies of R_t reducing to 0.7 (yellow) and 0.6(light blue) were also considered. Vertical dashed lines represent time-points of easing lockdown. (B), (C) the incident and cumulative cases increase in all scenarios in which R_t increases and reduces in the two elimination scenarios.

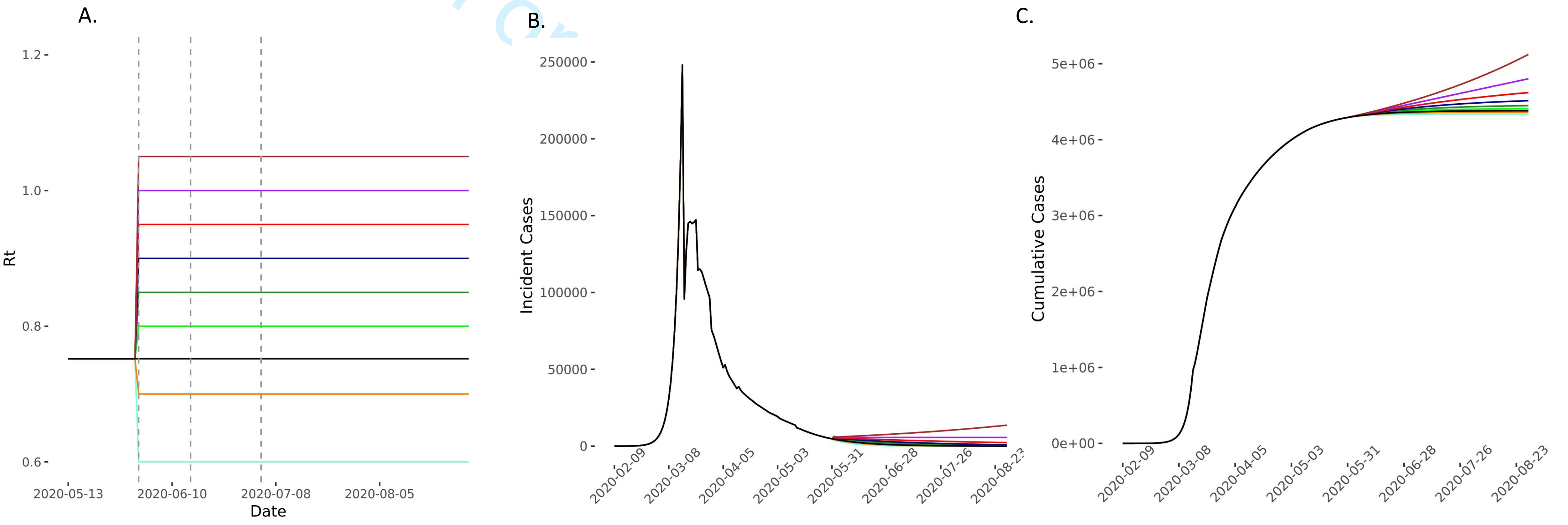

For peer review only - http://bmjopen.bmj.com/site/about/guidelines.xhtml

Figure 7. Predicted cases in scenarios of R_t increase on 1st June and 15th June compared with the baseline scenario

(A) The model compared scenarios in which R_t increases to 0.80 (light green), 0.85 (green), 0.90 (blue), 0.95 (red), 1 (purple) and 1.05(brown) and then further by 0.05 on the 15th June and then remaining constant for the 90-day forecasting period. The comparator baseline scenario is of R_t remaining at 0.752 (black). Vertical dashed lines represent time-points of easing lockdown. (B), (C) The incident and cases increase in all scenarios in which R_t increases.

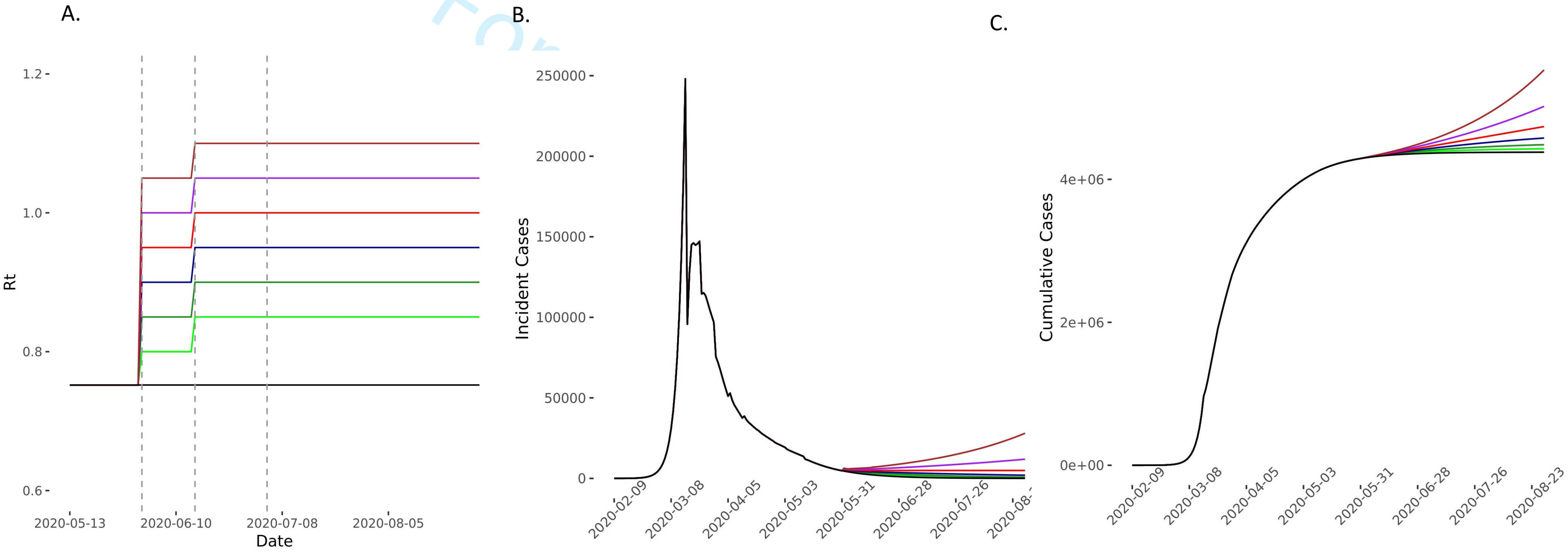

Figure 8. Predicted cases in scenarios of R_t increase on 1st June and 15th June and 4th July compared with the baseline scenario
(A) The model compared scenarios in which R_t increases to 0.80 (light green), 0.85 (green), 0.90 (blue), 0.95 (red), 1 (purple) and 1.05(brown) and then further by 0.05 on the 15th June and then again by 0.05 on the 3rd July before remaining constant for the 90-day forecasting period. The comparator baseline scenario is of R_t remaining at 0.752 (black). Vertical dashed lines represent time-points of easing lockdown. (B), (C) The incident and cumulative cases increase in all scenarios in which R_t increases.

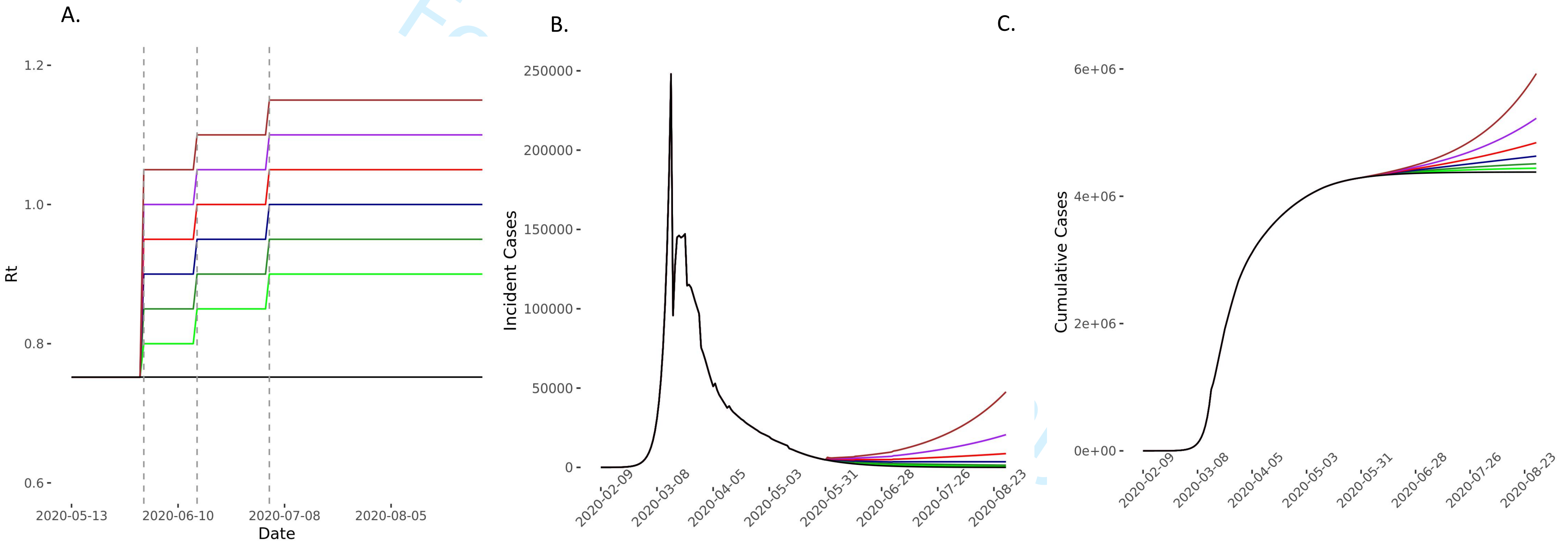

Figure 9. Predicted deaths in different scenarios of R_t increase on 1st June, 15th June and 4th July compared with the baseline scenario, and real observed death data from the ONS (light green).

The model compared scenarios in which R_t increases to different values on the 1st, 15th and 4th of July with real observed deaths (light green). The comparator baseline scenario is of R_t remaining at 0.752 (black). Vertical dashed lines represent time-points of easing lockdown. (B), (C) The incident and and cumulative deaths increase in all scenarios in which R_t increases. The daily deaths appear to fit best with the scenarios where R_ts were between 0.85 and 0.95 (dark blue, light blue, and purple) during this period.

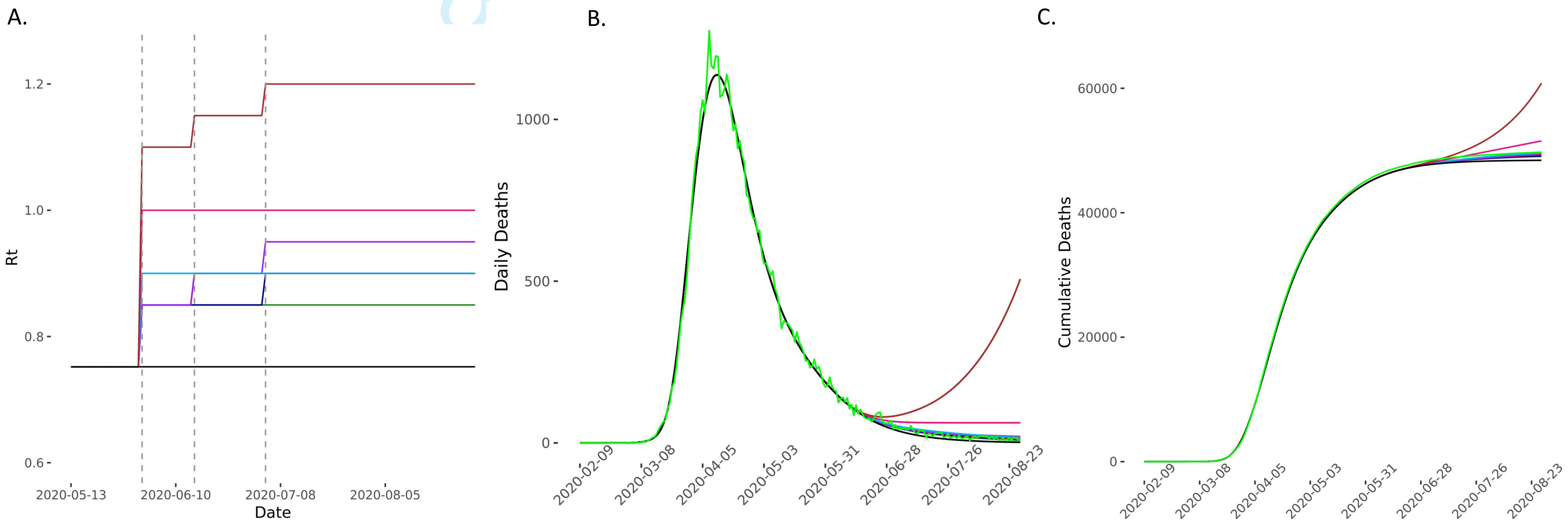

Figure 10: Estimated time-varying reproduction number (R_t) for England
The figure shows the R_t estimated from the recent ONS death data (up to September 11, 2020) with 95% credible intervals (grey) with a serial interval of mean 4.7 and SD 2.9 days. We see a gradual upward trend in R_t, with median R_t rising above 1 toward the end of July.

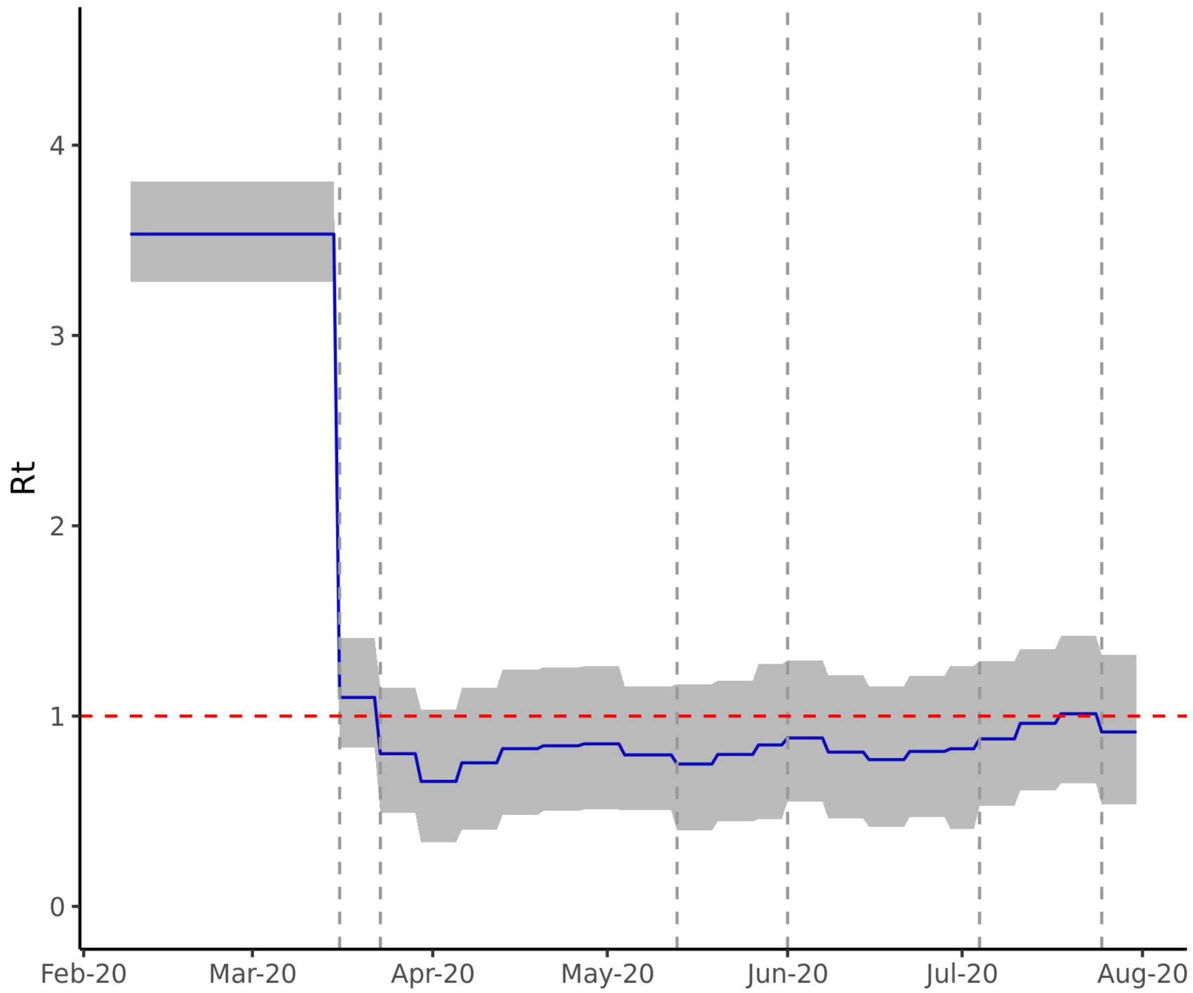

Figure 11: Predicted cases and deaths at different R_t values from current case numbers in England as of 21st September 2020

Figure 11 represents the predicted rise in cases based on different R_t values, and a serial interval of mean 4.7 and SD 2.9 days. The case numbers were calculated as a moving 7 day average from the Public Health England data of confirmed cases within England up to the 21st September. We project case, and death numbers (assuming an IFR of 1%) from these incident case numbers, using different scenarios of R_t. We note that case numbers are likely underestimates, as the testing system within England is currently running at capacity, and not everyone with symptoms is able to access tests.

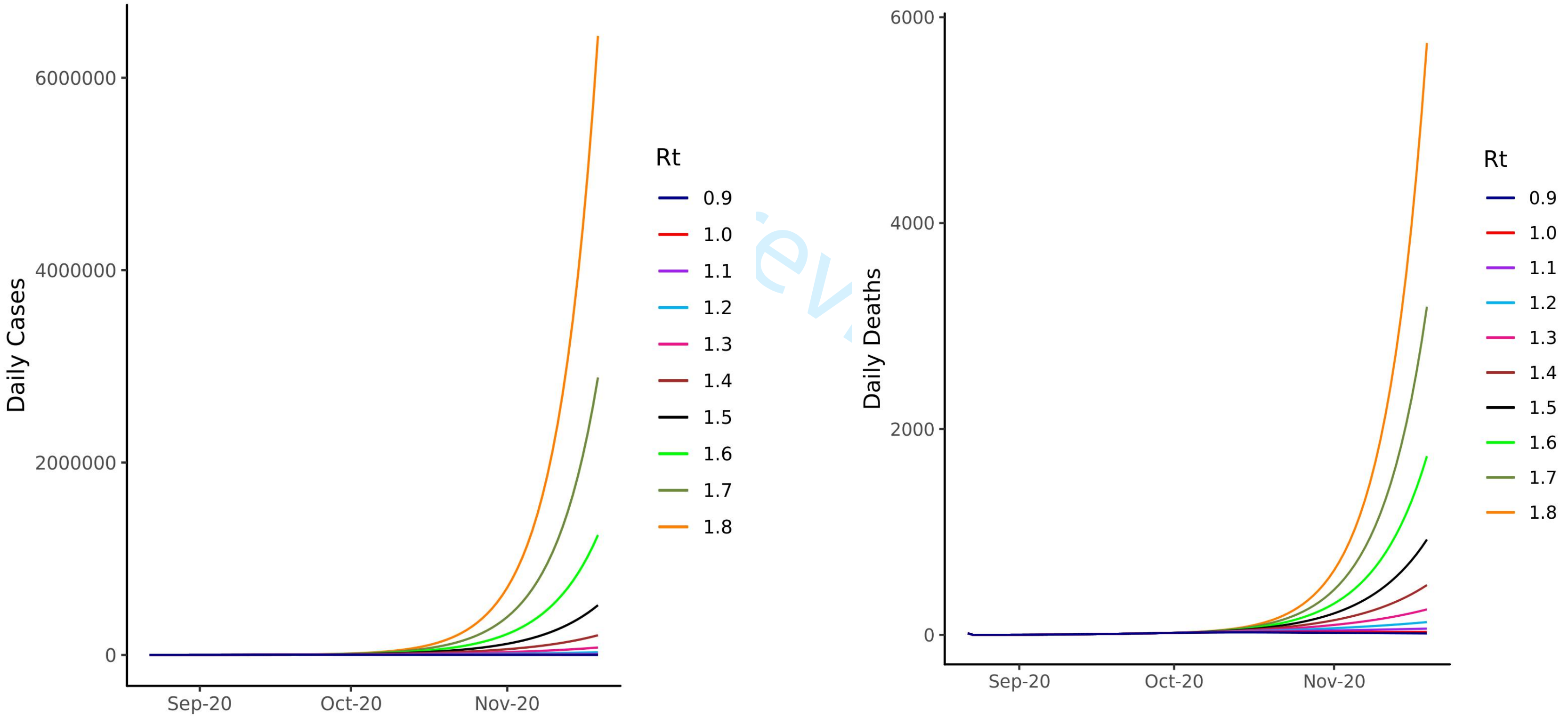

SUPPLEMENTARY MATERIALS

Modelling the impact of lockdown easing measures on cumulative COVID-19 cases and deaths in England

Ziauddeen H, PhD [1,2,3], Subramaniam N, BSc [1,2], Gurdasani D, PhD† [4].

Supplementary Table 1: Comparison of Bayesian models with different constraints on changes in R_t

Model	RMSE	EPLD	SE	diff_EPLD_model3	diff_SE_model3
model 1	38.0	-505.1	25.0	-1.5	0.5
model 2	28.3	-504.8	24.5	-1.2	0.9
model 3	28.0	-503.6	24.8	NA	NA

Supp. Table 1 represents model comparisons between Models 1-3, as specified in the text. RMSE represents the Root mean squared error between estimated and observed deaths for each model. EPLD represents the expected log pointwise predictive density which approximates leave-one-out (LOO) cross-validation. Less negative scores suggest better fit. SE is the standard error of EPLD. We assess the difference in EPLD between all models and the best performing model (Model 3 in this case), comparing the difference in EPLD (diff_EPLD_model3) with the standard error of the difference (diff_SE_model3). Although all three models appear comparable in performance, Model 3 appears to show the best fit with the lowest RMSE, and the least negative EPLD.

For peer review only

Supplementary Table 2: Comparison of models excluding specific change points for R_t

change points removed	RMSE	EPLD	SE	diff_EPLD	diff_SE
16th March	118.1	-576.0	26.1	-70.9	5.3
23rd March	44.6	-506.7	25.2	-1.6	1.0
13th May	40.2	-504.8	25.0	0.3	0.4
1st June	38.1	-505.2	25.0	-0.1	0.0
None (all included)	38.0	-505.1	25.0	NA	NA

Supp. Table 2 represents model comparisons between Models that constrain Rt at each of the 4 hypothesised change points at which point social distancing or lockdown measures were introduced (16th March and 23rd March), or when lockdown measures were eased (13th May and 1st June). The first column represents the change point left out in each model, with the last model with all three change points being the comparator, as specified in the text. RMSE represents the Root mean squared error between estimated and observed deaths for each model. EPLD represents the expected log pointwise predictive density which approximates leave-one-out (LOO) cross-validation. Less negative scores suggest better fit. SE is the standard error of EPLD. We assess the difference in EPLD between all models and the model with all three change points, comparing the difference in EPLD (diff_EPLD) with the standard error of the difference (diff_SE). The model leaving out 16th March as a change point, i.e. constraining Rt to remain constant at this point appears to adversely impact fit the most.

For peer review only

Supplementary Table 3: Cumulative cases and deaths in lockdown easing scenarios in primary model

Rt 1st June	Rt 15th June	Rt 4th July	Cumulative cases	Cumulative deaths	Cases difference from baseline	Death difference from baseline	RMSE
0.752	0.752	0.752	4411594(4199223-4639250)	48501(46170-50989)	0(0,0)	0(0,0)	
0.6	0.6	0.6	4364386(4162299-4580834)	48006(45783-50386)	-44302(-84684--18600	-462(-884--194)	25.3
0.65	0.65	0.65	4374559(4170697-4593499)	48115(45875-50523)	-33831(-64668--14204)	-350(-669--147)	24.9
0.7	0.7	0.7	4391027(4183302-4610584)	48286(46007-50696)	-19968(-38168--8384)	-204(-389--86)	24.4
0.75	0.75	0.75	4410590(4198499-4637531)	48494(46163-50977)	-908(-1736--381)	-9(-17--4)	23.9
0.75	0.75	0.8	4415149(4201945-4645342)	48518(46186-51016)	3069(1285-5890)	19(8-37)	23.8
0.75	0.8	0.8	4424153(4209052-4658126)	48612(46255-51149)	11497(4814-22058)	102(43-195)	23.7
0.75	0.8	0.85	4430866(4213721-4668154)	48654(46293-51225)	18197(7620-34906)	145(61-278)	23.6
0.8	0.8	0.8	4439684(4219884-4679358)	48771(46375-51380)	26447(11105-50549)	257(108-492)	23.4
0.8	0.8	0.85	4447876(4225283-4692920)	48827(46413-51458)	34303(14397-65598)	308(129-589)	23.3
0.8	0.85	0.85	4461240(4232489-4716984)	48954(46492-51680)	47523(19934-90933)	431(181-825)	23.2
0.8	0.85	0.9	4474630(4243279-4736698)	49036(46538-51811)	60851(25519-116475)	508(213-972)	23.1
0.85	0.85	0.85	4481739(4247839-4745973)	49166(46614-52010)	67576(28376-129149)	632(265-1208)	23.0
0.85	0.85	0.9	4498639(4257710-4770487)	49246(46692-52138)	83109(34888-158891)	722(303-1379)	23.0
0.85	0.9	0.9	4521199(4273493-4806484)	49446(46808-52428)	104334(43782-199536)	907(381-1733)	23.1
0.85	0.9	0.95	4547931(4291851-4848863)	49592(46917-52640)	130823(54887-250256)	1043(438-1994)	23.2
0.9	0.9	0.9	4549190(4292969-4850945)	49730(47007-52865)	132381(55595-252977)	1173(493-2240)	23.3
0.9	0.9	0.95	4579138(4311424-4905124)	49887(47120-53118)	163082(68471-311726)	1330(559-2542)	23.5
0.9	0.95	0.95	4613802(4328984-4965451)	50162(47308-53602)	197988(83107-378532)	1610(676-3077)	24.3
0.9	0.95	1	4667450(4358502-5053567)	50397(47439-54007)	250499(105129-479023)	1848(776-3534)	25.2
0.95	0.95	0.95	4655308(4352263-5032669)	50517(47499-54226)	239051(100411-456749)	1971(828-3764)	25.3
0.95	0.95	1	4718132(4385399-5139609)	50790(47648-54680)	299596(125815-572543)	2246(943-4290)	26.6
0.95	1	1	4779022(4412370-5246023)	51226(47883-55381)	358354(150465-684935)	2672(1122-5106)	28.8
0.95	1	1.05	4880364(4464544-5437916)	51650(48103-56116)	462002(193954-883178)	3087(1296-5899)	31.8
1	1	1	4840595(4444280-5359379)	51743(48160-56290)	421310(177012-804811)	3174(1334-6060)	31.4
1	1	1.05	4959206(4498273-5586656)	52235(48368-57131)	540234(226938-1032145)	3649(1533-6970)	35.2
1	1.05	1.05	5055758(4548961-5767421)	52892(48712-58297)	641220(269327-1225202)	4303(1808-8220)	40.2
1	1.05	1.1	5264669(4632979-6143068)	53598(49059-59632)	844596(354706-1613979)	5018(2108-9587)	47.2
1.05	1.05	1.05	5156984(4594880-5946919)	53594(49059-59623)	741957(311832-1416940)	5017(2109-9578)	45.3
1.05	1.05	1.1	5397044(4692140-6391841)	54411(49421-61165)	974252(409410-1860772)	5833(2452-11138)	53.5
1.05	1.1	1.1	5574165(4770085-6732948)	55401(49905-63038)	1150799(483559-2198106)	6843(2876-13067)	62.8
1.05	1.1	1.15	5969381(4946974-7481001)	56609(50491-65190)	1546934(649955-2954983)	8065(3390-15402)	76.7
1.1	1.1	1.1	5744209(4843263-7044331)	56428(50400-64839)	1317940(554129-2516086)	7878(3313-15037)	71.5
1.1	1.1	1.15	6189495(5035202-7885232)	57860(50954-67461)	1768512(743504-3376542)	9269(3898-17692)	87.4
1.1	1.15	1.15	6501607(5163364-8475727)	59458(51650-70470)	2081127(874883-3973567)	10834(4556-20682)	103.2
1.1	1.15	1.2	7272289(5484637-9955130)	61543(52551-74465)	2846203(1196439-5434639)	12908(5427-24642)	128.7

Supplementary Table 4 represents the estimated cumulative deaths, cumulative cases, and excess deaths and cases in different scenarios of changing Rt at points of easing lockdown in comparison with the baseline scenario of Rt remaining constant at 0.752.

For peer review only

Supplementary Table 4: Cumulative cases and deaths in lockdown easing scenarios in model with long serial interval

Rt 1st June	Rt 15th June	Rt 4th July	Cumulative cases	Cumulative deaths	Cases difference from baseline	Death difference from baseline
0.691	0.691	0.691	4404236(4183512-4622330)	48411(45990-50805)	0(0-0)	0(0-0)
0.6	0.6	0.6	4371866(4158398-4583033)	48078(45733-50400)	-32402(-52309--17783)	-330(-533--181)
0.65	0.65	0.65	4387428(4171118-4603369)	48241(45863-50609)	-16515(-26661--9065)	-166(-268--91)
0.7	0.7	0.7	4408635(4186726-4627236)	48451(46022-50849)	4158(2283-6712)	41(23-66)
0.7	0.7	0.75	4413466(4190714-4632818)	48487(46047-50893)	8904(4865-14430)	75(41-121)
0.7	0.75	0.75	4421929(4197326-4644215)	48571(46126-50997)	17612(9599-28584)	160(87-259)
0.7	0.75	0.8	4428891(4202474-4652651)	48618(46159-51054)	24664(13432-40046)	206(112-334)
0.75	0.75	0.75	4436254(4207602-4661170)	48717(46226-51175)	31769(17441-51275)	307(168-494)
0.75	0.75	0.8	4444061(4214414-4673025)	48768(46266-51240)	39710(21767-64168)	358(197-579)
0.75	0.8	0.8	4456057(4225564-4689908)	48884(46358-51403)	51923(28426-84007)	473(259-765)
0.75	0.8	0.85	4468509(4233843-4705662)	48955(46423-51508)	63758(34875-103225)	544(298-881)
0.8	0.8	0.8	4474042(4238163-4713852)	49060(46515-51637)	69645(38242-112391)	652(358-1051)
0.8	0.8	0.85	4487239(4247137-4731986)	49147(46570-51756)	82925(45486-133921)	732(402-1180)
0.8	0.85	0.85	4504368(4260726-4758965)	49297(46674-51983)	100288(54965-162081)	887(487-1433)
0.8	0.85	0.9	4524968(4276399-4786847)	49405(46756-52128)	120111(65789-194244)	997(546-1610)
0.85	0.85	0.85	4528097(4278796-4789875)	49515(46843-52296)	122997(67554-198451)	1107(609-1785)
0.85	0.85	0.9	4550519(4295317-4824987)	49639(46934-52475)	145167(79670-234360)	1229(675-1983)
0.85	0.9	0.9	4575600(4314157-4857930)	49864(47104-52757)	170201(93348-274900)	1441(791-2326)
0.85	0.9	0.95	4608999(4336767-4903946)	50032(47229-53038)	203356(111471-328606)	1608(883-2597)
0.9	0.9	0.9	4605943(4334310-4898052)	50138(47312-53190)	200055(109912-322694)	1716(944-2766)
0.9	0.9	0.95	4643792(4360620-4952873)	50326(47454-53431)	237006(130136-382485)	1902(1046-3067)
0.9	0.95	0.95	4680570(4385671-5007504)	50621(47645-53806)	273569(150145-441642)	2192(1204-3535)
0.9	0.95	1	4735012(4422134-5091796)	50870(47843-54169)	328896(180416-531139)	2446(1343-3946)
0.95	0.95	0.95	4720210(4412771-5069359)	50965(47911-54323)	313876(172518-506126)	2540(1397-4093)
0.95	0.95	1	4781115(4451857-5160478)	51266(48080-54750)	375333(206196-605461)	2822(1552-4549)
0.95	1	1	4836432(4486836-5241471)	51661(48368-55340)	429433(235844-692907)	3219(1770-5191)
0.95	1	1.05	4928929(4549055-5385960)	52032(48613-55941)	521422(286255-841597)	3603(1980-5811)
1	1	1	4892268(4526735-5326989)	52097(48655-56020)	485265(266854-782184)	3667(2018-5906)
1	1	1.05	4995150(4589022-5491882)	52523(48926-56643)	587186(322775-946757)	4092(2252-6593)
1	1.05	1.05	5076092(4640904-5614490)	53078(49274-57480)	668188(367218-1077551)	4639(2552-7476)
1	1.05	1.1	5227156(4734604-5849954)	53653(49678-58359)	820638(450864-1323717)	5217(2869-8409)
1.05	1.05	1.05	5154698(4689409-5733251)	53657(49684-58359)	747364(411252-1204170)	5221(2876-8408)
1.05	1.05	1.1	5321327(4792479-5997797)	54309(50099-59370)	915842(503787-1475921)	5860(3227-9437)
1.05	1.1	1.1	5444111(4858418-6188703)	55069(50551-60585)	1038372(571090-1673570)	6615(3642-10657)
1.05	1.1	1.15	5698707(4995152-6588038)	55936(51099-61889)	1290002(709312-2079494)	7480(4117-12052)
1.1	1.1	1.1	5558967(4921706-6371716)	55831(51060-61736)	1153037(634971-1857036)	7382(4069-11884)
1.1	1.1	1.15	5838364(5075136-6811946)	56779(51632-63173)	1430388(787494-2304058)	8336(4593-13420)
1.1	1.15	1.15	6021434(5176394-7116504)	57812(52276-64824)	1617286(890276-2605292)	9384(5170-15109)
1.1	1.15	1.2	6430941(5404442-7783304)	59109(53001-66839)	2030763(1117659-3271715)	10673(5879-17187)

Supplementary Table 5 represents the estimated cumulative deaths, cumulative cases, and excess deaths and cases in different scenarios of changing Rt at points of easing lockdown in comparison with the baseline scenario of Rt remaining constant at 0.691.

For peer review only

Supplementary Figure 1: Distribution of R-hat for parameters from final model

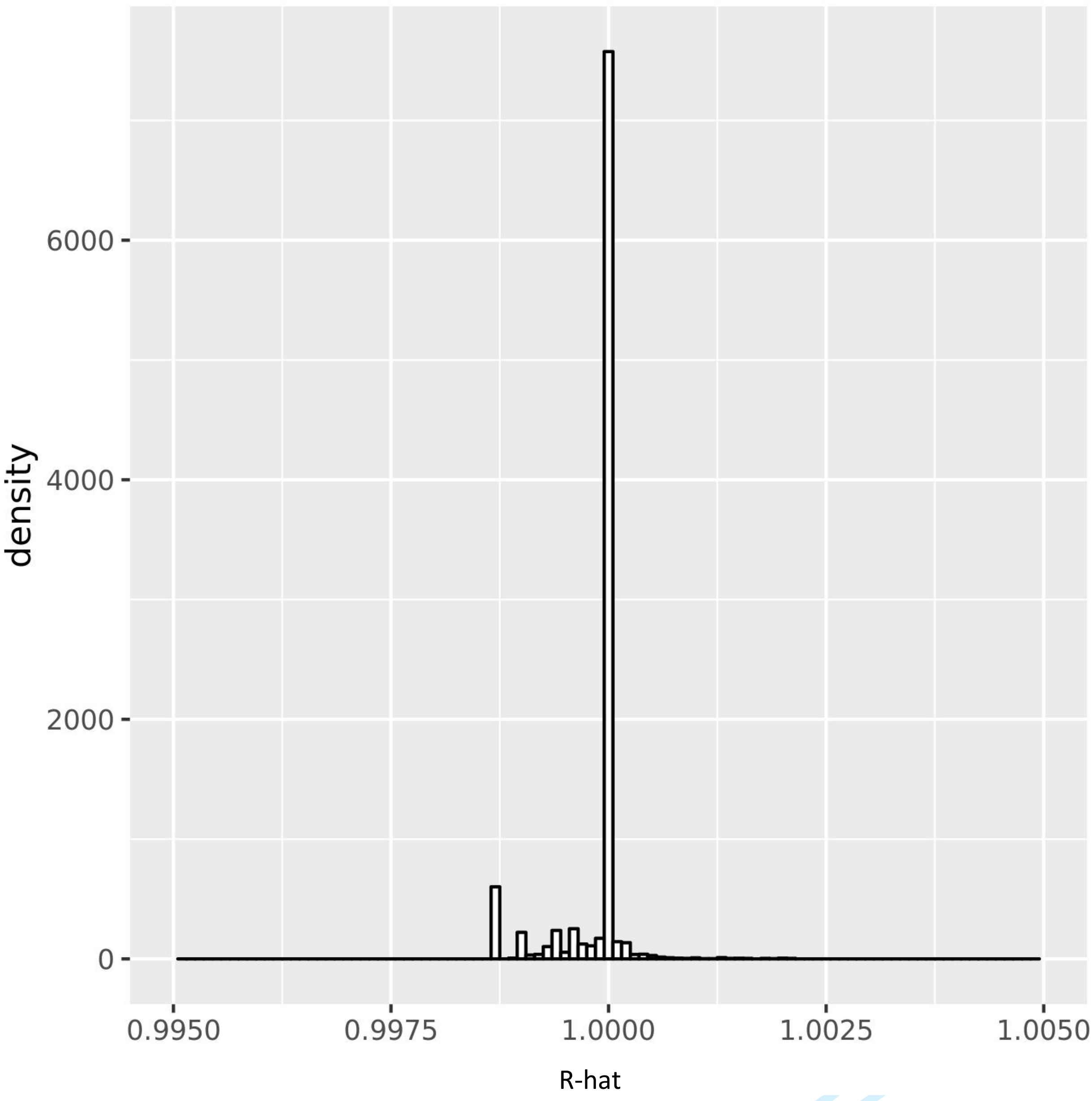

Supplementary Figure 1 represents the estimated R-hat for parameters of the final model with duplicates removed. The mean R-hat was 1.000057. An R-hat near 1 suggests that between-chain variance for a given parameter is equal to the within-chain variance, suggesting convergence of the model. All values were well below 1.05.

Supplementary Figure 2: Pareto shape parameter k distribution for final model

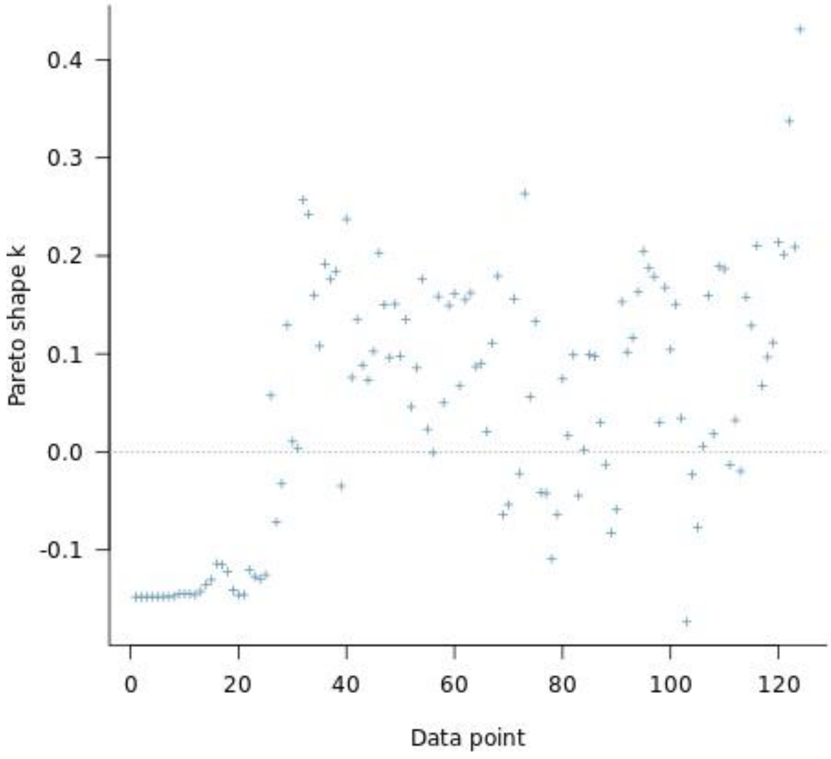

The estimated shape parameter k of the generalized Pareto distribution can be used to assess the reliability of the estimate from approximations of Leave-one-out cross-validation (LOO). The k shape values are all below 0.5, suggesting our estimates are reliable.

eview only

Supplementary Figure 3: Rt estimates with broad and uninformative priors

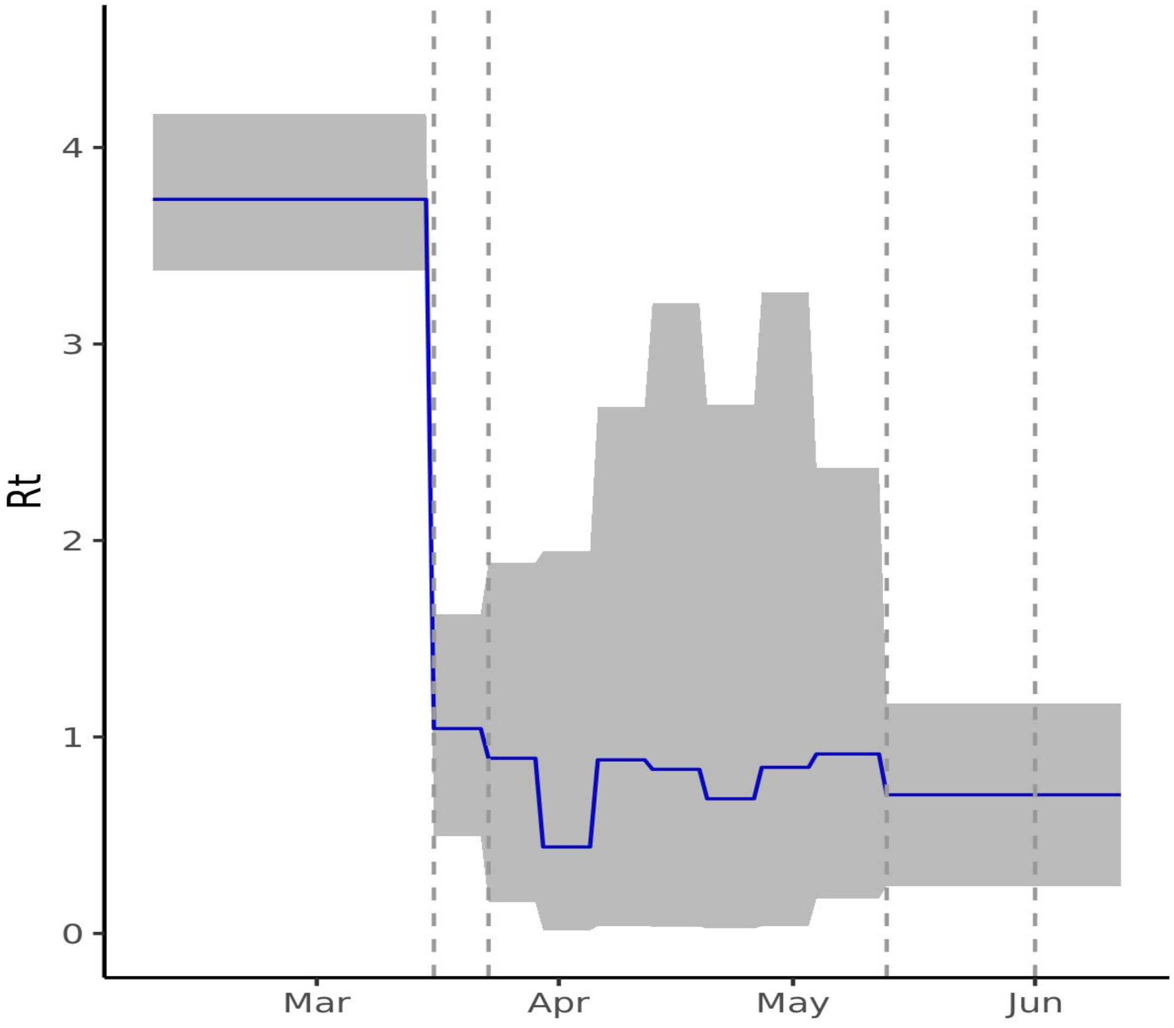

Supplementary Figure 3. Represents estimates of Rt when uninformative priors are used for estimation. We find that although uncertainty is greater around estimates, median estimates, and patterns of changes are similar as for the original model for all time intervals, suggesting that these are not constrained by specification of the prior in the final model.

Supplementary Figure 4: Estimated reproduction number in model with longer serial interval

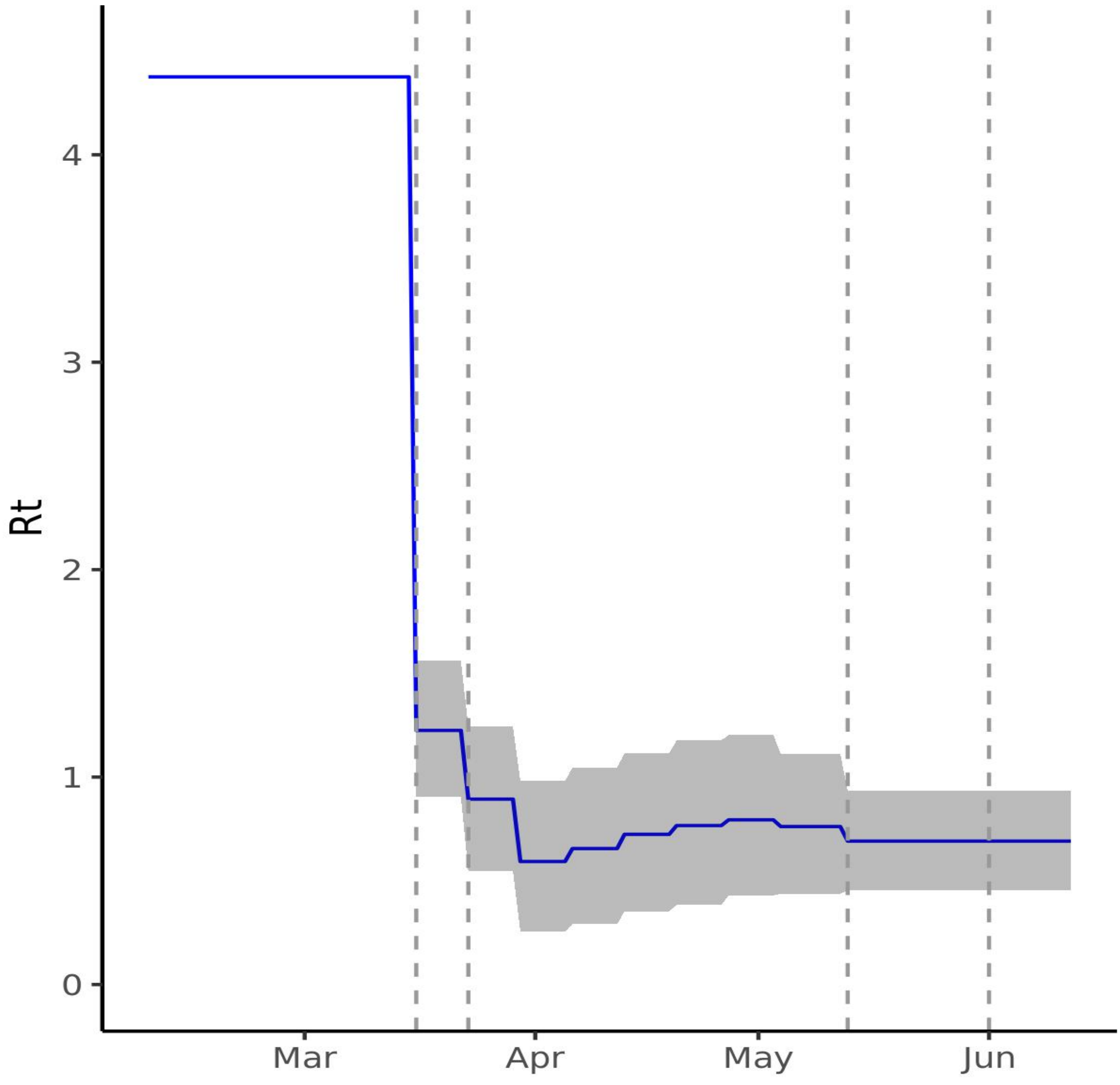

The figure shows the R_t estimated by a model with a a serial interval of mean 6.5 and coefficient of variation of 0.72. While estimates of R0 are higher in this model, estimates during other time intervals following lockdown are very similar to our primary model. 95% credible intervals are represented by grey bands.

Supplementary Figure 5: Predicted and observed deaths in model with longer serial interval

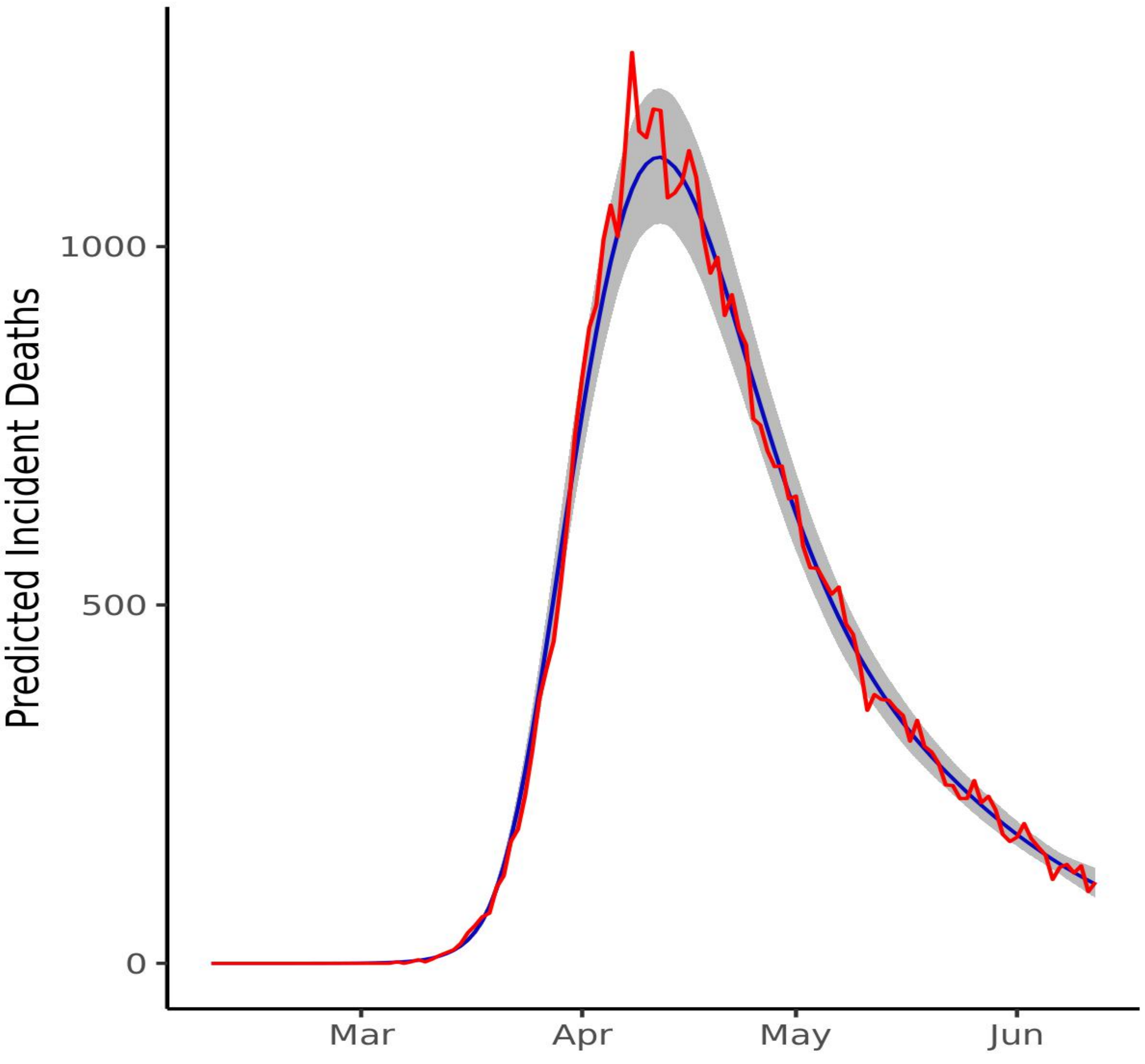

Daily deaths predicted by a model specifying longer serial intervals (blue) with 95% credible intervals (grey) show a good fit to the observed deaths from the ONS (red)

BMJ Open

Modelling the impact of lockdown easing measures on cumulative COVID-19 cases and deaths in England

Journal:	*BMJ Open*
Manuscript ID	bmjopen-2020-042483.R2
Article Type:	Original research
Date Submitted by the Author:	15-Jun-2021
Complete List of Authors:	Ziauddeen, Hisham; University of Cambridge<br>Subramaniam, Naresh; University of Cambridge<br>Gurdasani, Deepti; Queen Mary University of London, William Harvey Research Institute
**Primary Subject Heading**:	Epidemiology
Secondary Subject Heading:	Epidemiology
Keywords:	EPIDEMIOLOGY, Health policy < HEALTH SERVICES ADMINISTRATION & MANAGEMENT, Public health < INFECTIOUS DISEASES, Infection control < INFECTIOUS DISEASES

SCHOLARONE™
Manuscripts

I, the Submitting Author has the right to grant and does grant on behalf of all authors of the Work (as defined in the below author licence), an exclusive licence and/or a non-exclusive licence for contributions from authors who are: i) UK Crown employees; ii) where BMJ has agreed a CC-BY licence shall apply, and/or iii) in accordance with the terms applicable for US Federal Government officers or employees acting as part of their official duties; on a worldwide, perpetual, irrevocable, royalty-free basis to BMJ Publishing Group Ltd ("BMJ") its licensees and where the relevant Journal is co-owned by BMJ to the co-owners of the Journal, to publish the Work in this journal and any other BMJ products and to exploit all rights, as set out in our licence.

The Submitting Author accepts and understands that any supply made under these terms is made by BMJ to the Submitting Author unless you are acting as an employee on behalf of your employer or a postgraduate student of an affiliated institution which is paying any applicable article publishing charge ("APC") for Open Access articles. Where the Submitting Author wishes to make the Work available on an Open Access basis (and intends to pay the relevant APC), the terms of reuse of such Open Access shall be governed by a Creative Commons licence – details of these licences and which Creative Commons licence will apply to this Work are set out in our licence referred to above.

Other than as permitted in any relevant BMJ Author's Self Archiving Policies, I confirm this Work has not been accepted for publication elsewhere, is not being considered for publication elsewhere and does not duplicate material already published. I confirm all authors consent to publication of this Work and authorise the granting of this licence.

For peer review only

Modelling the impact of lockdown easing measures on cumulative COVID-19 cases and deaths in England

Ziauddeen H, PhD [1,2,3], Subramaniam N, BSc [1,2], Gurdasani D, PhD† [4].

[1] Dept. of Psychiatry, University of Cambridge, Cambridge UK

[2] Wellcome Trust-MRC Institute of Metabolic Science, University of Cambridge, Cambridge UK

[3] Cambridgeshire & Peterborough Foundation Trust, Cambridge UK

[4] William Harvey Research Institute, Barts and The London School of Medicine and Dentistry, Queen Mary University of London, London UK

† **Corresponding author**:

Dr Deepti Gurdasani
William Harvey Research Institute,
Barts and The London School of Medicine and Dentistry,
Queen Mary University of London, London UK
Email: d.gurdasani@qmul.ac.uk
Phone: 07737650357

For peer review only

Abstract:

Objectives:
To assess the potential impacts of successive lockdown easing measures in England, at a point in the COVID-19 pandemic when community transmission levels were relatively high.

Design:
We developed a Bayesian model to infer incident cases and *R* in England, from incident death data. We then used this to forecast excess cases and deaths in multiple plausible scenarios in which *R* increases at one or more time points.

Setting:
England

Participants:
Publicly available national incident death data for COVID-19 were examined.

Primary Outcome:
Excess cumulative cases and deaths forecast at 90 days, in simulated scenarios of plausible increases in *R* after successive easing of lockdown in England, compared to a baseline scenario where *R* remained constant.

Results:
Our model inferred an *R* of 0.75 on the 13th May when England first started easing lockdown. In the most conservative scenario modelled where *R* increased to 0.80 as lockdown was eased further on 1st June and then remained constant, the model predicted an excess 257 (95% 108-492) deaths and 26,447 (95% CI 11,105-50,549) cumulative cases over 90 days. In the scenario with maximal increases in *R* (but staying ≤1), the model predicts 3,174 (95% CI 1,334-6,060) excess cumulative deaths and 421,310 (95% CI 177,012-804,811) cases. Observed data from the forecasting period aligned most closely to the scenario in which *R* increased to 0.85 on the 1st June, and 0.9 on the 4th July.

Conclusions:
When levels of transmission are high, even small changes in *R* with easing of lockdown can have significant impacts on expected cases and deaths, even if *R* remains ≤1. This will have a major impact on population health, tracing systems and health care services in England. Following an elimination strategy rather than one of maintenance of *R*≤1 would substantially mitigate the impact of the COVID-19 epidemic within England.

Strengths and limitations

1. This study provides urgently needed information about the potential impact of successive lockdown easing measures in England when community transmission of SARS-CoV2 is relatively high.
2. We utilise a robust Bayesian model based on ONS registered deaths in England, to infer incident cases and reproduction number and then forecast deaths and cases

For peer review only

considering multiple plausible scenarios of increase in reproduction number with successive easing of lockdown in England.

3. Our study focuses on the impact of easing lockdown in the conservative scenario when R is maintained at or below 1 in line with stated government policy, showing that even this scenario would result in substantial excess of cases and deaths relative to a baseline scenario of not easing lockdown or elimination.
4. The excess cumulative cases are likely to be sensitive to the specified infection fatality ratio, although this is not expected to materially change the results and inferences. We have assumed a constant infection fatality rate across time, which would not account for changes in the age-composition of the infected cases over time.
5. The model inference in dependent on reliable reported statistics on incident deaths. Underestimation of recent registered deaths would lead to more conservative R inference, and underestimation of the impact of easing lockdown.

Introduction:

As countries around the world negotiated the first wave of the COVID-19 pandemic, governments had to make critical decisions about when and how they eased the lockdown measures instituted to control the pandemic. Given the significant risks of a resurgence of the pandemic and the consequent implications, these decisions have had important consequences on pandemic control following easing of lockdown restrictions globally.

Different countries eased lockdown in different ways, and at different points in their epidemic trajectory.[1] The UK imposed lockdown relatively late in its epidemic trajectory and began easing lockdown relatively early, when community transmission levels (incident cases) were still high.[2] By contrast, Germany, Denmark, Italy and Spain started easing lockdown when incident cases and deaths were at much lower levels. However despite mitigation strategies such as test, trace and isolation systems in place, countries like Germany saw increases in reproduction number (*R)* after easing lockdown, with increases to above 1 in June.[3] South Korea, and China too saw a resurgence in new cases after easing their lockdowns and went on to put in place localised restrictions to control the spread of infections.

Several experts, including SAGE, the scientific advisory body to the UK government, cautioned against easing lockdown in May 2020 [2], when community transmission was still high, warning that this could overwhelm the still nascent testing and contact tracing services that could mitigate the impact of easing lockdown, and greatly impact the health service. Nevertheless, the UK proceeded with easing lockdown with the stated aim of doing so while keeping $R \leq 1$. On the 13th May, people who could not work from home were asked to return to work. On the 1st June schools were re-opened, outdoor markets and showrooms opened and households were allowed to meet in socially distanced groups of six. On the 15th June non-essential businesses, including the retail sector, were opened. In the week of the 29th June, a surge in cases was reported in Leicester, England, leading to the re-imposition of restrictive measures, and concern that other regions in England may experience similar increases in case numbers.[4] Nevertheless, the government went ahead with the next planned easing of lockdown on the 4th of July, when pubs, cafes, and hotels opened.

For peer review only - http://bmjopen.bmj.com/site/about/guidelines.xhtml

As the country proceeded to rapidly ease lockdown, it was vital to understand and quantify the potential impact of this so as best inform public health strategy. In June 2020 we modeled these impacts across a range of plausible scenarios over the 90 day period from the 1st of June to the 29th of August. Using an epidemiological model of COVID-19 spread with Bayesian inference, we inferred parameters of the epidemic in England using daily death data from the Office of National Statistics (ONS). We estimated the time varying *R* and daily cases, and then used these to forecast cases and deaths in several plausible scenarios in which *R* increased with the easing of lockdown, particularly focusing on those in which *R* remained ≤1, and contrasted these with elimination strategies that aim to suppress *R* as much as possible.

During the manuscript review process, we were able to examine the observed data that accrued through the original forecasting period and compare it against the model predictions.

Methods

The original model inference and forecasting were carried out in June 2020 and the model development is described below. Following this, we describe the comparison of the model predictions from the original forecasts to the observed data from the forecasting period.

Data for model development:

In order to model the impact of easing lockdown, we needed to know the levels of transmission, and growth parameters of the regional epidemic. Given the limited community testing and case detection in the UK, incident case numbers at that point were likely to be substantially underestimated. We therefore based our model on the number of incident deaths by date of occurrence, which are likely to be more reliable.[5] Incident deaths are a function of incident cases in the previous weeks and the reproduction rate of the epidemic, and both these parameters can be inferred from the death data.[5] We included data till the 12th of June for England, as released by the ONS on the 30th of June 2020 (25th week of published data).[6] These data are based on deaths registered by the 27th of June. As reporting delays mean that more recent deaths are underestimated, we only considered deaths up to the 12th June.

Patient and public involvement

As only publicly available aggregate incident death statistics were utilised, there was no direct patient or public involvement.

Primary outcomes:

We assessed the excess cumulative predicted cases and deaths, over a 90-day period from the 1st June. We assumed different scenarios of changing *R* at the points of lockdown easing, in comparison with a baseline scenario in which *R* remained constant during this period.

Estimation of incident cases:

Incident cases, and time-varying *R* numbers were estimated using a Bayesian model, similar to that previously described by Flaxman et al,[5] accounting for the delay between onset of infection and death. The number of infected individuals is modelled using a discrete renewal process, as has been described before.[5] This is related to the commonly used Susceptible-Infected-Recovered (SIR) model, but is not expressed in differential form.

For peer review only - http://bmjopen.bmj.com/site/about/guidelines.xhtml

We modelled cases from 30 days prior to the first day that 10 cumulative deaths were observed in England, similar to previous methods.[5] The numbers of incident cases for the first 6 days of this period were set as parameters to be estimated by the model (**Table 1**).

Table 1: Parameters estimated by Bayesian model

Variable	Parameter	No.	Priors
c_t, where t=1…6	Number of initial cases on first six days	6	exponential(1.0/tau)
R_0	Baseline Reproduction Number	1	Normal(2.4,0.5)
R_t	Time varying effective reproduction number	9	Normal(0.8,0.25)
φ	variance parameter for negative binomial distribution of deaths	1	normal(0,5)
$\boldsymbol{\tau}$	parameter in prior of c_t	1	exponential(0.03)

Subsequent incident case numbers would then be a function of these initial cases, and estimated *R* values. We assumed a serial interval (SI) with a lognormal distribution with mean 4.7 and standard deviation (SD) of 2.9 days, as in Nishiura et al [7]. The SI was discretised as follows:

$$g_s = \int_{t=s-1}^{s} g(t)dt$$

For s=1,2…N, where N is the total number of intervals (each interval being 1 day) estimated. We estimated the distribution for 201 days, to align with the 111 days of data up to the 29th May, plus 90 days of forecasting. Given a SI distribution, the number of infections c_t on a given day *t*, is given by the following discrete convolution function:

$$c_t = R_t \Sigma_{j=0}^{t-1} c_j g_{t-j},$$

The incident cases on a given day *t,* are therefore a function of *R* at point *t* and incident cases up to time *t*−*1*, weighted by the distribution of the serial interval.

Estimation of time-varying reproduction number

The baseline reproduction number (R_0), and the subsequent time varying effective reproduction number (R_t) were estimated up to the 12th June. We allowed R_t to change on at least three points: (1) 16th March, when the UK first introduced social distancing measures; (2) 23rd March, when lockdown measures came into place with stay at home instructions and closures of schools and non-essential businesses; and (3) 13th May, the first easing of lockdown. We also considered models in which R_t was allowed to change on the 1st June. Given the limited death data i.e. only up to the 12th June, we were unlikely to be able to estimate changes in R_t after the 13th May with sufficient certainty. Observed deaths from the

For peer review only

For peer review only - http://bmjopen.bmj.com/site/about/guidelines.xhtml

1st June are likely to be a function of cases 2-3 weeks prior to this, and were unlikely to reflect changes in R_t from the 1st of June.

Model selection

We assessed and compared models that allowed R_t to change at the 4 points described above (Model 1), with more flexible models that allowed more frequent changes (Models 2 and 3), as follows:

1. Model 1: 16th March, 23rd March, 13th May and 1st June
2. Model 2: Every week from the beginning of the modelling period, including on the 16th March, 23rd March, 13th May and the 1st June
3. Model 3: 16th March, 23rd March, and 13th May, and every week between the 23rd March and 13th May i.e. during lockdown.

For each model, we used the R package *loo* to calculate expected log pointwise predictive density (ELPD) using Leave-one-out cross-validation (LOO) individually for each left out data point based on the model fit to the other data points. We then calculated between-model differences in ELPDs, to assess whether particular models predicted data better than others, as discussed previously.[8] As the assumptions in estimation of ELPD may be violated given these are time-series data, and therefore correlated, we also compared the root mean squared errors (RMSE) across models to assess fit. The final model used was arrived upon based on these comparisons, prioritising differences in ELPD, as this has been used in a similar context to assess change points, previously.[9] We assessed whether models were significantly different (ELPD difference/SE of difference >2). When models were not statistically significantly different in performance, for simplicity, we prioritised the model where the least number of parameters needed estimation.

In addition, we also compared Model 1 (four change points) with models where each of the change points were left out in turn, as done by Dehnig et al,[9] to assess if these dates do correspond to change points in R_t.

Estimation of deaths:

Incident deaths from COVID-19 are a function of the infection fatality rate (IFR), the proportion of infections that result in death, and incident cases that have occurred over the past 2-3 weeks. For observed daily deaths (D_t) for days $t \in 1, \ldots, n$, the expectation of observed daily deaths (d_t) is given by:

$$d_t = \mathrm{E}(D_t)$$

As described in Flaxman et al., we model the number of observed daily deaths D_t as following a negative binomial distribution with mean d_t and variance $d_t + \frac{d_t^2}{\psi}$, where ψ follows a half normal distribution:

$$D_t \sim Negative\ Binomial\left(d_t,\ d_t + \frac{d_t^2}{\psi}\right), \qquad \text{where } \psi \sim Normal^{+}(0,5).$$

For peer review only - http://bmjopen.bmj.com/site/about/guidelines.xhtml
For peer review only

Similar to estimation of incident cases, deaths at time point t (d_t) were modelled as a function of incident cases up to time $t-1$, weighted by the distribution of time of infection to time of death (π). The π distribution was modelled as the sum of the distribution of infection onset to symptom onset (the incubation period), and the distribution of symptom onset to death. As has been previously done,[5] both of these were modelled as gamma distributions with means of 5.1 days (coefficient of variation 0.86) and 18.8 days (coefficient of variation 0.45), respectively as follows:

$$\pi \sim IFR * (Gamma(5.1, 0.86) + Gamma(18.8, 0.45))$$

IFR was assumed to be 1.1%, based on the most recent estimates from the University of Cambridge MRC Nowcasting and Forecasting model.[10] This estimate is in line with estimates from Flaxman et al. (Imperial), of 1% that have been widely used in modelling of COVID-19 deaths across the UK.[11] These estimates are based on the those reported by Verity et al.,[12] during early epidemiological inference from the outbreak in Wuhan, and are corrected for age structure, and contact patterns for the UK, as previously outlined.[11] Misspecification of the IFR estimate would lead to biased inference of case numbers, but not deaths, as this can be considered as a scaling factor, that is used first to estimate the cases, which are then used to accurately predict observed deaths, and future deaths based on different scenarios. Therefore, the predicted death numbers can be thought of as independent of these estimates. For simplicity, we consider a fixed IFR over time.

To discretise the time to death distribution, we estimated the probability of death within each discrete time interval (1 day), conditional on surviving previous intervals. First, we calculate the hazard (h_t) the instantaneous probability of failure (i.e. dying) within a time interval, as follows:

$$h_t = \frac{\int_{t = s - 0.5}^{s + 0.5} \pi(t)dt}{1 - \pi_{s - 0.5}}$$

As the denominator excludes individuals who have died, this ensures that h_t is calculated only among those surviving. The probability of survival within each interval is:

$$s_t = 1 - h_t$$

The cumulative survival probability of surviving up to the interval $t-1$ is therefore:

$$S_{T > t - 1} = \prod_{j = 1}^{t - 1} s_j$$

, where T is the time of death of an individual. In other words the cumulative probability of survival up to interval t is simply the product of survival within each interval up to t-1, where the probability of survival within each interval (s_t) is $1-h_t$, where h_t is the probability of dying within that interval.

Given this, we now estimate the probability of death within interval t, conditional on surviving up to $t-1$ as:

For peer review only

$$\omega_t = P(T = t|\, T > t-1) = \, S_{T\,>\,t\,-\,1} * h_t$$

Here ω represents the discretised distribution of infection onset to death, with the probability of death within interval *t* conditional on surviving previous intervals. Deaths can therefore be calculated as a function of incident cases of infection within previous intervals, as follows:

$$d_t = \sum_{j=0}^{t-1} c_j \omega_{t-j}$$

Here, the number of deaths within interval *t* (on a given day) is a sum of the number of daily cases up to the previous day, with previous cases weighted by the discretised probability distribution of time from onset of infection to death.

Estimated parameters and model priors:

We estimated the set of model parameters θ={$c_{1\text{-}6}$, R_0, R_t, φ, $\boldsymbol{\tau}$} using Bayesian inference with Markov-chain Monte-Carlo (MCMC) (**Table 1**). We estimated the number of cases in the first six days of the modelled period, as subsequent cases are simply a function of cases on these days, the SI, and R_t . As described above, R_0 was constrained up to the 16th March and then again after the 13th of May. For the period prior to 16th March, we assigned a normal prior for R_0 with mean 2.5 and SD 0.5. For the period that R_t was allowed to vary i.e. every week from the 16th of March till the 13th of May, we assigned a normal prior with a mean 0.8 and SD 0.25. These priors are based on estimates of time changing R_t from the University of Cambridge MRC biostatistics nowcasting and forecasting models[10] and SAGE estimates of *R*,[13] and consistent with Flaxman et al.[5] For the number of cases on day 1, we assigned a prior exponential distribution:

$$y \sim exponential\left(\frac{1}{\tau}\right)$$

where $$\tau \sim exponential(0.03)$$

Model estimation:

Parameters were estimated using the Stan package in R with Markov chain Monte Carlo (MCMC) algorithms used to approximate a posterior distribution of parameters by randomly sampling the parameter space. We used 4 chains with 1000 warm up samples (which were discarded), and 3000 subsequent samples in each chain (12,000 samples in total) to approximate a posterior distribution using the Gibbs Sampling algorithm. From these we obtained the best-fit values and the 95% credible intervals for all parameters. We used these parameters to estimate the number of incident cases and deaths in England. We examined the fit of the model predicted deaths to the observed daily deaths from the ONS, and also the consistency of the model parameters with known values in the literature, estimated from global data. We assessed the distribution of *R-hat* values for all parameters, to assess convergence between chains.

Sensitivity analyses:

We carried out sensitivity analyses using uninformative priors for R_0 and R_t, to examine the sensitivity of R_t estimates to prior specification. We also examined the impact of the SI by comparing the baseline model (SI of mean 4.7 and SD 2.9 days), with a longer SI modelled as

For peer review only - http://bmjopen.bmj.com/site/about/guidelines.xhtml

a gamma distribution with mean 6.5 and coefficient of variation of 0.72, as estimated by Chan et al.[14]

Forecasting cases and deaths:
All forecasts were carried out up to 90 days (29th August 2020) after the 1st of June. We considered a set of scenarios in which R_t increased from baseline on the 1st of June and then remained constant, as well as those in which further increases in R_t occur on the 15th June and the 4th July. We considered an increase in R_t of up to 0.25 in increments of 0.05, this being a plausible degree of change in response to easing lockdown, based on the empirical data from other countries,[3,15] as well as the modelling by UK SAGE.[16] Finally, for comparison with a strategy of elimination, namely suppressing R_t to the lowest level possible before easing lockdown measures, as has been done South Korea, New Zealand and Australia, we also modelled scenarios with R_t values of 0.6 and 0.7.

For each of these scenarios, we predicted the number of incident cases, and incident deaths, using the functions from the inference model above. Briefly cases are a function of R_t, incident cases on previous days and the SI discretised distribution:

$$c_t = R_t \sum_{j=0}^{t-1} c_j g_{t-j},$$

Deaths are a function of incident cases over previous weeks, and the distribution of onset of infection to death times:

$$d_t = \sum_{j=0}^{t-1} c_j \omega_{t-j}$$

All scenarios were compared to a baseline scenario of no change in R_t from the 13th of May onwards.

Comparison of model predictions to observed data:
The observed death data for daily deaths in England up to the 28th of August as obtained from the ONS (from data up to the 11th September) were plotted against the original model predictions from June, and the root mean square error was calculated between the observed data and the predicted deaths in the different modelled scenarios. The model was rerun with these data, to infer values of R_t till the 28th of August. As the purpose of this exploratory model was inference of parameters, R_t was allowed to change weekly from the 16th March, as well as at time points of easing lockdown: 13th of May, 1st June, 15th June and 4th July as in the original forecasting and the 25th of July (gyms and pools reopened), and the 15th of August (casinos, bowling alleys and soft play areas reopened). Where these dates fell on the weekly change point, they were not included separately.

Results

Model selection and model inferences
Model 3, which allowed weekly changes in R_t during lockdown, produced the best fit to the data (**Supplementary Table 1**), with estimation of fewer parameters compared with Model 2.

For peer review only - http://bmjopen.bmj.com/site/about/guidelines.xhtml

This was therefore used as the primary model and unless otherwise stated, all inferences described subsequently are from this model.

We inferred R_0 of 3.65 (95% credible intervals (CI) 3.36-3.96), consistent with previous estimates within the UK.[5] The R_t is estimated to have declined substantially following initiation of social distancing, and lockdown measures, reaching a low of 0.66 (95% CI 0.34-1.04) during the week 30th March-5th April 2020. The most recent R_t from the 13th of May is estimated as 0.752 (95% CI 0.50-1.00) (**Figure 1**). The alternative models allowing change of R_t on the 1st of June inferred a very similar R_t for the 1st -12th June suggesting that there was insufficient data to accurately infer any changes to R_t following the easing of lockdown on 1st June. On examining the impact of constraining R_t on model fit at any of the 4 change points, this appears greatest for the 16th March (when social distancing measures were put into place) (**Supplementary Table 2**) with only modest impacts on model fit of constraining R_t on 23rd March and 13th May, and no impact on constraining R_t on the 1st June.

The model showed a good fit to the observed distribution of deaths up to the 12th June (**Figure 2**). *Rhat* estimates were < 1.05 for all estimated parameters (**Supplementary Figure 1**). Leave one out cross-validation also supported a good model fit, with the shape parameter k<0.5 for all values (**Supplementary Figure 2**). The median number of incident cases inferred on the 1st June was 4,317/day (95% CI 2,062-8,155), which was broadly consistent with the estimates from the ONS survey for England based on a random sample of the population within the same time period.

Forecasts of lockdown easing scenarios

In the baseline forecasting scenario where R_t remained constant (R_{test}=0.75) through the 90-day forecasting period (1st June to 29th August 2020), the model predicted 48,501 (46,170-50,989) cumulative deaths in England (**Supplementary Table 3**). By comparison, the ONS reported 46,539 cumulative deaths up to 12th June in England (registered up to 27th June).

In the scenarios where R_t increased on the 1st of June and then remained constant, for increases from the median 0.75 to 0.80, 0.85, 0.90, 0.95 and 1, the model predicted median excess deaths of 257 (95% CI 108-492), 632 (95% CI 265-1,208), 1,173 (95% 493-2,240), 1,971(95% 828-3,764) and 3,174 (95% CI 1,334-6,060) respectively. Increases of R_t to 1.05 and 1.1, with resultant exponential growth, led to excess median deaths of 5017 (95% CI 2,109-9,578), and 7,878 (3,313-15,037) respectively (**Figure 3** and **Supplementary Table 3**).

In scenarios where R_t increased on the 1st June, 15th June and 4th July, we found that compared to the baseline scenario, modest increases of R_t to 0.80, 0.85, 0.90, on these dates respectively would lead to 508 (95% CI 213-972) excess deaths. If R_t increased to 0.90, 0.95 and 1 at these time points, then excess estimated deaths increase to 1,848 (95% CI 776-3,534). In these scenarios R_t remains ≤1 (**Figures 3-5** and **Supplementary Table 3**). Increases of R_t above 1 at any point resulted in rapid increases in cases, and deaths, with between 3,600-13,000 excess deaths in different scenarios for R_t rising up to between 1 and 1.2, predicting a second wave of the epidemic within England (**Figure 4-5** and **Supplementary Table 3**).

Even in the conservative scenario where R_t increased from 0.75 to 0.80 on the 1st of June and then remained constant thereafter, the model predicted an excess of 26,447 (95% CI 11,105-

For peer review only - http://bmjopen.bmj.com/site/about/guidelines.xhtml

50,549) cumulative cases over 90 days. On the other hand, the scenario with the largest changes in R_t, but still remaining ≤1, predicted an excess of up to 421,310 (95% CI 177,012-804,811) (**Figures 6-8** and **Supplementary Table 3**). For scenarios where R_t rose beyond 1 (up to 1.2), we would expect between 540,000 to 2.8 million excess cases, in line with a second wave (**Supplementary Table 3**).

Forecasts from an elimination scenario

Compared to the baseline scenario of R_t staying at 0.75, we found that maintaining R_t at 0.60 and 0.70 would result in 44,302(95% CI 84684-18600) and 19,968 (95% CI 38168-8384) fewer cumulative cases, and 462 (95% CI 194-884) and 204 (95% CI 389-86) fewer deaths over the modelled 90-day period, respectively **(Figure 3, Figure 6, Supplementary Table 3**).

Robustness of model in sensitivity analyses

Using uninformative (no prior specified) priors for R_t did not materially alter the median estimates of R_t, although uncertainty around estimates was predictably increased (**Supplementary Figure 3**). This suggests our estimates are robust to the priors specified.

Using a longer SI leads to an increase in the estimated R_0, although subsequent estimates following easing of lockdown remain broadly comparable (**Supplementary Figure 4**). This model is comparable to the primary model with regard to fit to observed deaths (**Supplementary Figure 5**) although we note that predicted excess deaths and cases in all scenarios where R_t<1.1, are higher than in the primary model with shorter serial interval (**Supplementary Table 4**), suggesting the primary model is likely to be conservative.

Comparison of model predictions to observed data:

The observed cases and deaths are plotted against the modelled scenarios in **Figures 9**. Among the scenarios studied, the observed daily deaths seems to align most closely with the scenario in which R values are 0.85, 0.85 and 0.9 at the 3 change points. The RMSE between the observed and predicted deaths is lowest for this scenario (**Supplementary Table 3**). The inferred R_t values concur with this (although uncertainty estimates are wide), and also suggest that it is in late July that R_t started to creep above 1 (**Figure 10**). We also note that the observed cumulative deaths by the 28th August represent an excess of 1,291 deaths over our baseline scenario.

Discussion

In this paper we describe a Bayesian model for inferring incident cases and reproduction numbers from daily death data, and for forecasting the impact of future changes in *R*. Our findings provide important quantification of the likely impact of relaxing lockdown measures in England, and to our knowledge, this is the first study to have comprehensively assessed this through several plausible scenarios. We show that even in scenarios in which *R* remains ≤1 (in line with the UK government's stated aim), small increases in R_t from lifting lockdown measures, can lead to a substantial excess of deaths with 3,174 (95% CI 1,334-6,060) in the most severe scenario modelled.

For peer review only - http://bmjopen.bmj.com/site/about/guidelines.xhtml

Our model inferences are robust to modelling assumptions of specified priors for R_t. We note, however, that using a longer serial interval would results in higher numbers of excess deaths for each scenario, suggesting that our primary scenario is conservative (**Supplementary Tables 3** and **4**). Our estimated R_t of 0.75 following 13th May is consistent with estimates from the SAGE group advising government at the time.[13] We assessed increases in R_t that were entirely plausible, given the data from other European countries that have started easing lockdown.[3] Our model predicted a substantial excess of cases and deaths in several scenarios where R remained ≤1, as well as scenarios where R increased up to 1.2. When we compared our predictions to the observed data from the original forecasting period we found that these aligned most closely to the scenario in which R increased to 0.85 on the 1st June, and then to 0.9 on the 4th July. In contrast, our model showed that had an elimination strategy been pursued and R_t suppressed to 0.6 or 0.7, this could have prevented a median estimated 462 and 204 deaths, and 44,302, and 19,968 cases, respectively from the baseline scenario.

Countries like Denmark and Germany started easing lockdown when community transmission was low and this likely mitigated increases in R with the lifting of lockdowns, alongside the use of aggressive case detection and contact tracing approaches. The UK began to ease lockdown when community transmission was still high (with daily estimated >8000 cases and >300 deaths) and still does not have a fully operational test, trace and isolate system at the time of writing, with the existing system overwhelmed by incident cases. The UK's current estimates of R_t still rely on incident deaths (as used by the MRC Nowcasting and Forecasting model, and SAGE)[10] , and therefore reflect community transmission from a median of 2-3 weeks ago.[13] Easing lockdown in 2-weekly steps, meant that by the time we detected the impact of one step, the next one had already been instituted and not unexpectedly, mitigating these impacts was challenging. At the time lockdown was being rapidly eased UK SAGE expressed concerns that increases in R up to 1.2 could continue undetected for longer periods of time.[16]

In September 2020, the UK is at point where community transmission is once again high and it is clear that we have entered the second wave of the pandemic. Schools reopened in the second week of September, a move that is vitally important to children's health and development, but one that can potentially increase community transmission. Cases and hospitalisations have been increasing exponentially, which has recently translated into an increase in weekly deaths. Using the best available confirmed COVID-19 case data in England published by the UK government on the 21st September (which is likely an underestimate), we modelled the potential impact of increases in transmission on daily cases and deaths over the next two months, assessing different scenarios of increase in R_t. As R_t reaches 1.5, the daily deaths approach 1,000 by late November (**Figure 11**). We note that the number of deaths forecast during this period could be overestimated if transmission is disproportionately higher among younger age groups, as overall IFR would be lower than the assumed 1%. However, as current data suggests, transmission is likely to spill over into more vulnerable, and older age groups over time. This has profound implications for the health service and the limited ICU capacity available in the NHS, which is at great risk of being overwhelmed. Our modelling suggests that small changes in R_t moving forward could have substantially large effects on case numbers, and deaths, suggesting that mitigatory strategies implemented in a timely manner could have a large impact.

For peer review only - http://bmjopen.bmj.com/site/about/guidelines.xhtml

We acknowledge some important limitations of our model. The first is that it is based on a back calculation of cases based on incident deaths, which are likely to underestimated due to reporting delays and underreporting. Second, our model is reliant on inferring cases, and reproduction numbers, which depend on the assumed distributions of the serial interval, and the time of onset to death distributions. Though we based our assumptions on the literature, misspecification of these would influence our estimates. While we have evaluated this, greater deviations from true estimates would make our forecasting less reliable. Third, similar to Flaxman et al,[11] our model uses the IFR as a multiplier for the distribution of time from infection to death, in the absence of reliable population level case fatality rates (CFR). While this would not affect the estimation of deaths, if the CFR were higher (due to large proportions of cases being asymptomatic), then the predicted case numbers would be overestimated by our model. We note, however that the estimate of IFR we used (1.1%) is consistent with the CFR estimated previously from Beijing[17] and Flaxman et al.[11] We have also, for simplicity, assumed that IFR remains constant throughout the pandemic and the forecasting period. Given that age is an important determinant of mortality, our model may not reflect the changes in the age-composition of infected individuals, and changes in healthcare, and treatments over time, influencing the accuracy of inference, and forecasting. Unfortunately, the ONS does not provide age-stratified daily death data for England to allow us to model differences in age-structure. We have therefore, not considered these in our inference or forecasting. We note that if cases occur disproportionately in younger populations following easing of lockdown, excess deaths may be overestimated during our forecasting period. Fourth, we did not consider the impact of mitigatory measures in our current modelling. However, as we have seen, mitigatory measures were implemented with significant delays from when community transmission increased, as many experts had expected. Nevertheless if implemented with sufficient rigour and coverage, mitigatory measures would reduce the impact of the modelled scenarios. We note that our inferred R_t based on recent death data should reflect the impact of mitigatory measures, such as testing, contract tracing and isolating, as well as mask use, as inferred R_t values were allowed to change every week. Finally, we only modelled a limited set of scenarios, mainly restricted to those in which R_t remained ≤ 1.2 but there are multiple possible scenarios that could be modelled. We note that the scenarios modelled are in line with R_t ranges that were subsequently inferred from current death data.

In summary, we show that increases in R_t as a result of easing lockdown would have a substantial impact on incident transmission and deaths for even modest increases that still maintain $R_t \leq 1$, and an even greater impact should R_t rise above 1. This has subsequently been borne out by the observed data. Our findings and the observed data thus far argue strongly for a much more cautious approach in public health management, an urgent need for a properly functioning test, trace and isolate system, with adequate support for isolation,[18] robust mitigatory measures in schools[19] and serious consideration of elimination strategy alongside vaccine-roll out to control the pandemic. Such a multi-pronged approach aimed at elimination is necessary and its value has been clearly demonstrated in terms of lower case numbers, fewer deaths and lower economic impacts in countries that have followed such strategies.[20] This is all the more important given that continuing transmission has favoured adaptation of SARS-CoV-2, with emergence of several variants of concern some of which are more transmissible, more able to escape immunity from vaccines, or both.

For peer review only - http://bmjopen.bmj.com/site/about/guidelines.xhtml

Elimination allows us to reduce uncertainty associated with new variants, and conserves vaccine effectiveness by preventing emergence of new variants that may threaten this.

Authors' contributions:
DG conceived the study and designed the model with NS. DG programmed the model and made the figures. HZ and NS consulted on the model design. All authors interpreted the results, contributed to writing the Article, and approved the final version for submission.

Declaration of competing interests:
None.

Funding:
HZ is partly funded by the Bernard Wolfe Health Neuroscience Fund at the University of Cambridge. NS is funded by a Strategic Award from the Wellcome Trust (208363/Z/17/Z). DG is funded by the UKRI/Rutherford HDR-UK fellowship programme (reference MR/S003711/2), and the NIHR AIM Development award (reference NIHR202646).

Data sharing:
All data on daily deaths used in this study were taken from the Office of National Statistics website (https://www.ons.gov.uk/peoplepopulationandcommunity/birthsdeathsandmarriages/deaths/datasets/weeklyprovisionalfiguresondeathsregisteredinenglandandwales).

The code for the model, and dataset analysed is available at: https://github.com/dgurdasani1/lockdownsim

Ethics
No ethical approval was obtained for this study, as only publicly available aggregate data on incident deaths was analysed.

Acknowledgements:
We would like to acknowledge and thank Flaxman et al and the team at Imperial College London for making their code available for us to use.

References:

1. Coronavirus: How lockdown is being lifted across Europe. BBC. 2020.
2. Gallagher J. Coronavirus: Risk in UK lockdown easing too soon, warn scientists. BBC. 2020.
3. Robert Koch Institute G. Coronavirus Disease 2019 (COVID-19), Daily Situation Report of the Robert Koch Institute 2020.
4. Leicester lockdown tightened as coronavirus cases rise. BBC. 2020.
5. Flaxman S MS, Gandy, A, Unwin HJT, Coupland H, Mellan, TA, Zhu H, Berah T, Eaton JW Report 13: Estimating the number of infections and the impact of non-pharmaceutical interventions on COVID-19 in 11 European countries: Imperial College, London, 2020.

For peer review only - http://bmjopen.bmj.com/site/about/guidelines.xhtml

6. Deaths registered weekly in England and Wales, provisional. In: Statistics OoN, editor.; 2020.
7. Nishiura H, Linton NM, Akhmetzhanov AR. Serial interval of novel coronavirus (COVID-19) infections. *Int J Infect Dis* 2020; **93**: 284-6.
8. Vehtari A, Gelman, A., and Gabry, J. Practical Bayesian model evaluation using leave-one-out cross-validation and WAIC. Statistics and Computing. *Statistics and Computing* 2017; **27**(5): 1413-32.
9. Dehning J, Zierenberg J, Spitzner FP, et al. Inferring change points in the spread of COVID-19 reveals the effectiveness of interventions. *Science* 2020.
10. Joint PHE Modelling Cell MBUC-WG. COVID-19: nowcast and forecast, 2020.
11. Flaxman S, Mishra S, Gandy A, et al. Estimating the effects of non-pharmaceutical interventions on COVID-19 in Europe. *Nature* 2020.
12. Verity R, Okell LC, Dorigatti I, et al. Estimates of the severity of coronavirus disease 2019: a model-based analysis. *Lancet Infect Dis* 2020; **20**(6): 669-77.
13. Gov.uk. Government publishes coronavirus (COVID-19) R value and growth rates. 2020.
14. Chan Y-W D FS, Lam T-L T, Leung M-H J, Wong M-L, Lam, H-Y, Chuang S-K. Transmission dynamics, serial interval and epidemiology of COVID-19 diseases in Hong Kong under different control measures. Wellcome Open Research; 2020.
15. STATUS FOR SMITTETRYKKET I DANMARK, 29/4 2020: Statens Serum Institut.
16. SAGE. Thirty-eighth SAGE meeting on COVID-19, 21st May 2020, 2020.
17. Leung K, Wu JT, Liu D, Leung GM. First-wave COVID-19 transmissibility and severity in China outside Hubei after control measures, and second-wave scenario planning: a modelling impact assessment. *Lancet* 2020; **395**(10233): 1382-93.
18. Gurdasani D, Bear L, Bogaert D, et al. The UK needs a sustainable strategy for COVID-19. *Lancet* 2020; **396**(10265): 1800-1.
19. Gurdasani D, Alwan NA, Greenhalgh T, et al. School reopening without robust COVID-19 mitigation risks accelerating the pandemic. *Lancet* 2021; **397**(10280): 1177-8.
20. Oliu-Barton M, Pradelski BSR, Aghion P, Artus P, Kickbusch I, Lazarus JV. SARS-CoV-2 elimination, not mitigation, creates best outcomes for health, the economy, and civil liberties. *The Lancet* 2021.

For peer review only

Figure Legends

Figure 1: Estimated time-varying reproduction number (R_t) for England

The figure shows the R_t estimated by Model 3 (blue) with 95% credible intervals (grey) with a serial interval of mean 4.7 and SD 2.9 days. From 3.65 (CI 3.36-3.96), R_t drops on the 16th March and 23rd March (indicated by vertical dashed lines) when social distancing and lockdown were instituted, reaching a low of 0.66 (95% CI 0.34-1.04) in the week of the 30th March. The last estimated R_t is 0.75 (95% CI 0.50-1.00) following the 13th May.

Figure 2: Model fit to observed death data

Daily deaths predicted by Model 3 (blue) with 95% credible intervals (grey) show a good fit to the observed deaths from the ONS (red)

Figure 3. Predicted deaths with R_t increasing on 1st June

For peer review only - http://bmjopen.bmj.com/site/about/guidelines.xhtml

(A) The model compared scenarios in which R_t increases to 0.80 (light green), 0.85 (green), 0.90 (dark blue), 0.95 (red), 1 (purple) and 1.05 (brown) and then remains constant for the 90-day forecasting period. The comparator baseline scenario is of R_t remaining at 0.75 (black) and two elimination strategies of R_t reducing to 0.7 (yellow) and 0.6 (light blue) were also considered. Vertical dashed lines represent time-points of easing lockdown. (B), (C) the incident and cumulative deaths increase in all scenarios in which R_t increases and reduces in the two elimination scenarios.

Figure 4. Predicted deaths in scenarios of R_t increase on 1st and 15th June compared with baseline scenario

(A) The model compared scenarios in which R_t increases to 0.80 (light green), 0.85 (green), 0.90 (blue), 0.95 (red), 1 (purple) and 1.05(brown) and then further by 0.05 on the 15th June and then remaining constant for the 90-day forecasting period. The comparator baseline scenario is of R_t remaining at 0.75 (black). Vertical dashed lines represent time-points of easing lockdown. (B), (C) The incident and cumulative deaths increase in all scenarios in which R_t increases.

Figure 5. Predicted deaths in scenarios of R_t increase on 1st June, 15th June and 4th July compared with baseline scenario

(A) The model compared scenarios in which R_t increases to 0.80 (light green), 0.85 (green), 0.90 (blue), 0.95 (red), 1 (purple) and 1.05(brown) and then further by 0.05 on the 15th June and then again by 0.05 on the 3rd July before remaining constant for the 90-day forecasting period. The comparator baseline scenario is of R_t remaining at 0.752 (black). Vertical dashed lines represent time-points of easing lockdown. (B), (C) The incident and cumulative deaths increase in all scenarios in which R_t increases.

Figure 6. Predicted cases in scenarios of R_t increase on 1st June compared with baseline and elimination scenarios

(A) The model compared scenarios in which R_t increases to 0.80 (light green), 0.85 (green), 0.90 (dark blue), 0.95 (red), 1 (purple) and 1.05(brown) and then remains constant for the 90-day forecasting period. The comparator baseline scenario is of R_t remaining at 0.752 (black) and two elimination strategies of R_t reducing to 0.7 (yellow) and 0.6(light blue) were also considered. Vertical dashed lines represent time-points of easing lockdown. (B), (C) the incident and cumulative cases increase in all scenarios in which R_t increases and reduces in the two elimination scenarios.

Figure 7. Predicted cases in scenarios of R_t increase on 1st June and 15th June compared with the baseline scenario

(A) The model compared scenarios in which R_t increases to 0.80 (light green), 0.85 (green), 0.90 (blue), 0.95 (red), 1 (purple) and 1.05(brown) and then further by 0.05 on the 15th June and then remaining constant for the 90-day forecasting period. The comparator baseline scenario is of R_t remaining at 0.752 (black). Vertical dashed lines represent time-points of easing lockdown. (B), (C) The incident and cases increase in all scenarios in which R_t increases.

Figure 8. Predicted cases in scenarios of R_t increase on 1st June and 15th June and 4th July compared with the baseline scenario

For peer review only

(A) The model compared scenarios in which R_t increases to 0.80 (light green), 0.85 (green), 0.90 (blue), 0.95 (red), 1 (purple) and 1.05(brown) and then further by 0.05 on the 15th June and then again by 0.05 on the 3rd July before remaining constant for the 90-day forecasting period. The comparator baseline scenario is of R_t remaining at 0.752 (black). Vertical dashed lines represent time-points of easing lockdown. (B), (C) The incident and cumulative cases increase in all scenarios in which R_t increases.

Figure 9. Predicted deaths in different scenarios of R_t increase on 1st June, 15th June and 4th July compared with the baseline scenario, and real observed death data from the ONS (light green).

The model compared scenarios in which R_t increases to different values on the 1st, 15th and 4th of July with real observed deaths (light green). The comparator baseline scenario is of R_t remaining at 0.752 (black). Vertical dashed lines represent time-points of easing lockdown. (B), (C) The incident and cumulative deaths increase in all scenarios in which R_t increases. The daily deaths appear to fit best with the scenarios where R_ts are between 0.85 and 0.95 (dark blue, light blue, and purple) during this period.

Figure 10: Estimated time-varying reproduction number (R_t) for England

The figure shows the R_t estimated from the recent ONS death data (up to September 11, 2020) with 95% credible intervals (grey) with a serial interval of mean 4.7 and SD 2.9 days. We see a gradual upward trend in inferred R_t, with median R_t rising above 1 toward the end of July.

Figure 11: Predicted cases and deaths at different R_t values from current case numbers in England as of 21st September 2020

Figure 11 represents the predicted rise in cases based on different R_t values, and a serial interval of mean 4.7 and SD 2.9 days. The case numbers were calculated as a moving 7 day average from the Public Health England data of confirmed cases within England up to the 21st September. We project case, and death numbers (assuming an IFR of 1%) from these incident case numbers, using different scenarios of R_t. We note that case numbers are likely underestimates, as the testing system within England is currently running at capacity, and not everyone with symptoms is able to access tests.

For peer review only

For peer review only - http://bmjopen.bmj.com/site/about/guidelines.xhtml

Figure 1: Estimated time-varying reproduction number (R_t) for England

The figure shows the R_t estimated by Model 3 (blue) with 95% credible intervals (grey) with a serial interval of mean 4.7 and SD 2.9 days. From 3.65 (CI 3.36-3.96), R_t drops on the 16th March and 23rd March (indicated by vertical dashed lines) when social distancing and lockdown were instituted, reaching a low of 0.66 (95% CI 0.34-1.04) in the week of the 30th March. The last estimated R_t is 0.75 (95% CI 0.50-1.00) following the 13th May.

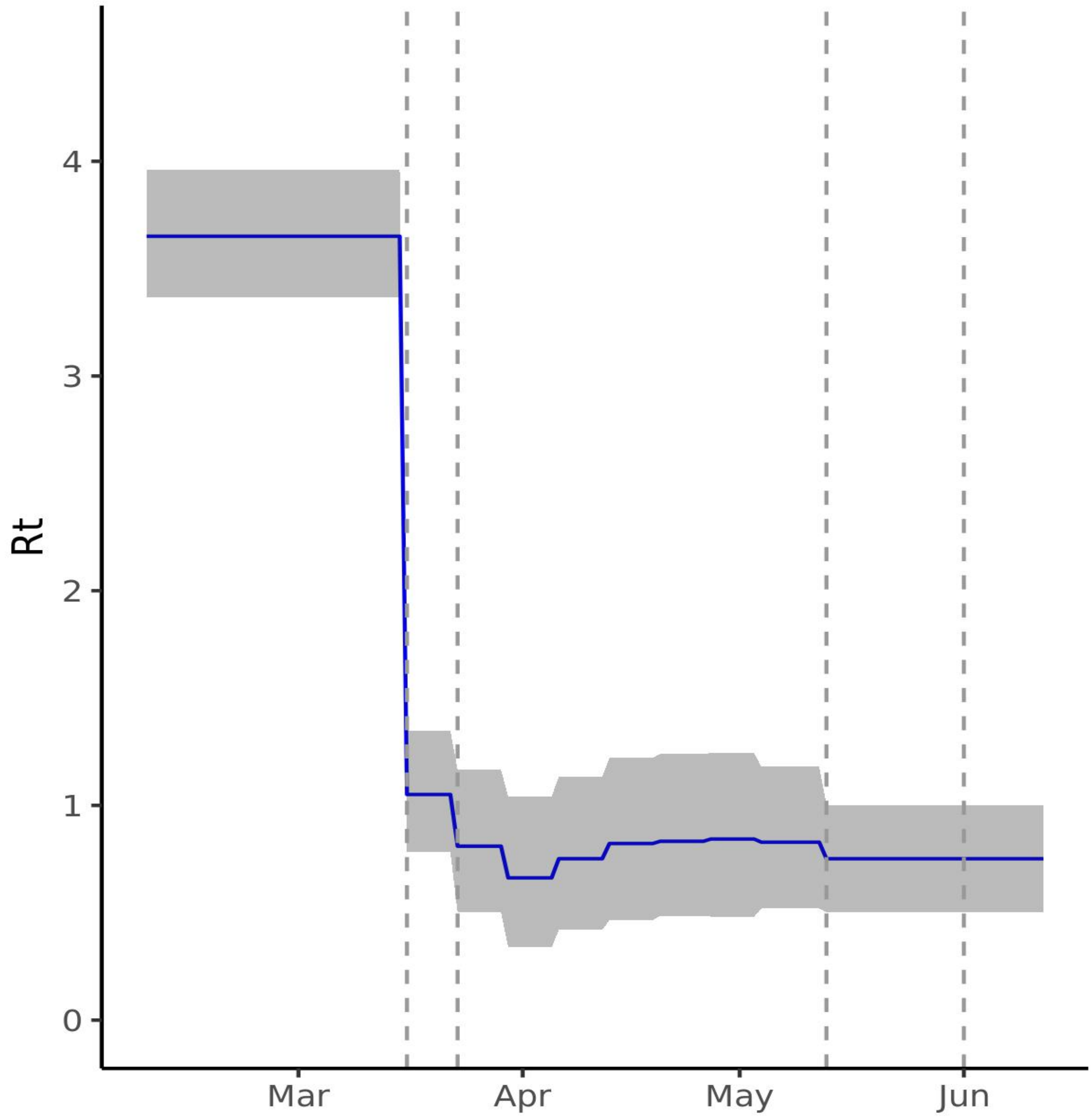

Figure 2: Model fit to observed death data
Daily deaths predicted by Model 3 (blue) with 95% credible intervals (grey) show a good fit to the observed deaths from the ONS (red)

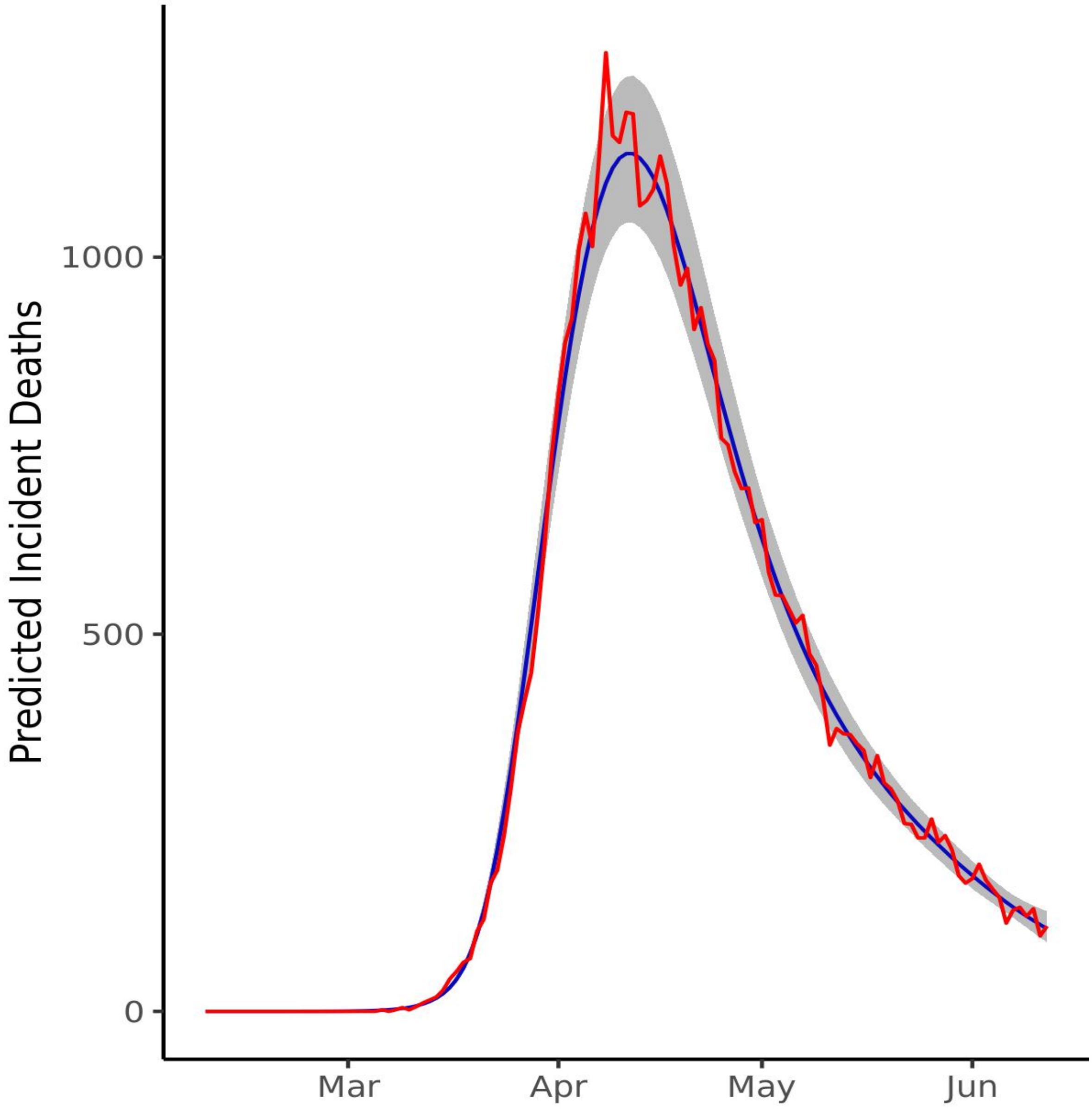

Figure 3. Predicted deaths with R_t increasing on 1st June

(A) The model compared scenarios in which R_t increases to 0.80 (light green), 0.85 (green), 0.90 (dark blue), 0.95 (red), 1 (purple) and 1.05 (brown) and then remains constant for the 90-day forecasting period. The comparator baseline scenario is of R_t remaining at 0.75 (black) and two elimination strategies of R_t reducing to 0.7 (yellow) and 0.6 (light blue) were also considered. Vertical dashed lines represent time-points of easing lockdown. (B), (C) the incident and cumulative deaths increase in all scenarios in which R_t increases and reduces in the two elimination scenarios.

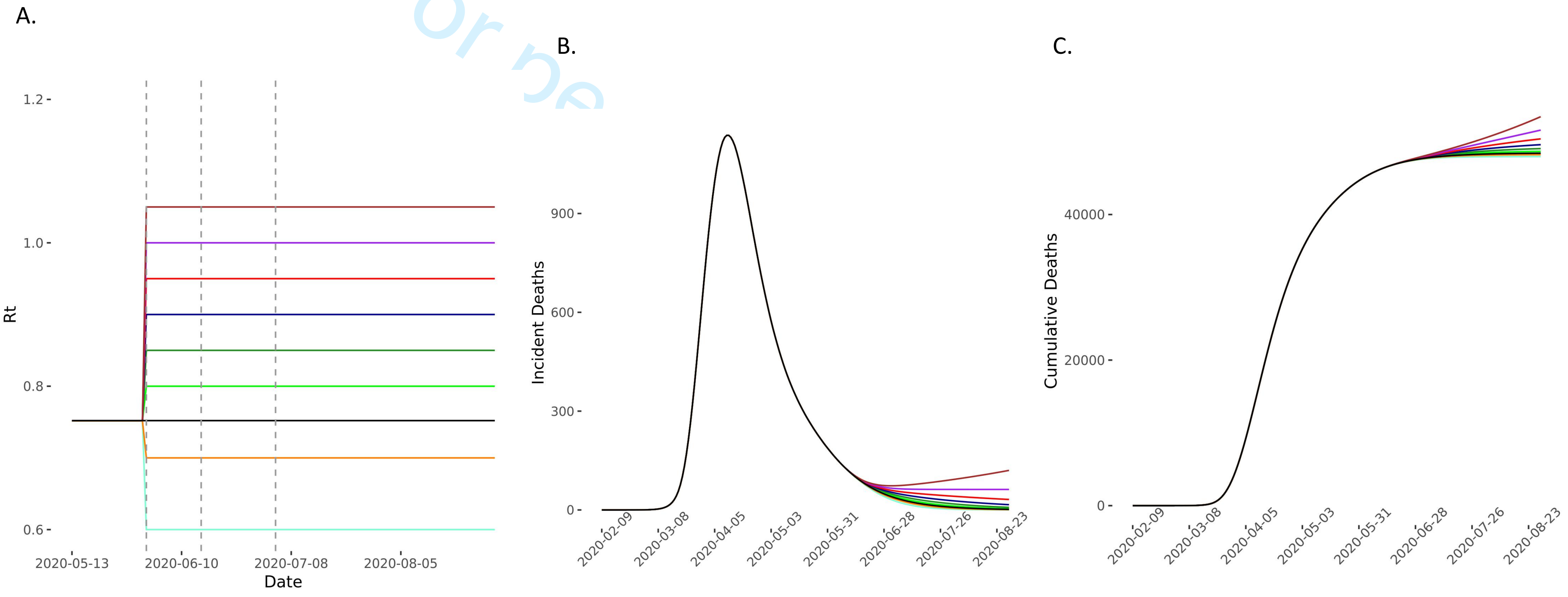

Figure 4. Predicted deaths in scenarios of R_t increase on 1st and 15th June compared with baseline scenario

(A) The model compared scenarios in which R_t increases to 0.80 (light green), 0.85 (green), 0.90 (blue), 0.95 (red), 1 (purple) and 1.05(brown) and then further by 0.05 on the 15th June and then remaining constant for the 90-day forecasting period. The comparator baseline scenario is of R_t remaining at 0.75 (black). Vertical dashed lines represent time-points of easing lockdown. (B), (C) The incident and cumulative deaths increase in all scenarios in which R_t increases.

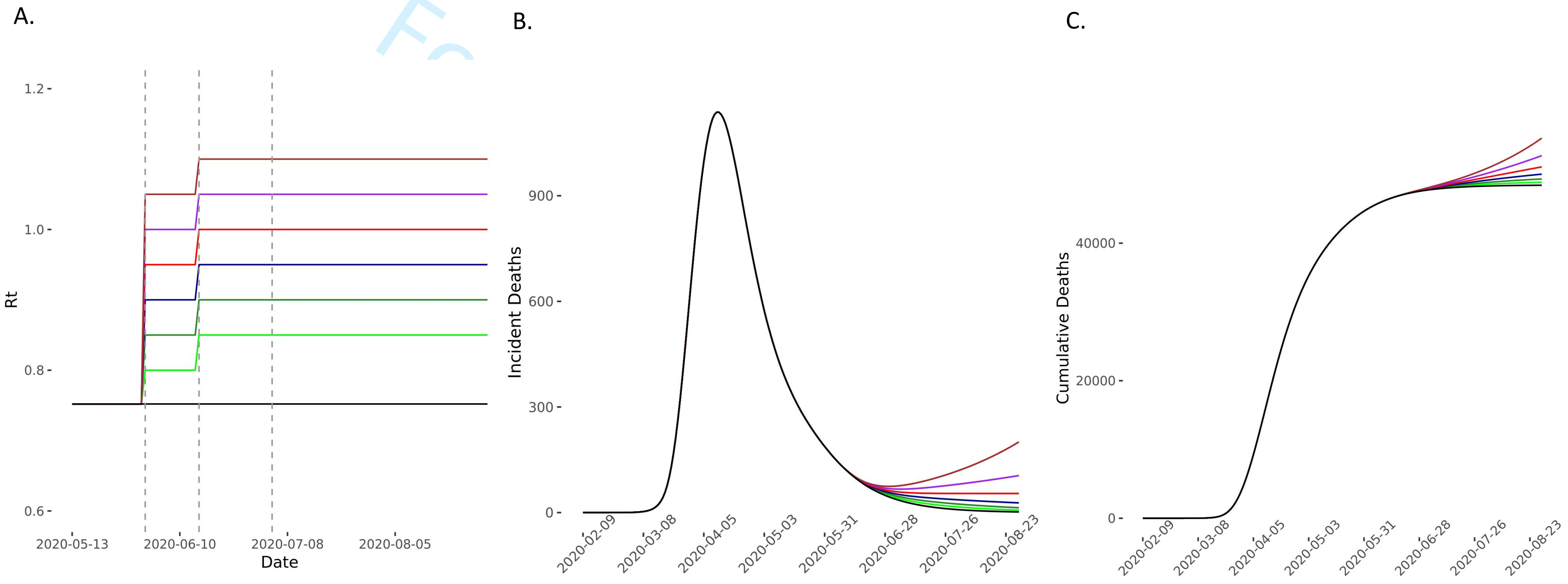

Figure 5. Predicted deaths in scenarios of R_t increase on 1st June, 15th June and 4th July compared with baseline scenario

(A) The model compared scenarios in which R_t increases to 0.80 (light green), 0.85 (green), 0.90 (blue), 0.95 (red), 1 (purple) and 1.05(brown) and then further by 0.05 on the 15th June and then again by 0.05 on the 3rd July before remaining constant for the 90-day forecasting period. The comparator baseline scenario is of R_t remaining at 0.752 (black). Vertical dashed lines represent time-points of easing lockdown. (B), (C) The incident and cumulative deaths increase in all scenarios in which R_t increases.

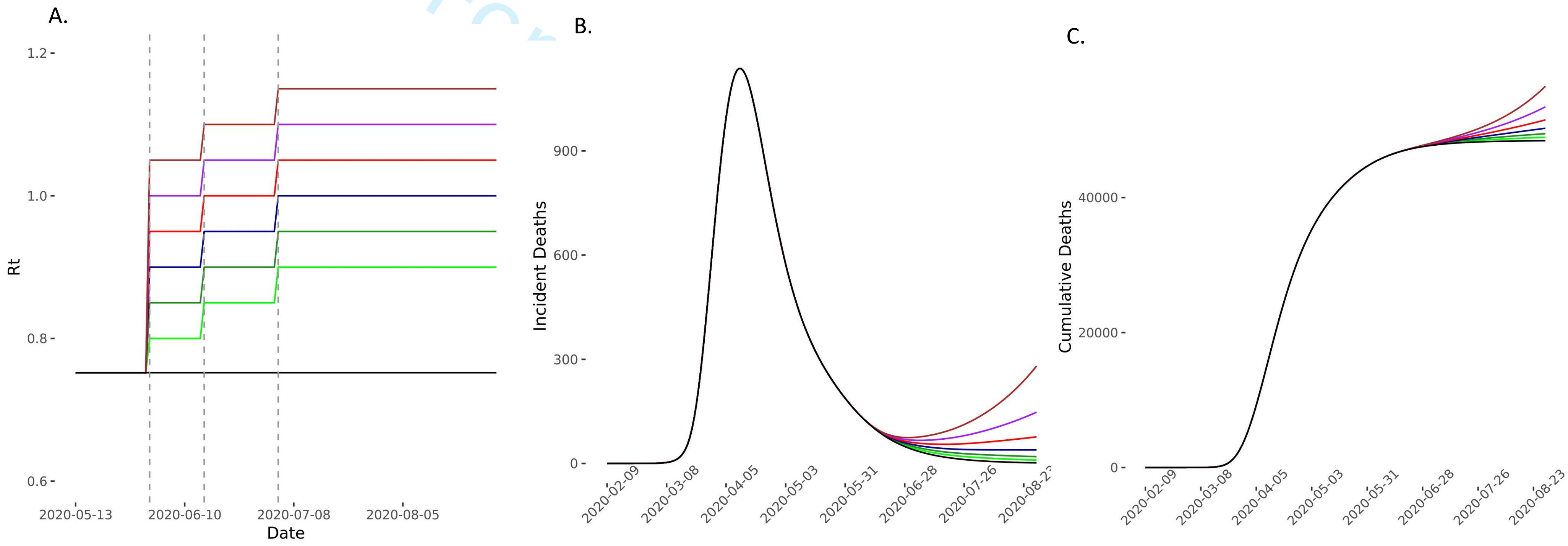

Figure 6. Predicted cases in scenarios of R_t increase on 1st June compared with baseline and elimination scenarios

(A) The model compared scenarios in which R_t increases to 0.80 (light green), 0.85 (green), 0.90 (dark blue), 0.95 (red), 1 (purple) and 1.05(brown) and then remains constant for the 90-day forecasting period. The comparator baseline scenario is of R_t remaining at 0.752 (black) and two elimination strategies of R_t reducing to 0.7 (yellow) and 0.6(light blue) were also considered. Vertical dashed lines represent time-points of easing lockdown. (B), (C) the incident and cumulative cases increase in all scenarios in which R_t increases and reduces in the two elimination scenarios.

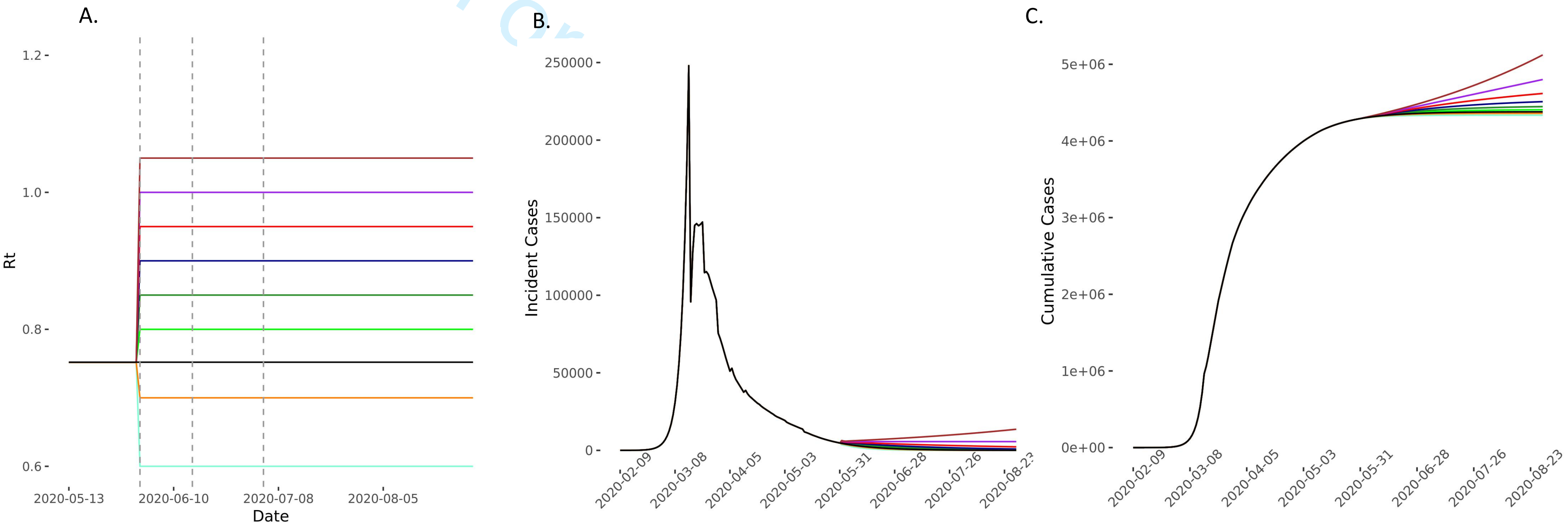

Figure 7. Predicted cases in scenarios of R_t increase on 1st June and 15th June compared with the baseline scenario

(A) The model compared scenarios in which R_t increases to 0.80 (light green), 0.85 (green), 0.90 (blue), 0.95 (red), 1 (purple) and 1.05(brown) and then further by 0.05 on the 15th June and then remaining constant for the 90-day forecasting period. The comparator baseline scenario is of R_t remaining at 0.752 (black). Vertical dashed lines represent time-points of easing lockdown. (B), (C) The incident and cases increase in all scenarios in which R_t increases.

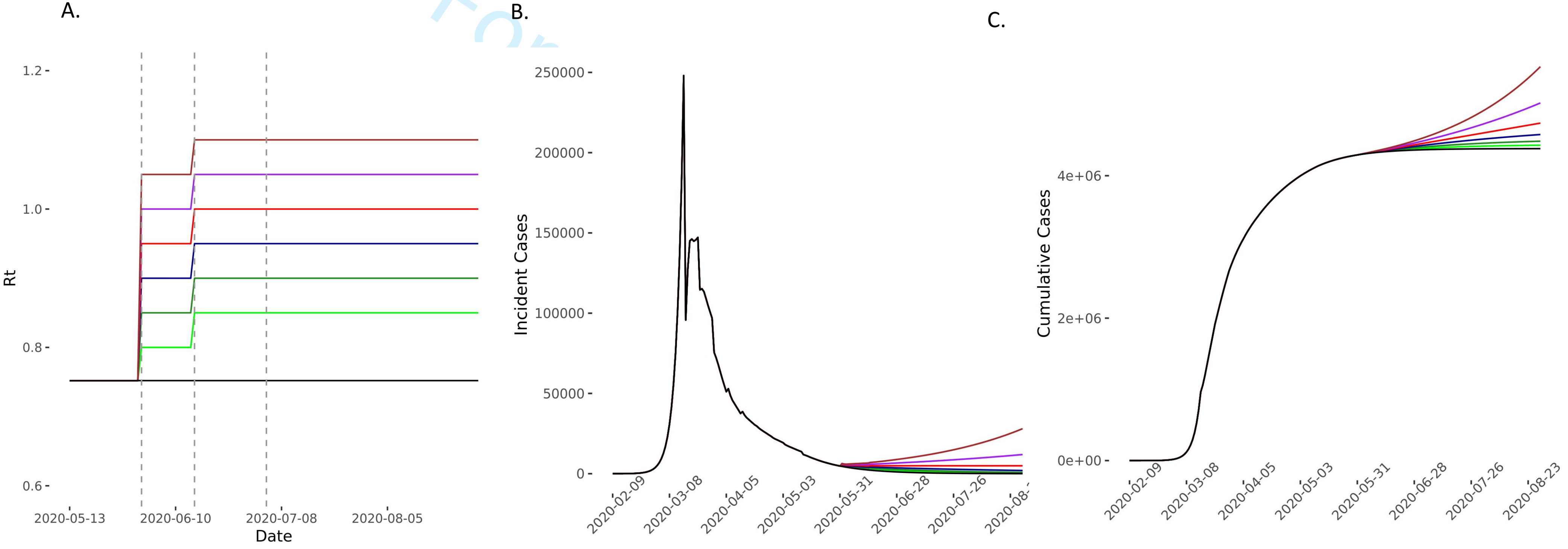

Figure 8. Predicted cases in scenarios of R_t increase on 1st June and 15th June and 4th July compared with the baseline scenario

(A) The model compared scenarios in which R_t increases to 0.80 (light green), 0.85 (green), 0.90 (blue), 0.95 (red), 1 (purple) and 1.05(brown) and then further by 0.05 on the 15th June and then again by 0.05 on the 3rd July before remaining constant for the 90-day forecasting period. The comparator baseline scenario is of R_t remaining at 0.752 (black). Vertical dashed lines represent time-points of easing lockdown. (B), (C) The incident and cumulative cases increase in all scenarios in which R_t increases.

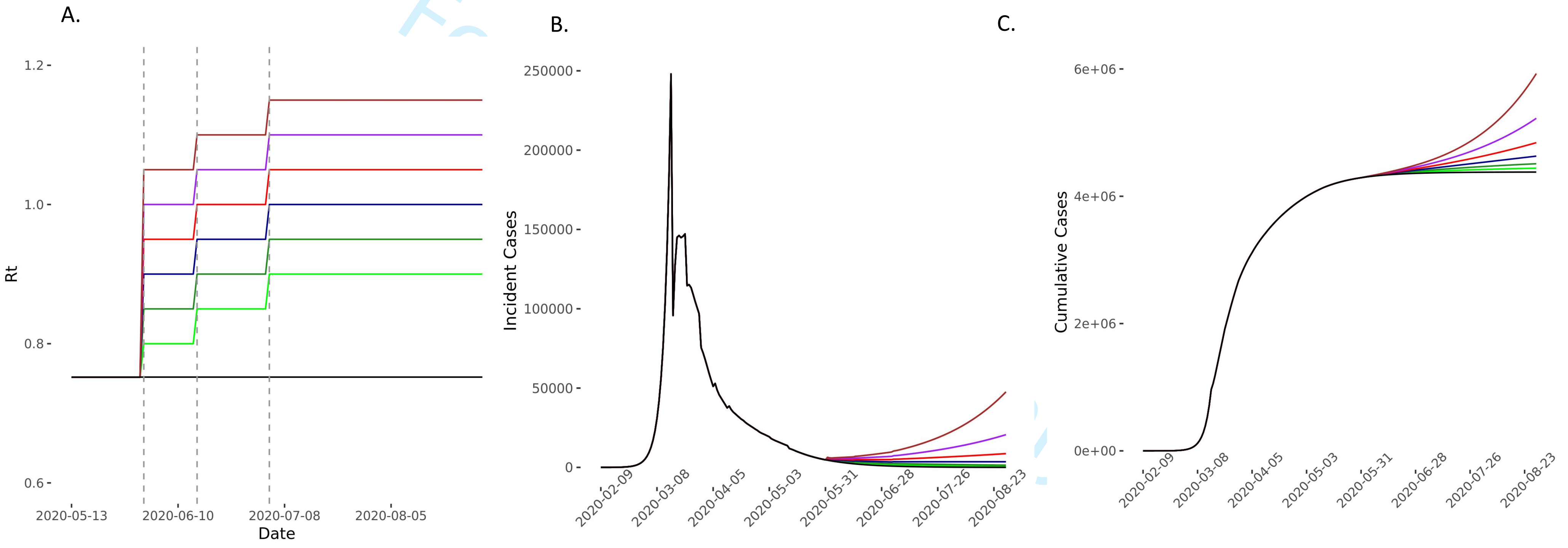

Figure 9. Predicted deaths in different scenarios of R_t increase on 1st June, 15th June and 4th July compared with the baseline scenario, and real observed death data from the ONS (light green).

The model compared scenarios in which R_t increases to different values on the 1st, 15th and 4th of July with real observed deaths (light green). The comparator baseline scenario is of R_t remaining at 0.752 (black). Vertical dashed lines represent time-points of easing lockdown. (B), (C) The incident and and cumulative deaths increase in all scenarios in which R_t increases. The daily deaths appear to fit best with the scenarios where R_ts were between 0.85 and 0.95 (dark blue, light blue, and purple) during this period.

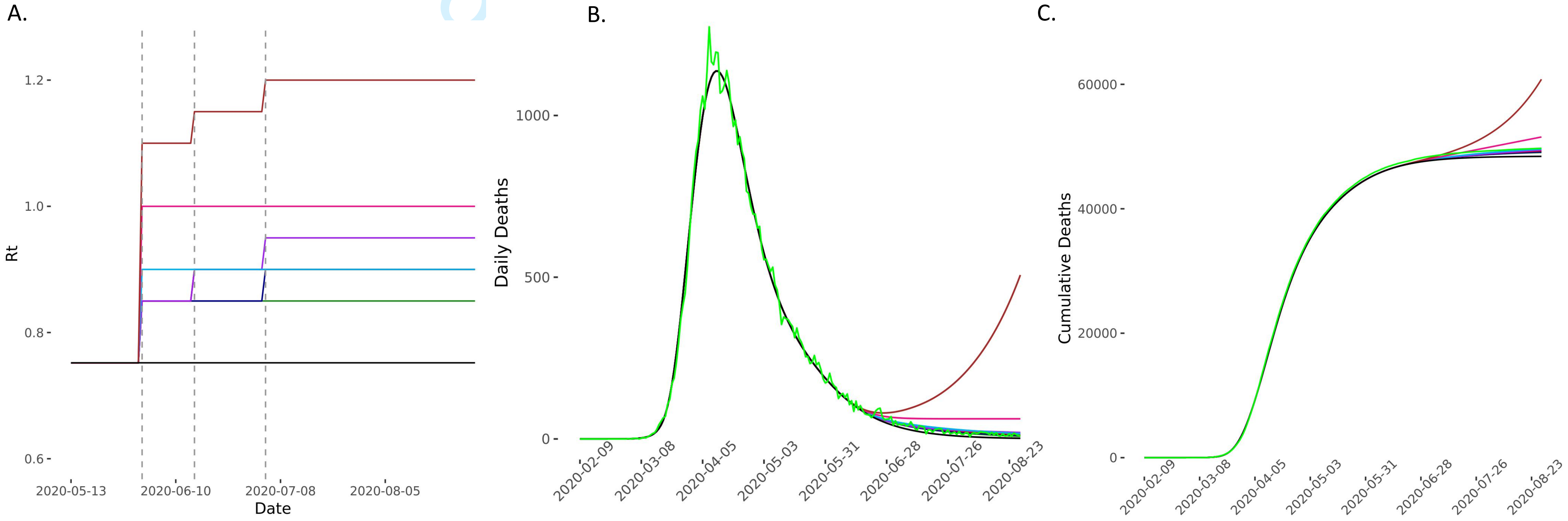

Figure 10: Estimated time-varying reproduction number (R_t) for England

The figure shows the R_t estimated from the recent ONS death data (up to September 11, 2020) with 95% credible intervals (grey) with a serial interval of mean 4.7 and SD 2.9 days. We see a gradual upward trend in R_t, with median R_t rising above 1 toward the end of July.

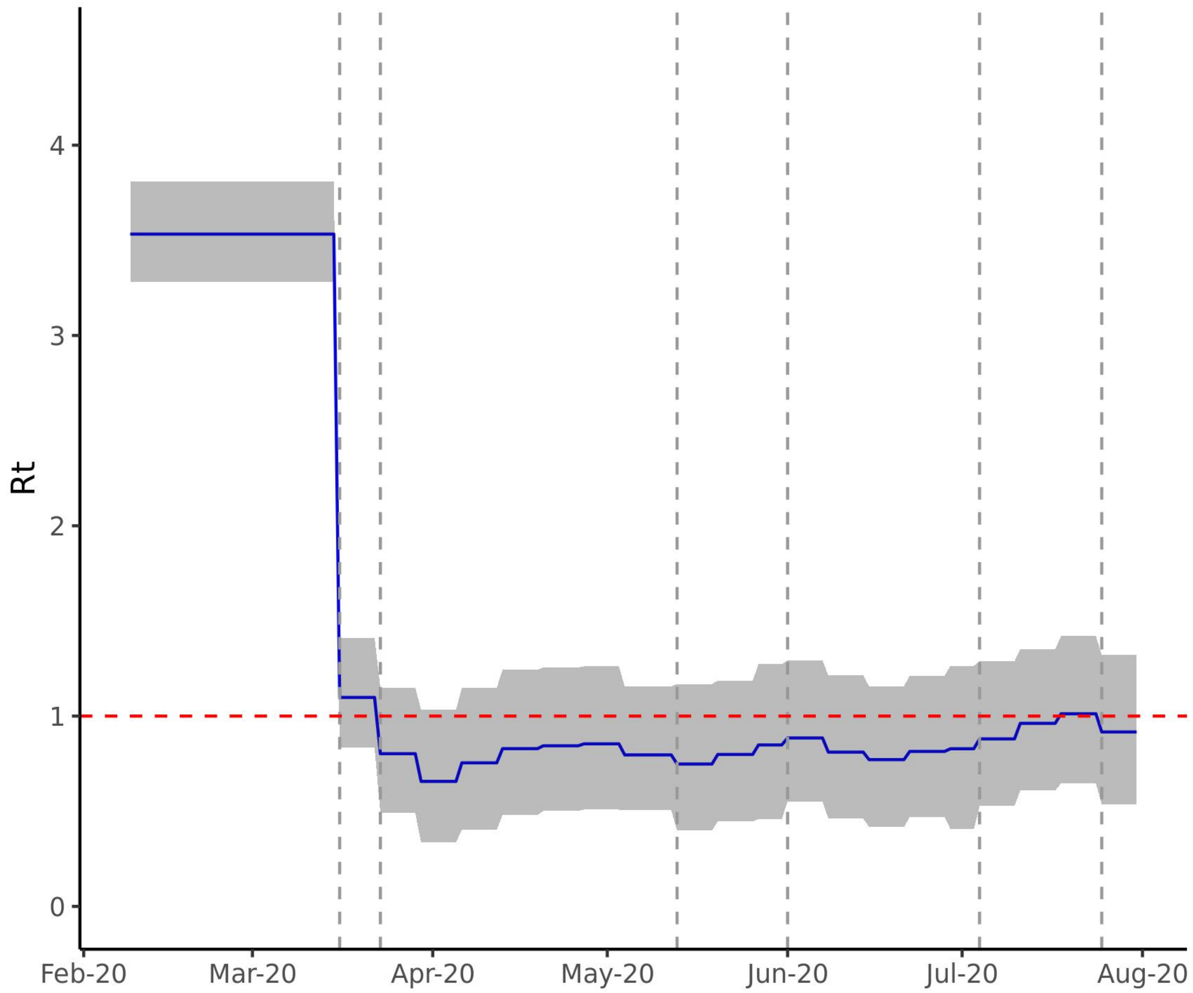

Figure 11: Predicted cases and deaths at different R_t values from current case numbers in England as of 21st September 2020

Figure 11 represents the predicted rise in cases based on different R_t values, and a serial interval of mean 4.7 and SD 2.9 days. The case numbers were calculated as a moving 7 day average from the Public Health England data of confirmed cases within England up to the 21st September. We project case, and death numbers (assuming an IFR of 1%) from these incident case numbers, using different scenarios of R_t. We note that case numbers are likely underestimates, as the testing system within England is currently running at capacity, and not everyone with symptoms is able to access tests.

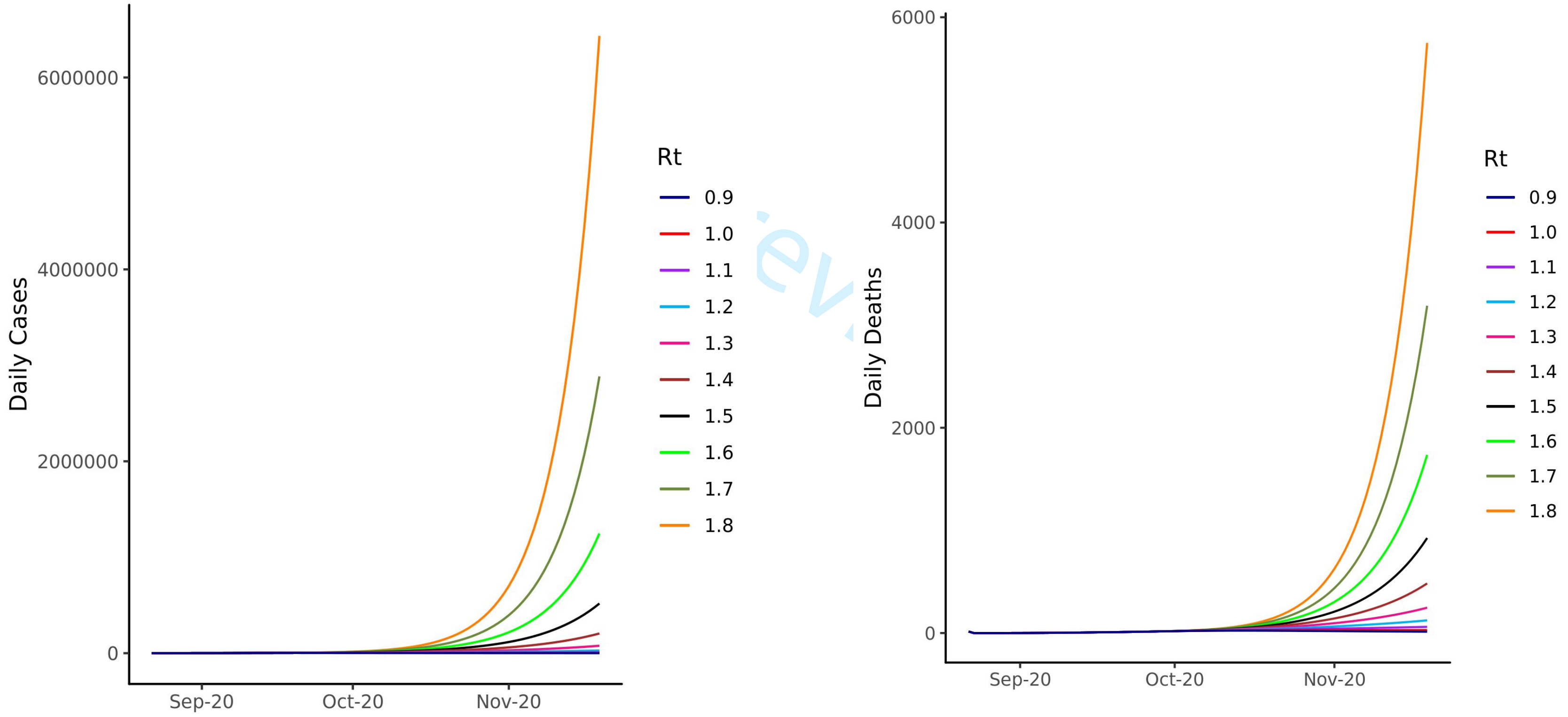

SUPPLEMENTARY MATERIALS

Modelling the impact of lockdown easing measures on cumulative COVID-19 cases and deaths in England

Ziauddeen H, PhD [1,2,3], Subramaniam N, BSc [1,2], Gurdasani D, PhD† [4].

Supplementary Table 1: Comparison of Bayesian models with different constraints on changes in R_t

Model	RMSE	EPLD	SE	diff_EPLD_model3	diff_SE_model3
model 1	38.0	-505.1	25.0	-1.5	0.5
model 2	28.3	-504.8	24.5	-1.2	0.9
model 3	28.0	-503.6	24.8	NA	NA

Supp. Table 1 represents model comparisons between Models 1-3, as specified in the text. RMSE represents the Root mean squared error between estimated and observed deaths for each model. EPLD represents the expected log pointwise predictive density which approximates leave-one-out (LOO) cross-validation. Less negative scores suggest better fit. SE is the standard error of EPLD. We assess the difference in EPLD between all models and the best performing model (Model 3 in this case), comparing the difference in EPLD (diff_EPLD_model3) with the standard error of the difference (diff_SE_model3). Although all three models appear comparable in performance, Model 3 appears to show the best fit with the lowest RMSE, and the least negative EPLD.

For peer review only

Supplementary Table 2: Comparison of models excluding specific change points for R_t

change points removed	RMSE	EPLD	SE	diff_EPLD	diff_SE
16th March	118.1	-576.0	26.1	-70.9	5.3
23rd March	44.6	-506.7	25.2	-1.6	1.0
13th May	40.2	-504.8	25.0	0.3	0.4
1st June	38.1	-505.2	25.0	-0.1	0.0
None (all included)	38.0	-505.1	25.0	NA	NA

Supp. Table 2 represents model comparisons between Models that constrain Rt at each of the 4 hypothesised change points at which point social distancing or lockdown measures were introduced (16th March and 23rd March), or when lockdown measures were eased (13th May and 1st June). The first column represents the change point left out in each model, with the last model with all three change points being the comparator, as specified in the text. RMSE represents the Root mean squared error between estimated and observed deaths for each model. EPLD represents the expected log pointwise predictive density which approximates leave-one-out (LOO) cross-validation. Less negative scores suggest better fit. SE is the standard error of EPLD. We assess the difference in EPLD between all models and the model with all three change points, comparing the difference in EPLD (diff_EPLD) with the standard error of the difference (diff_SE). The model leaving out 16th March as a change point, i.e. constraining Rt to remain constant at this point appears to adversely impact fit the most.

For peer review only

Supplementary Table 3: Cumulative cases and deaths in lockdown easing scenarios in primary model

Rt 1st June	Rt 15th June	Rt 4th July	Cumulative cases	Cumulative deaths	Cases difference from baseline	Death difference from baseline	RMSE
0.752	0.752	0.752	4411594(4199223-4639250)	48501(46170-50989)	0(0,0)	0(0,0)	
0.6	0.6	0.6	4364386(4162299-4580834)	48006(45783-50386)	-44302(-84684--18600	-462(-884--194)	25.3
0.65	0.65	0.65	4374559(4170697-4593499)	48115(45875-50523)	-33831(-64668--14204)	-350(-669--147)	24.9
0.7	0.7	0.7	4391027(4183302-4610584)	48286(46007-50696)	-19968(-38168--8384)	-204(-389--86)	24.4
0.75	0.75	0.75	4410590(4198499-4637531)	48494(46163-50977)	-908(-1736--381)	-9(-17--4)	23.9
0.75	0.75	0.8	4415149(4201945-4645342)	48518(46186-51016)	3069(1285-5890)	19(8-37)	23.8
0.75	0.8	0.8	4424153(4209052-4658126)	48612(46255-51149)	11497(4814-22058)	102(43-195)	23.7
0.75	0.8	0.85	4430866(4213721-4668154)	48654(46293-51225)	18197(7620-34906)	145(61-278)	23.6
0.8	0.8	0.8	4439684(4219884-4679358)	48771(46375-51380)	26447(11105-50549)	257(108-492)	23.4
0.8	0.8	0.85	4447876(4225283-4692920)	48827(46413-51458)	34303(14397-65598)	308(129-589)	23.3
0.8	0.85	0.85	4461240(4232489-4716984)	48954(46492-51680)	47523(19934-90933)	431(181-825)	23.2
0.8	0.85	0.9	4474630(4243279-4736698)	49036(46538-51811)	60851(25519-116475)	508(213-972)	23.1
0.85	0.85	0.85	4481739(4247839-4745973)	49166(46614-52010)	67576(28376-129149)	632(265-1208)	23.0
0.85	0.85	0.9	4498639(4257710-4770487)	49246(46692-52138)	83109(34888-158891)	722(303-1379)	23.0
0.85	0.9	0.9	4521199(4273493-4806484)	49446(46808-52428)	104334(43782-199536)	907(381-1733)	23.1
0.85	0.9	0.95	4547931(4291851-4848863)	49592(46917-52640)	130823(54887-250256)	1043(438-1994)	23.2
0.9	0.9	0.9	4549190(4292969-4850945)	49730(47007-52865)	132381(55595-252977)	1173(493-2240)	23.3
0.9	0.9	0.95	4579138(4311424-4905124)	49887(47120-53118)	163082(68471-311726)	1330(559-2542)	23.5
0.9	0.95	0.95	4613802(4328984-4965451)	50162(47308-53602)	197988(83107-378532)	1610(676-3077)	24.3
0.9	0.95	1	4667450(4358502-5053567)	50397(47439-54007)	250499(105129-479023)	1848(776-3534)	25.2
0.95	0.95	0.95	4655308(4352263-5032669)	50517(47499-54226)	239051(100411-456749)	1971(828-3764)	25.3
0.95	0.95	1	4718132(4385399-5139609)	50790(47648-54680)	299596(125815-572543)	2246(943-4290)	26.6
0.95	1	1	4779022(4412370-5246023)	51226(47883-55381)	358354(150465-684935)	2672(1122-5106)	28.8
0.95	1	1.05	4880364(4464544-5437916)	51650(48103-56116)	462002(193954-883178)	3087(1296-5899)	31.8
1	1	1	4840595(4444280-5359379)	51743(48160-56290)	421310(177012-804811)	3174(1334-6060)	31.4
1	1	1.05	4959206(4498273-5586656)	52235(48368-57131)	540234(226938-1032145)	3649(1533-6970)	35.2
1	1.05	1.05	5055758(4548961-5767421)	52892(48712-58297)	641220(269327-1225202)	4303(1808-8220)	40.2
1	1.05	1.1	5264669(4632979-6143068)	53598(49059-59632)	844596(354706-1613979)	5018(2108-9587)	47.2
1.05	1.05	1.05	5156984(4594880-5946919)	53594(49059-59623)	741957(311832-1416940)	5017(2109-9578)	45.3
1.05	1.05	1.1	5397044(4692140-6391841)	54411(49421-61165)	974252(409410-1860772)	5833(2452-11138)	53.5
1.05	1.1	1.1	5574165(4770085-6732948)	55401(49905-63038)	1150799(483559-2198106)	6843(2876-13067)	62.8
1.05	1.1	1.15	5969381(4946974-7481001)	56609(50491-65190)	1546934(649955-2954983)	8065(3390-15402)	76.7
1.1	1.1	1.1	5744209(4843263-7044331)	56428(50400-64839)	1317940(554129-2516086)	7878(3313-15037)	71.5
1.1	1.1	1.15	6189495(5035202-7885232)	57860(50954-67461)	1768512(743504-3376542)	9269(3898-17692)	87.4
1.1	1.15	1.15	6501607(5163364-8475727)	59458(51650-70470)	2081127(874883-3973567)	10834(4556-20682)	103.2
1.1	1.15	1.2	7272289(5484637-9955130)	61543(52551-74465)	2846203(1196439-5434639)	12908(5427-24642)	128.7

Supplementary Table 3 represents the estimated cumulative deaths, cumulative cases, and excess deaths and cases in different scenarios of changing Rt at points of easing lockdown in comparison with the baseline scenario of Rt remaining constant at 0.752.

For peer review only

Supplementary Table 4: Cumulative cases and deaths in lockdown easing scenarios in model with long serial interval

Rt 1st June	Rt 15th June	Rt 4th July	Cumulative cases	Cumulative deaths	Cases difference from baseline	Death difference from baseline
0.691	0.691	0.691	4404236(4183512-4622330)	48411(45990-50805)	0(0-0)	0(0-0)
0.6	0.6	0.6	4371866(4158398-4583033)	48078(45733-50400)	-32402(-52309--17783)	-330(-533--181)
0.65	0.65	0.65	4387428(4171118-4603369)	48241(45863-50609)	-16515(-26661--9065)	-166(-268--91)
0.7	0.7	0.7	4408635(4186726-4627236)	48451(46022-50849)	4158(2283-6712)	41(23-66)
0.7	0.7	0.75	4413466(4190714-4632818)	48487(46047-50893)	8904(4865-14430)	75(41-121)
0.7	0.75	0.75	4421929(4197326-4644215)	48571(46126-50997)	17612(9599-28584)	160(87-259)
0.7	0.75	0.8	4428891(4202474-4652651)	48618(46159-51054)	24664(13432-40046)	206(112-334)
0.75	0.75	0.75	4436254(4207602-4661170)	48717(46226-51175)	31769(17441-51275)	307(168-494)
0.75	0.75	0.8	4444061(4214414-4673025)	48768(46266-51240)	39710(21767-64168)	358(197-579)
0.75	0.8	0.8	4456057(4225564-4689908)	48884(46358-51403)	51923(28426-84007)	473(259-765)
0.75	0.8	0.85	4468509(4233843-4705662)	48955(46423-51508)	63758(34875-103225)	544(298-881)
0.8	0.8	0.8	4474042(4238163-4713852)	49060(46515-51637)	69645(38242-112391)	652(358-1051)
0.8	0.8	0.85	4487239(4247137-4731986)	49147(46570-51756)	82925(45486-133921)	732(402-1180)
0.8	0.85	0.85	4504368(4260726-4758965)	49297(46674-51983)	100288(54965-162081)	887(487-1433)
0.8	0.85	0.9	4524968(4276399-4786847)	49405(46756-52128)	120111(65789-194244)	997(546-1610)
0.85	0.85	0.85	4528097(4278796-4789875)	49515(46843-52296)	122997(67554-198451)	1107(609-1785)
0.85	0.85	0.9	4550519(4295317-4824987)	49639(46934-52475)	145167(79670-234360)	1229(675-1983)
0.85	0.9	0.9	4575600(4314157-4857930)	49864(47104-52757)	170201(93348-274900)	1441(791-2326)
0.85	0.9	0.95	4608999(4336767-4903946)	50032(47229-53038)	203356(111471-328606)	1608(883-2597)
0.9	0.9	0.9	4605943(4334310-4898052)	50138(47312-53190)	200055(109912-322694)	1716(944-2766)
0.9	0.9	0.95	4643792(4360620-4952873)	50326(47454-53431)	237006(130136-382485)	1902(1046-3067)
0.9	0.95	0.95	4680570(4385671-5007504)	50621(47645-53806)	273569(150145-441642)	2192(1204-3535)
0.9	0.95	1	4735012(4422134-5091796)	50870(47843-54169)	328896(180416-531139)	2446(1343-3946)
0.95	0.95	0.95	4720210(4412771-5069359)	50965(47911-54323)	313876(172518-506126)	2540(1397-4093)
0.95	0.95	1	4781115(4451857-5160478)	51266(48080-54750)	375333(206196-605461)	2822(1552-4549)
0.95	1	1	4836432(4486836-5241471)	51661(48368-55340)	429433(235844-692907)	3219(1770-5191)
0.95	1	1.05	4928929(4549055-5385960)	52032(48613-55941)	521422(286255-841597)	3603(1980-5811)
1	1	1	4892268(4526735-5326989)	52097(48655-56020)	485265(266854-782184)	3667(2018-5906)
1	1	1.05	4995150(4589022-5491882)	52523(48926-56643)	587186(322775-946757)	4092(2252-6593)
1	1.05	1.05	5076092(4640904-5614490)	53078(49274-57480)	668188(367218-1077551)	4639(2552-7476)
1	1.05	1.1	5227156(4734604-5849954)	53653(49678-58359)	820638(450864-1323717)	5217(2869-8409)
1.05	1.05	1.05	5154698(4689409-5733251)	53657(49684-58359)	747364(411252-1204170)	5221(2876-8408)
1.05	1.05	1.1	5321327(4792479-5997797)	54309(50099-59370)	915842(503787-1475921)	5860(3227-9437)
1.05	1.1	1.1	5444111(4858418-6188703)	55069(50551-60585)	1038372(571090-1673570)	6615(3642-10657)
1.05	1.1	1.15	5698707(4995152-6588038)	55936(51099-61889)	1290002(709312-2079494)	7480(4117-12052)
1.1	1.1	1.1	5558967(4921706-6371716)	55831(51060-61736)	1153037(634971-1857036)	7382(4069-11884)
1.1	1.1	1.15	5838364(5075136-6811946)	56779(51632-63173)	1430388(787494-2304058)	8336(4593-13420)
1.1	1.15	1.15	6021434(5176394-7116504)	57812(52276-64824)	1617286(890276-2605292)	9384(5170-15109)
1.1	1.15	1.2	6430941(5404442-7783304)	59109(53001-66839)	2030763(1117659-3271715)	10673(5879-17187)

Supplementary Table 4 represents the estimated cumulative deaths, cumulative cases, and excess deaths and cases in different scenarios of changing Rt at points of easing lockdown in comparison with the baseline scenario of Rt remaining constant at 0.691.

For peer review only

Supplementary Figure 1: Distribution of R-hat for parameters from final model

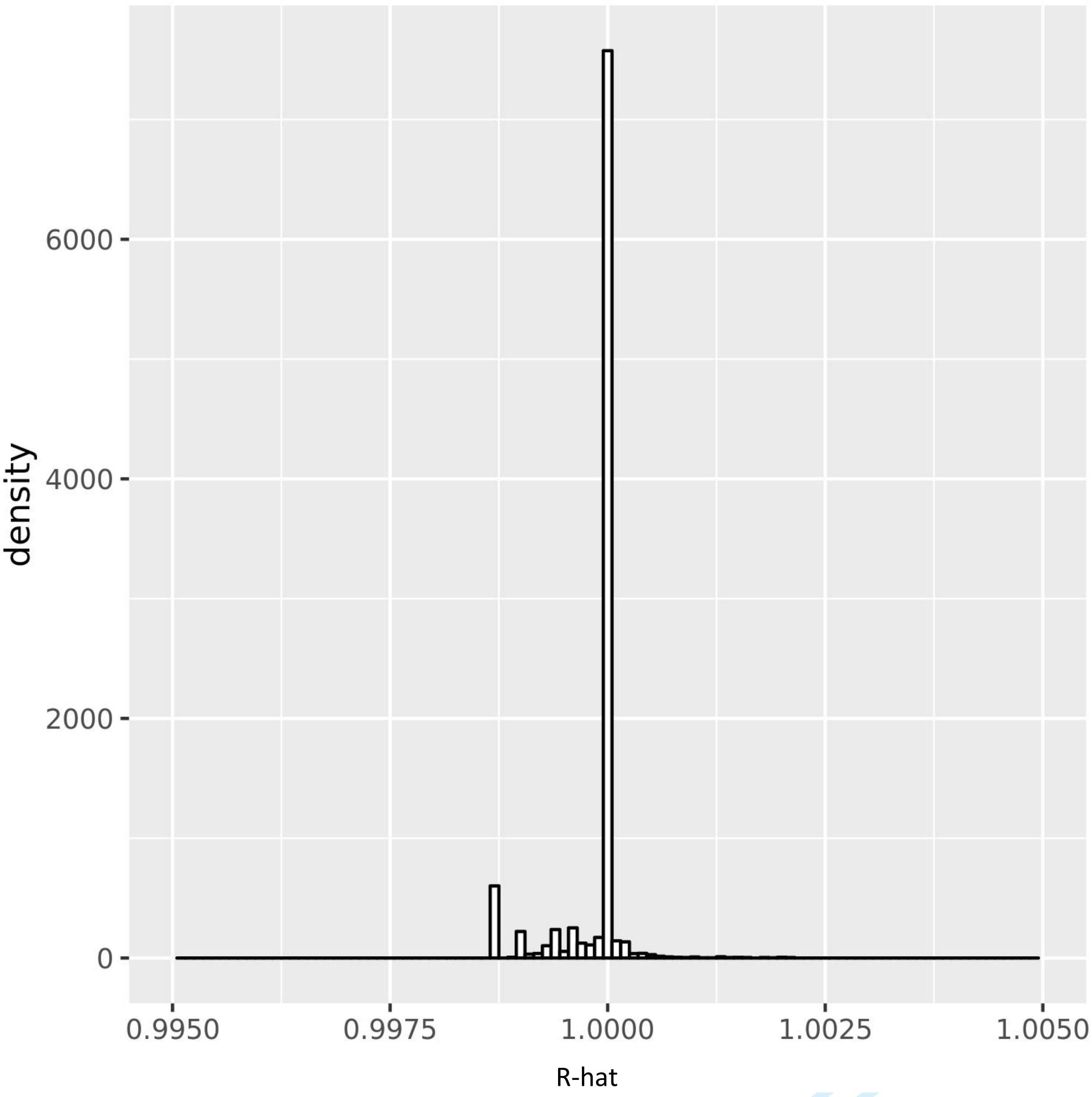

Supplementary Figure 1 represents the estimated R-hat for parameters of the final model with duplicates removed. The mean R-hat was 1.000057. An R-hat near 1 suggests that between-chain variance for a given parameter is equal to the within-chain variance, suggesting convergence of the model. All values were well below 1.05.

Supplementary Figure 2: Pareto shape parameter k distribution for final model

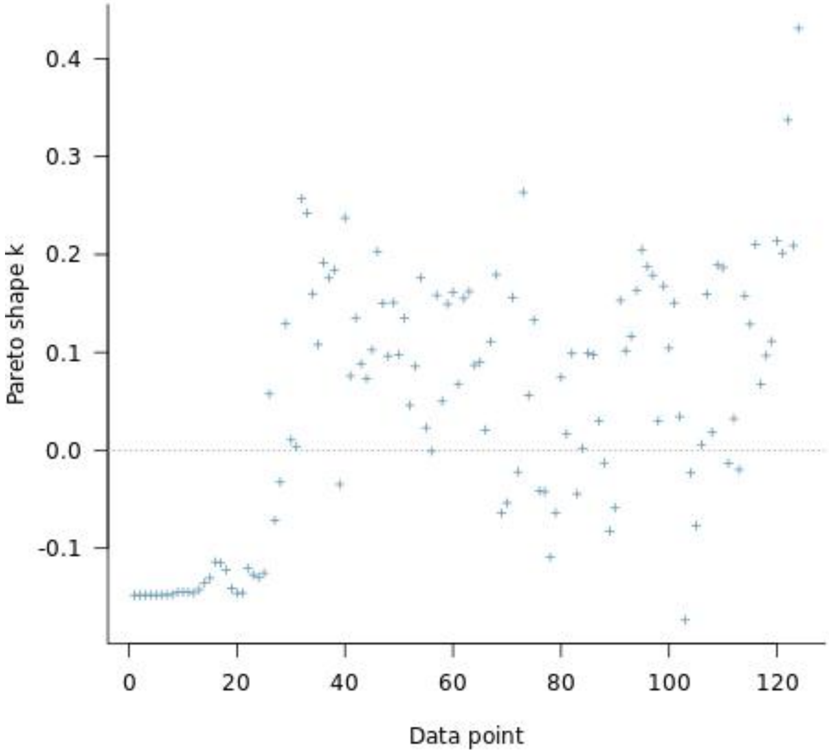

The estimated shape parameter k of the generalized Pareto distribution can be used to assess the reliability of the estimate from approximations of Leave-one-out cross-validation (LOO). The k shape values are all below 0.5, suggesting our estimates are reliable.

eview only

Supplementary Figure 3: Rt estimates with broad and uninformative priors

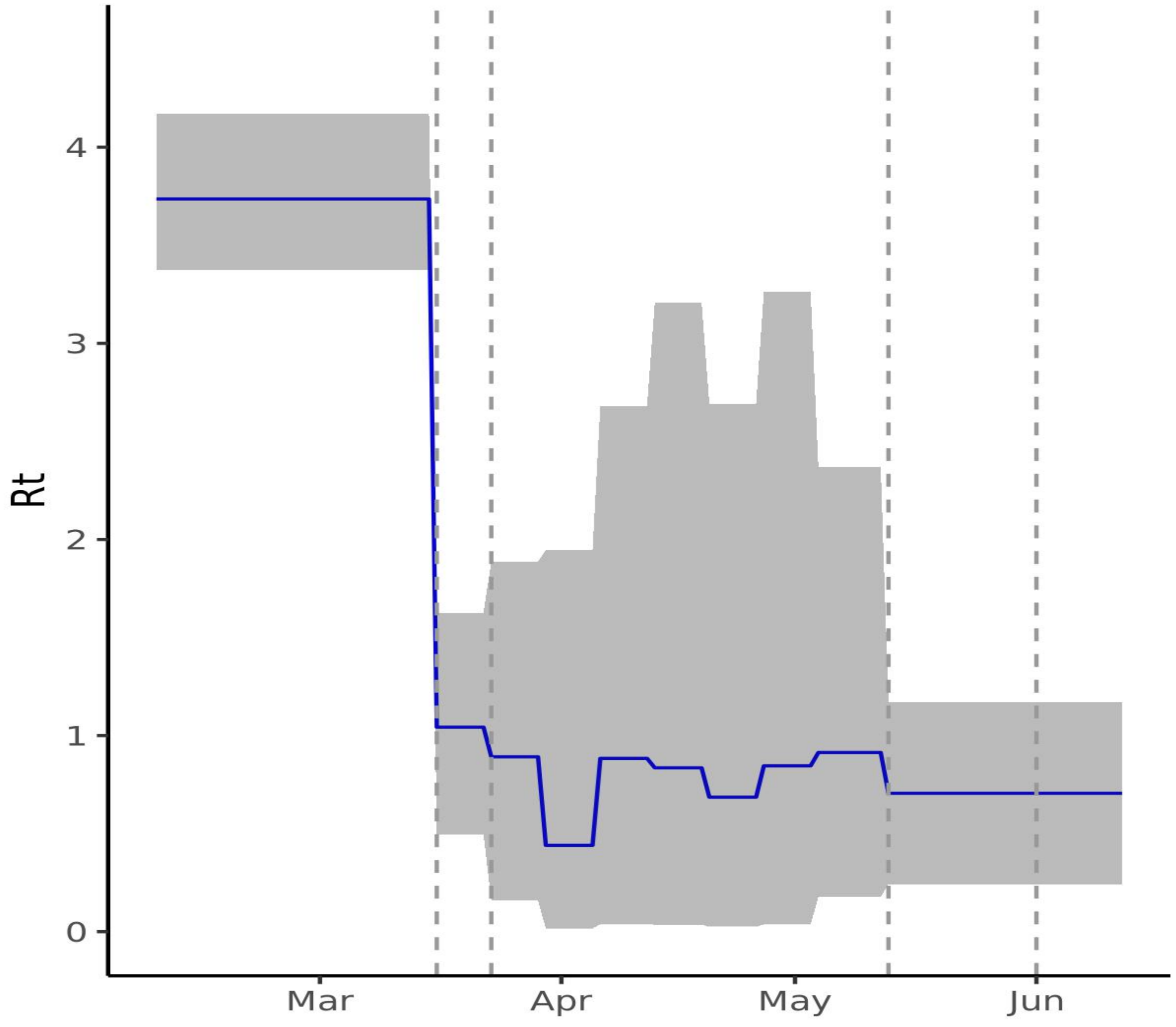

Supplementary Figure 3. Represents estimates of Rt when uninformative priors are used for estimation. We find that although uncertainty is greater around estimates, median estimates, and patterns of changes are similar as for the original model for all time intervals, suggesting that these are not constrained by specification of the prior in the final model.

Supplementary Figure 4: Estimated reproduction number in model with longer serial interval

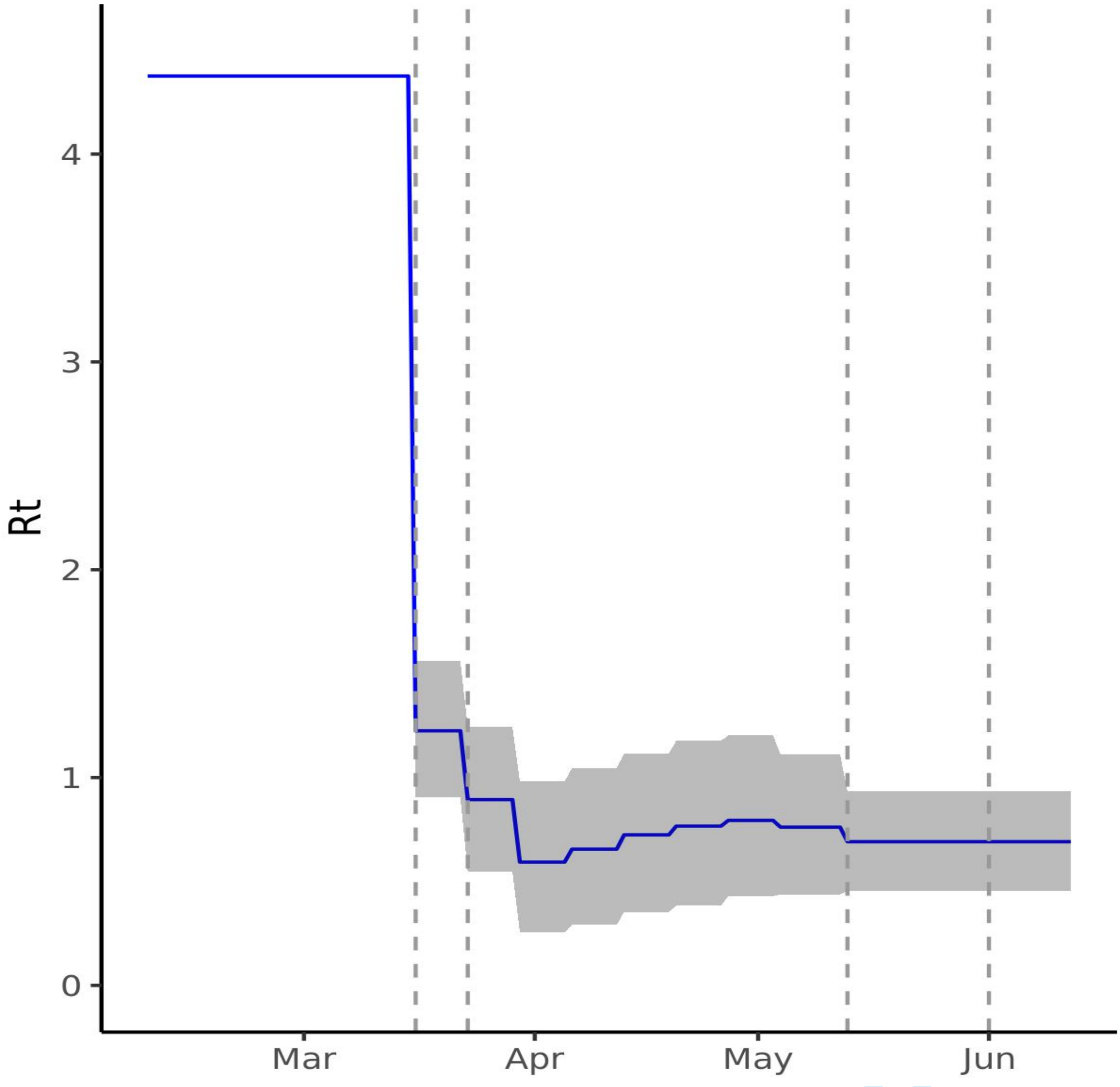

The figure shows the R_t estimated by a model with a a serial interval of mean 6.5 and coefficient of variation of 0.72. While estimates of R0 are higher in this model, estimates during other time intervals following lockdown are very similar to our primary model. 95% credible intervals are represented by grey bands.

Supplementary Figure 5: Predicted and observed deaths in model with longer serial interval

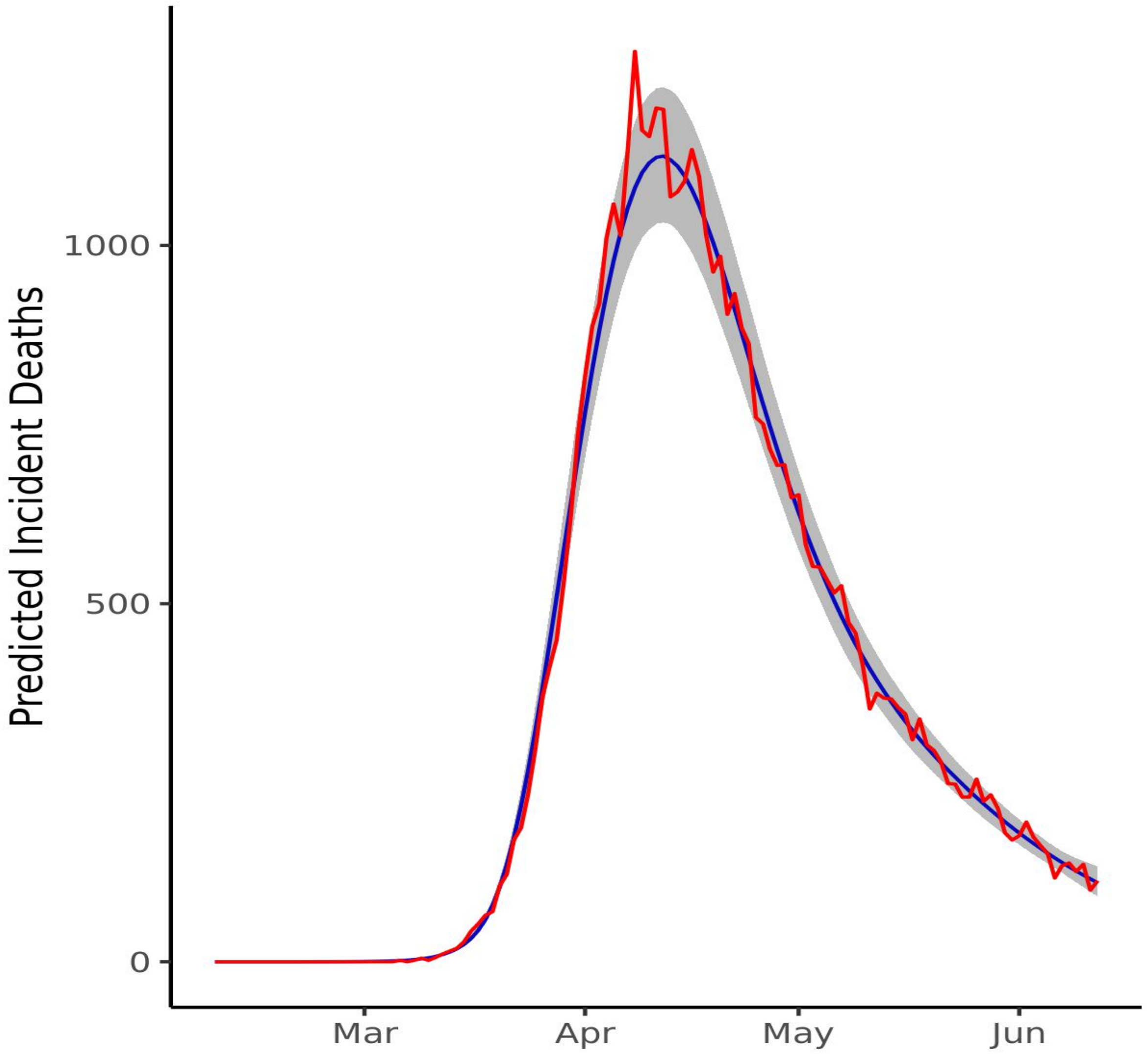

Daily deaths predicted by a model specifying longer serial intervals (blue) with 95% credible intervals (grey) show a good fit to the observed deaths from the ONS (red)
